# Design, Synthesis, In Vitro and In Silico Biological Evaluation of New Pyridine-2,5-Dicarboxylates Esters Bearing Natural Source Fragments as Anti-Trypanosomatid Agents

**DOI:** 10.3390/pharmaceutics17101271

**Published:** 2025-09-28

**Authors:** Luis M. Sánchez-Palestino, Adriana Moreno-Rodríguez, Diana V. Navarrete-Carriola, Marlet Martínez-Archundia, Marhian López-Vargas, Liliana Argueta-Figueroa, Lenci K. Vázquez-Jiménez, Alma D. Paz-González, Eyra Ortiz-Pérez, Michael P. Doyle, Gildardo Rivera

**Affiliations:** 1 Laboratorio de Biotecnología Farmacéutica, Centro de Biotecnología Genómica, Instituto Politécnico Nacional, Reynosa 88710, Mexico; lsanchezp2000@alumno.ipn.mx (L.M.S.-P.); dnavarretec1900@alumno.ipn.mx (D.V.N.-C.); lenka.18@hotmail.com (L.K.V.-J.); apazg@ipn.mx (A.D.P.-G.); eortizp@ipn.mx (E.O.-P.); 2 Department of Chemistry, University of Texas at San Antonio, One UTSA Circle, San Antonio, TX 78249, USA; 3 Laboratory for the Design and Development of New Drugs and Biotechnological Innovation, Escuela Superior de Medicina, Instituto Politécnico Nacional, Plan de San Luis y Díaz Mirón, Ciudad de México 11340, Mexico; mtmartineza@ipn.mx; 4 Laboratorio de Estudios Epidemiológicos, Clínicos, Diseños Experimentales e Investigación, Facultad de Ciencias Químicas, Universidad Autónoma “Benito Juárez” de Oaxaca, Avenida Universidad S/N, Ex Hacienda Cinco Señores, Oaxaca 68120, Mexico; augl770623.fmc@uabjo.mx (A.M.-R.); marhianlv9@gmail.com (M.L.-V.); 5 SECIHTI—Tecnológico Nacional de México, Instituto Tecnológico de Toluca, Metepec 52149, Mexico; liliana.argueta@secihti.mx

**Keywords:** pyridine hybrid derivatives, 1,2,3-triazine 1-oxide, natural products, antiprotozoal agents, ADMET properties

## Abstract

**Background**: Chagas disease and leishmaniasis remain public health concerns. Despite the existence of approved medications for the treatment of these diseases, most patients discontinue treatment due to long drug regimens and/or the severe side effects of these drugs. This leads to treatment failure and potential future drug resistance. Therefore, the search for new molecules with trypanocidal activity, low cytotoxicity, and high selectivity is essential to address this challenge. **Methods**: In this work, three series (a, b, and c) of pyridine-2,5-dicarboxylate esters were synthesized using different β-keto-esters bearing naturally occurring fragments and 1,2,3-triazine-1-oxides via the inverse electron demand Diels–Alder (IEDDA) reaction. The structural elucidation of the compounds was performed using NMR (^1^H and ^13^C) and HRMS, and the crystal structure of compound **6a** was also obtained. Furthermore, a biological assay was performed for all synthesized and characterized compounds to determine their cytotoxicity against *Trypanosoma cruzi*, *Leishmania mexicana*, and the J774.2 macrophage cell line. Finally, the in silico determination of their pharmacokinetic and toxicological properties was performed using the SwissADME and ProTox 3.0 platforms. **Results**: Compounds **3a**, **4a**, **5a**, **4b**, and **8c** had the highest anti-*Trypanosoma cruzi* activity against both strains (IC_50_ ≤ 56.68 µM). Compounds **8b**, **10a**, **9b**, and **12b** had considerable leishmanicidal activity against *Leishmania mexicana* against both strains (IC_50_ ≤ 161.53 µM). Furthermore, in silico prediction of ADMET properties suggest that these pyridine compounds possess good pharmacokinetic profile. The results are also consistent with low in vitro cytotoxicity and high selectivity. **Conclusions**: The synthesized pyridine-2,5-dicarboxylate esters have promising activity against *Trypanosoma cruzi* and *Leishmania mexicana*, with low cytotoxicity and good drug-like properties, suggesting that these compounds are potential candidates for further evaluation as new treatments for Chagas disease and leishmaniasis.

## 1. Introduction

Trypanosomatidae is a family of obligatory protozoan parasites predominantly restricted to invertebrate hosts (monoxenus life cycle). However, trypanosomatids belonging to the genus Trypanosoma and Leishmania possess a dixenous behavior, where a zoonotic or anthroponotic life cycle is needed. The genera Trypanosoma and Leishmania are in the spotlight due to their role as human pathogens causing devastating human diseases, including Human African Trypanosomiasis (Sleeping sickness), Chagas disease, and Leishmaniasis [1].

*Trypanosoma cruzi* (*T. cruzi*) is the etiological agent of Chagas disease, also known as American Trypanosomiasis, which is primarily transmitted by triatomine bugs. However, other important transmission routes include oral, congenital, blood transfusion, and organ transplantation. Global estimates suggest that approximately six million people are infected, with up to 30% likely to experience severe, life-threatening symptoms affecting the heart, digestive system, and nervous system [2]. Moreover, one million people are believed to be infected with *T. cruzi*, and 7700 new cases are reported per year [3].

Currently, the treatment for Chagas disease involves the use of two nitrogen-heterocyclic drugs, Benznidazole and Nifurtimox [4,5]. Both drugs are primarily effective during the early stages of the acute phase. However, their efficacy diminishes in older individuals and during the chronic phase of the disease. Other factors, such as the host’s immune response and the strain of the parasite, may also affect the effectiveness of the drugs. Furthermore, the treatment costs, coupled with the inability to work due to the symptoms of Chagas disease, contribute to significant global economic losses, particularly in endemic regions [4].

Leishmaniasis disease is caused by various species belonging to *Leishmania* spp. genus. Leishmaniasis could be presented mainly in two forms: (1) visceral leishmaniasis (or kala-azar), which causes 70,000 new cases per year and is the most serious leishmaniasis form; (2) cutaneous leishmaniasis, which is the most common leishmaniasis form, causing ulcers and skin lesions, and it is estimated that around 800,000 new cases occur annually [2].

*Leishmania mexicana* (*L. mexicana*) is mainly transmitted by infected female phlebotomine sandflies of the genera *Phlebotomus* and *Lutzomyia*, the latter of which is present in the Americas. Two stages in the life cycle have been identified in *Leishmania* spp.: promastigote (flagellated), which is found in sand flies, and amastigote (non-flagellated) [6]. As stated by the World Health Organization (WHO), the treatment for Leishmaniasis is based on the type of disease, parasite species, and geographical location, and treatment follows the health guidelines according to each country [7]. Pentavalent antimonials [Sb(V)], such as Pentostam and Glucantime, are used in all clinical forms of leishmaniasis. Other treatment options are amphotericin B, lipidic formulations of amphotericin B, paromomycin, pentamidine isethionate, miltefosine, and antifungals such as ketoconazole and itraconazole [6,8].

Due to the side effects of drugs used in the treatment of Chagas disease and Leishmaniasis, there is an urgent need to develop new agents that are highly effective therapeutically with low cytotoxicity. This would help avoid treatment interruptions, which can lead to treatment failure.

**Figure 1:**
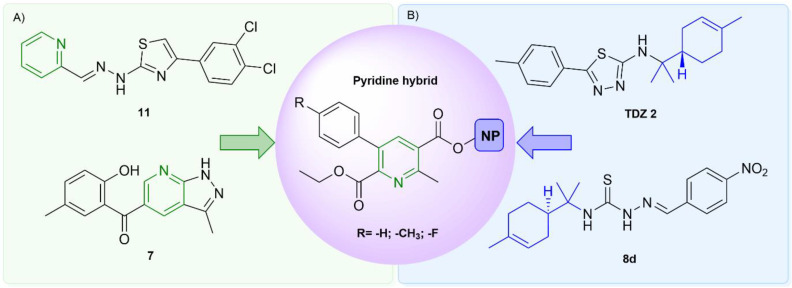
Pyridine and secondary metabolites of natural products with trypanocidal activities. (**A**) Pyridine compounds with trypanocidal (**11**, IC_50_ = 4 µM) and leishmanicidal activity (**7**, IC_50_ = 31 µM). (**B**) Molecules containing natural product scaffolds with trypanocidal (TDZ 2, IC_50_ = 1.3 µM) and leishmanicidal activity (**8d**, IC_50_ = 2.4 µM).

On the other hand, natural products (NPs) are a major source of molecules, diverse in structure and biological applications [9,10]. In general, NPs are characterized by aliphatic fragments (chains and ring systems), but arene systems are also present. Moreover, NPs often have more than one chiral center, which confers greater complexity, and the oxygen atom content is higher compared to nitrogen [11,12]. Terpenoids are one of the most abundant and diverse groups of naturally occurring compounds, and most of them are derived from plants. Their biological activities include antitumor, anti-inflammatory, antibacterial, antiviral, and antimalarial, among others [13]. Some molecules containing terpenoid scaffolds have antichagasic (TDZ 2) [14] and antileishmanial (**8d**) activities [15] (Figure 1). Based on their favorable pharmacokinetic profile and broad biological applications, the search for terpenoids with trypanocidal activity appears to be a suitable path.

Additionally, the pyridine ring is an isostere of benzene and is found in secondary metabolites. The pharmaceutical industry uses the pyridine scaffold in many existing drugs. Besides, pharmacological activities of many pyridine compounds have been reported, such as antibacterial, anticancer, antifungal, anti-inflammatory, antitubercular, antichagasic, antimalarial, and anti-amoebic effects, among others [13]. The physicochemical properties of the pyridine ring, including its polarity, ionization capacity, basicity, solubility, and hydrogen-bond forming capability, contribute to its continued use in drugs [16]. Recent studies have also demonstrated trypanocidal activities of pyridine compounds against *T. cruzi*, such as compound **11** [17] and against *L. mexicana*, compound **7** [18] (Figure 1). Recently, our research group synthesized 1,2,3-triazine 1-oxide derivatives that provide a direct synthetic route to the preparation of different heterocycles, including pyridines and isoxazoles. Therefore, in this study, a design strategy conjugation of the pyridine ring with fragments of natural products (Figure 1) was proposed to obtain new and more potent anti-trypanosomatid agents.

## 2. Materials and Methods

### 2.1. Chemistry

All reactions, until otherwise stated, were performed in oven-dried (150 °C) glassware with magnetic stirring under an air atmosphere. Thin-layer chromatography analysis (TLC) was carried out using EM Science silica gel 60 F254 plates; visualization was accomplished with UV light at 254 nm (UVP, INC, San Gabriel, CA, USA). Column chromatography was conducted on the Combi Flash^®^ Rf200 (Teledyne isco, Lincoln, NE, USA) purification system using normal-phase disposable columns. Nuclear magnetic resonance (NMR) spectra data were recorded on a Bruker spectrometer of 500 MHz and 300 MHz (Bruker, Billerica, MA, USA) and calibrated using the resonance signal of the residual non-deuterated solvent for ^1^H-NMR [δH = 7.26 ppm (CDCl_3_)] and deuterated solvent for ^13^C-NMR [δC = 77.16 (CDCl_3_)] as an internal reference at 298 K. Spectra were reported as follows: chemical shift (δ ppm), multiplicity (Mi), coupling constants (Hz), integration and assignment. The peak information was described as: br = broad, s = singlet, d = doublet, t = triplet, q = quartet, dd = doublet of doublet, m = multiplet, and comp = composite of magnetically non-equivalent protons. ^13^C-NMR spectra were collected on Bruker instruments of 126 MHz and 75 MHz (Bruker, Massachusetts, USA) with complete proton decoupling. High-resolution mass spectra (HRMS) were performed on a Bruker MicroTOFESI mass spectrometer (Bruker, MA, USA) with an ESI source using CsI or LTQ ESI positive ion calibration solution as the standard. Tetrahydrofuran, dichloromethane, and toluene were purified using a JC-Meyer solvent purification system (JC Meyer, Laguna Beach, CA, USA).

### 2.2. Synthesis

All ketones, ethyl diazoacetate, ethyl acetoacetate, 1,8-diazabicyclo [5.4.0]undec-7-ene (DBU) 1,4-diazabicyclo[2.2.2]octane (DABCO), and triethylamine (Et_3_N), were purchased from Sigma Aldrich (St. Louis, MO, USA), TCI America (Portland, OR, USA), and Alfa Aesar (Heysham, UK), and they were used without further purification. β-Keto-esters derived from cinnamyl alcohol, piperonyl alcohol, 1-adamantanemethanol, benzyl alcohol, menthol, citronellol, geraniol, *p*-methyl benzyl alcohol, and borneol were synthesized by the reported procedure [19]. 1,2,3-triazine 1-oxides [20,21] were prepared by following the literature-reported procedure.

Synthesis of pyridine-2,5-dicarboxylate derivatives was performed as previously reported by De Angelis et al. [22]. Triazine 1-oxide 1a-c (0.3 mmol) was added all at once to a 5 mL dichloromethane (DCM) solution containing β-keto-esters 2a–j (0.45 mmol; 1.5 equiv.), and DBU (0.6 mmol; 2 equiv.) was added dropwise. The reaction continued for 30 min at room temperature. Once the reaction was completed, the solvent was removed under reduced pressure, and the product was isolated by flash chromatography (20–50% ethyl acetate/hexane solution) to give pyridine compounds **3**–**12** in 56–99% yield (Scheme 1). All compounds were characterized by ^1^H-NMR, ^13^C-NMR, and HRMS spectroscopy (Table S1, Figures S1–S58). Additionally, compound **6a** (CCDC2365195) was crystallized, and the ORTEP diagram is reported in Figure S59.

### 2.3. Biological Evaluation

#### 2.3.1. In Vitro Trypanocidal Activity

Epimastigotes from the reference strain of *T. cruzi* (NINOA) and an autochthonous isolate of *T. cruzi* (A1) were used for in vitro evaluation. Both strains were maintained in liver infusion tryptose (LIT) medium supplemented with 10% FBS and 0.1% penicillin-streptomycin. They were preserved by transferring 1 × 10^6^ parasites/mL into a new culture medium every week. To determine the trypanocide activity of pyridine-2,5-dicarboxylates dissolved in dimethyl sulfoxide (DMSO) at 0.2%. *T. cruzi* epimastigotes at 1 × 10^6^ cells/well were cultured with the corresponding drug at different concentrations (0.78–100 µM) in 96-well microliter plates and incubated for 48 h at 28 °C. DMSO was included as a negative control, and Nifurtimox and Benznidazole were included as positive controls. The metabolic activity was determined by the Alamar Blue assay. Briefly, after the incubation period, 20 µL of 2.5 mM resazurin solution was added to each well and incubated for 3 h. Three independent experiments were performed in triplicate. The half-maximum inhibitory concentration (IC_50_) was determined using probit analysis [23].

#### 2.3.2. In Vitro Leishmanicidal Activity

Promastigotes of *L. mexicana* from two strains: reference strain *L. mexicana* (MNYC/BZ/62/M379) (M379) and autochthonous isolate of *L. mexicana* (MHOM/MX/2017/UABJO17FCQEPS) (FCQEPS) were used in in vitro evaluation. Briefly, parasites were cultured in RPMI 1640 medium supplemented with 10% fetal bovine serum (FBS), 100 U/mL penicillin, 100 µg/mL streptomycin, and glutamine (2 mM). To determine the leishmanicidal activity of pyridine-2,5-dicarboxylates, the parasites, in their logarithmic growth phase (5 × 10^5^ parasites/mL), were cultured in 96-well microliter plates with the corresponding pyridine-2,5-dicarboxylate compound at different concentrations (0.78–100 µM). Compounds were dissolved in DMSO in a final volume of 200 µL for 48 h at 26 °C. Parasites treated with DMSO (0.2%) were used as a negative control, while treatments with Glucantime and Amphotericin B, at the same concentrations, were used as positive controls. The metabolic activity of the cells was determined using the 3-(4,5-Dimethylthiazol-2-yl)-2,5-diphenyltetrazolium bromide (MTT) method. IC_50_ was determined using probit analysis. Three independent experiments were performed in triplicate, and the data were analyzed for statistical significance (*p* < 0.05) using Biostat 5.3.0 software [23,24].

#### 2.3.3. Cytotoxicity and Selectivity Index

A cytotoxicity assay was performed using the murine macrophage J774.2 cell line, which was recloned from the original ascites and solid tumor J774.1 (according to the manufacturer). The cells were cultured in RPMI medium supplemented with 10% SBF, 100 U µg/mL penicillin, 100 µg/mL streptomycin, and glutamine (2 mM), and 5% CO_2_ atmosphere. The medium was changed every 2–3 days. Macrophages were used at 1 × 10^6^ cells and were incubated with compounds at different concentrations (0.75–100 µM, respectively) at 37 °C for 48 h in a 5% CO_2_ atmosphere. DMSO was used as a negative control (0.2%). The metabolic activity of macrophages was determined by the MTT method. The percentage of cell viability was calculated, and the half-maximal cytotoxic concentration (CC_50_) was determined using probit analysis. Three independent assays were conducted in triplicate. The selectivity index (SI) was calculated for the promastigotes of *L. mexicana* and *T. cruzi*, for all strains (CC_50_/IC_50_) [23,24].

### 2.4. ADMET In Silico Properties

All pyridine carboxylate compounds were subjected to pharmacokinetic profile prediction of their Absorption, Distribution, Metabolism, and Excretion (ADME) using the SwissADME and cytotoxicity (ProTox 3.0) [25].

## 3. Results

### 3.1. Chemistry

**Scheme 1:**
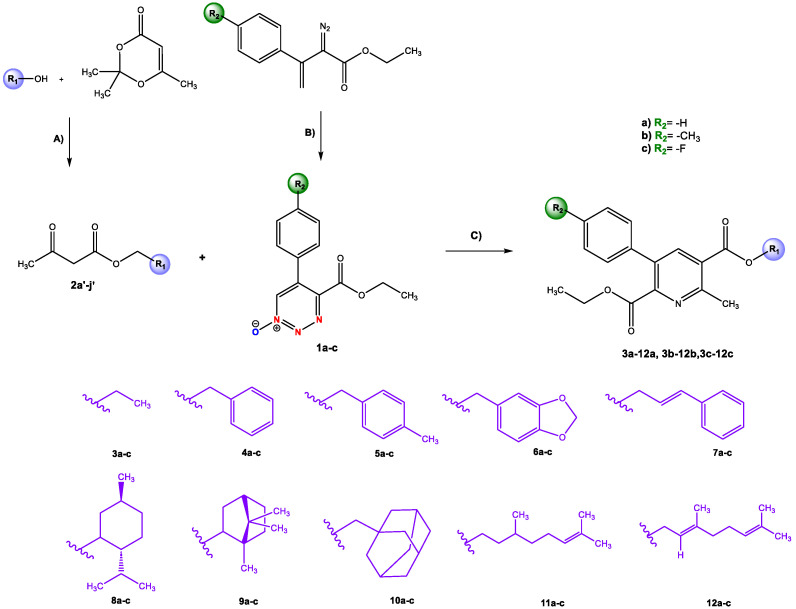
Synthetic route to obtain pyridine hybrids having natural product scaffolds. (**A**) Toluene, reflux, 12 h; (**B**) TBN (1.3 equiv.), DCM: HFIPA (20:1), rt, 30 min; (**C**) DBU (1.5 equiv.), DCM, rt, 30 min.

Pyridine-2,5-dicarboxylate esters 3a–c to 12a–c were synthesized as shown in Scheme 1 by reacting compounds 2a’–j’ with 1,2,3-triazine 1-oxide compounds **1a**–**c** in the presence of DBU at room temperature and DCM as solvent (Table 1). The intermediates 1a–c were obtained by a [5 + 1]-cycloaddition reaction between tert-butyl nitrite and three different vinyl diazo compounds (a-c) using a solvent mixture of DCM and HFIP at room temperature for 30 min. β-Keto-esters (2a’–j’) were synthesized by reacting 2,2,6-trimethyl-4H-1,3-dioxin-4-one with different alcohols bearing natural product scaffolds (a’–j’) under reflux for 12 h using toluene as the solvent. All compounds were characterized by ^1^H-NMR, ^13^C-NMR, and HRMS spectroscopy; some examples (**4b**, **8c**, **9b**, and **12b**) are described below. The characterization of the other compounds is provided in the supplementary material.

5-Benzyl 2-ethyl 6-methyl-3-(p-tolyl)pyridine-2,5-dicarboxylate, **4b**. Colorless oil, yield 63%. ^1^H-NMR (500 MHz, CDCl_3_) δ 8.3 (s, ^1^H), 7.5 (d, *J* = 6.6 Hz, 2H), 7.4 (comp, *J* = 13.1, 6.9 Hz, 3H), 7.3 (q, 4H), 5.4 (s, 2H), 4.2 (q, *J* = 7.2 Hz, 2H), 2.9 (s, 3H), 2.4 (s, 3H), 1.1 (t, *J* = 7.2 Hz, 3H). ^13^C-NMR (126 MHz, CDCl_3_) δ 166.9, 165.7, 158.3, 151.1, 140.4, 138.2, 135.4, 134.2, 133.9, 129.3, 128.7, 128.5 (*d*, *J* = 12.3 Hz), 128.2, 126.4, 67.4, 61.9, 24.6, 21.2, 13.8. HRMS (ESI) *m*/*z*: [M + H]^+^ Calculated for C_24_H_23_NO_4_ 390.1700; Found 390.1699.

2-Ethyl 5-((1R,2S,5S)-2-isopropyl-5-methylcyclohexyl) 3-(4-fluorophenyl)-6-methylpyridine-2,5-dicarboxylate, **8c**. Colorless oil, yield 73%. ^1^H-NMR (500 MHz, CDCl_3_) δ 8.2 (s, 1H), 7.4 (comp, 2H), 7.2 (t, *J* = 8.7 Hz, 2H), 5.0 (td, *J* = 11.0, 4.5 Hz, 1H), 4.2 (q, *J* = 7.2 Hz, 2H), 2.9 (s, 3H), 2.2–2.1 (m, 1H), 1.9 (pd, *J* = 7.0, 2.8 Hz, 1H), 1.8 (dt, *J* = 14.6, 3.1 Hz, 2H), 1.6–1.5 (m, 1H), 1.2 (dd, 3H), 1.1 (t, *J* = 7.2 Hz, 3H), 0.9 (*dd*, *J* = 17.0, 6.8 Hz, 8H), 0.8 (*d*, *J* = 7.0 Hz, 3H). ^13^C-NMR (126 MHz, CDCl_3_) δ 166.6, 165.5, 163.8, 161.9, 158.3, 150.6, 140.1, 133.4, 133.3, 132.9, 130.2, 130.1, 127.4, 115.7, 115.5, 75.9, 61.9, 47.1, 41.0, 34.2, 31.5, 26.5, 24.6, 23.5, 22.0, 20.8, 16.4, 13.8. HRMS (ESI) *m*/*z*: [M + H]^+^ Calculated for C_26_H_32_FNO_4_ 442.2388; Found 442.2379.

2-Ethyl 5-((1S,2R,4R)-1,7,7-trimethylbicyclo[2.2.1]heptan-2-yl) 6-methyl-3-(p-tolyl)pyridine-2,5-dicarboxylate, **9b**. Yellow oil, yield 99%. ^1^H-NMR (500 MHz, CDCl_3_) δ 8.2 (s, 1H), 7.3 (t, *J* = 6.6 Hz, 4H), 5.2 (dt, *J* = 9.9, 2.9 Hz, 1H), 4.2 (q, *J* = 7.2 Hz, 2H), 2.9 (s, 3H), 2.5 (*dd*t, *J* = 14.0, 9.9, 4.0 Hz, 1H), 2.4 (s, 3H), 2.0 (*ddd*, *J* = 13.4, 9.4, 4.4 Hz, 1H), 1.8 (*d*p, *J* = 11.9, 4.2 Hz, 1H), 1.8 (t, *J* = 4.6 Hz, 1H), 1.5–1.4 (m, 1H), 1.3 (comp, 2H), 1.1 (t, *J* = 7.2 Hz, 3H), 1.0 (s, 3H), 0.9 (*d*, *J* = 3.4 Hz, 6H). ^13^C-NMR (126 MHz, CDCl_3_) δ 166.9, 166.5, 157.7, 150.8, 140.2, 138.2, 134.3, 133.8, 129.4, 128.2, 127.3, 81.9, 61.8, 49.0, 48.0, 44.9, 37.0, 28.1, 27.5, 24.6, 21.2, 19.7, 18.9, 13.8, 13.7. HRMS (ESI) *m*/*z*: [M + H]^+^ Calculated for C_27_H_33_NO_4_ 436.2482; Found 436.2480.

(E)-5-(3,7-Dimethylocta-2,6-dien-1-yl) 2-ethyl 6-methyl-3-(p-tolyl)pyridine-2,5-dicarboxylate, **12b**. Colorless oil, yield 77%. ^1^H-NMR (500 MHz, CDCl_3_) δ 8.2 (s, 1H), 7.3 (*d*, *J* = 1.5 Hz, 4H), 5.5 (td, *J* = 7.3, 1.4 Hz, 1H), 5.1–5.1 (m, 1H), 4.9 (*d*, *J* = 7.2 Hz, 2H), 4.2 (q, *J* = 7.1 Hz, 2H), 2.9 (s, 3H), 2.4 (s, 3H), 2.1 (*d*, *J* = 5.9 Hz, 4H), 1.8 (s, 3H), 1.7 (s, 3H), 1.6 (s, 3H), 1.1 (t, *J* = 7.1 Hz, 3H). 13C-NMR (75 MHz, CDCl_3_) δ 166.9, 166.0, 158.1, 150.8, 143.2, 140.4, 138.1, 134.3, 133.9, 131.9, 129.2, 128.2, 126.8, 123.6, 117.8, 62.5, 61.8, 39.5, 26.2, 25.7, 24.4, 21.2, 17.7, 16.6, 13.8. HRMS (ESI) *m*/*z*: [M + H]^+^ Calculated for C_27_H_33_NO_4_ *m*/*z*: 436.2482; Found 436.2485.

### 3.2. Biological Evaluation

#### 3.2.1. Biological Evaluation Against *T. cruzi* and *L. Mexicana*

Initially, ten pyridine-2,5-dicarboxylate-esters derivatives (Series a) with a phenyl group at the C3-position and an ester group with fragments of natural products at the C5-position on the pyridine ring were obtained. To analyze the influence of electron-donating and electron-withdrawing groups (EDG and EWG), the *para*-methylphenyl (series b) and *para*-fluorophenyl (series c) groups at the C3-position on the pyridine ring were incorporated. These three series were evaluated against two *T. cruzi* (epimastigotes) and two *L. mexicana* (promastigotes) strains to determine their antiparasitic activity by calculating their IC_50_ (Table 2). The antiparasitic activity was classified as highly active (IC_50_ < 40 µM), moderately active (IC_50_ 41 to 100 µM), slightly active (IC_50_ > 100 µM), and inactive (IC_50_ > 200 µM).

These results reveal that the tested compounds had antiparasitic activity against *T. cruzi* epimastigotes and *L. mexicana* promastigotes. Moreover, pyridine compounds from series a, b, and c had superior activity over *T. cruzi* rather than *L. mexicana*. Although several pyridine compounds had superior IC_50_ values compared to the reference drug Benznidazole (Bzn), only compound **9b** demonstrated to be effective against *T. cruzi* NINOA and A1 strains with an IC_50_ < 40 µM. On the other hand, despite several compounds having antiparasitic activity, only compounds **8a**, **9b**, and **12b** were active against both *L. mexicana* M379 and FCQEPS strains with superior IC_50_ to Glucantime (IC_50_ = 134 µM). Accordingly, terpenoids such as menthyl, borneyl, and geranyl enhanced the trypanocidal and leishmanicidal activities exhibited by pyridine compounds.

#### 3.2.2. Cytotoxicity and SI Index Against Macrophage J774.2 Cell Line

In this study, almost all the pyridine-2,5-dicarboxylate esters of series a had very low cytotoxicity (CC_50_ > 200 µM) against the mouse macrophage J774.2 cell line, except compounds with citronellyl (**11a**) and geranyl (**12a**) esters at the C5 position on the pyridine moiety (CC_50_ of 118.7 and 25 µM, respectively). From series b, almost all compounds had low cytotoxicity (CC_50_ > 200 µM), except compound **11b** (CC_50_ = 169 µM) with the citronellyl group. Finally, the cytotoxicity of all compounds from series c was low (CC_50_ > 200 µM), suggesting that the presence of the fluorine atom (EWG) reduces the cytotoxic effect (Table 3).

SI was calculated to assess the relative selectivity of pyridine-2,5-dicarboxylate compounds, including reference drugs, over parasites. Seven compounds (**4a**, **6a**, **7a**, **4b**, **9b**, **10b**, and **9c**) had the highest SI values (SI > 4.71) against the *T. cruzi* NINOA strain, surpassing the standard drug Benznidazole (SI = 4.41). It is important to note that SI values for **9b** and **10b** were above 8.95.

### 3.3. ADMET In Silico Properties

The in silico analysis showed that pyridine-2,5-dicarboxylate esters had molecular weights ranging from 313.35 to 451.53 Da, consistently containing 5–8 hydrogen bond acceptors and lacking hydrogen bond donors across the series a, b, and c (Table 4). These derivatives demonstrated moderate lipophilicity (LogP 3.37–6.07), low topological polar surface areas (65.49–83.95 Å^2^), and a high count of rotatable bonds (7–12). Based on solubility coefficient predictions, most compounds were classified as having moderate to poor solubility, especially those with terpenoid esters, and with high gastrointestinal absorption (Table 4). Notably, compounds **3a**–**5a**, **3b**–**5b**, and **3c**–**4c** were predicted to cross the blood–brain barrier. This permeability seemed to decrease as lipophilicity (4.88) and molecular weight increased (>407.43 Da). In comparison, Nifurtimox, Benznidazole, and Glucantime (control drugs) exhibited low lipophilicity, high solubility, high gastrointestinal absorption (except Nifurtimox), and lacked blood–brain permeability. Additionally, some of these derivatives inhibit different isoforms of cytochrome P450 (Table 5). Toxicity predictions, such as hepatotoxicity, carcinogenicity, mutagenicity, and cytotoxicity, were inactive for all derivatives, except carcinogenicity for compounds **11a**–**b** and **12a**–**b**, which are active (Table 6).

## 4. Discussion

### 4.1. Chemical Synthesis of Pyridine-2,5-dicarboxylates

A novel series of pyridine-2,5-dicarboxylate-esters was successfully synthesized in moderate to high yields in the range of 56 to 99% (Table 1) using the inverse electron demand Diels–Alder (IEDDA) reaction with different β-keto-esters as dienophiles and 1,2,3-triazine-1-oxides as dienes (Scheme 1) [19]. The characterization of the compounds shown the presence of a singlet (S) in the range of 8.18–8.32 ppm in ^1^H-NMR spectra corresponds to the hydrogen atom on the pyridine ring; a quadruplet (q) signal at 4.24 ppm to two hydrogens of –CH_2_ and triplet (t) signal at 1.13 ppm to three hydrogens of –CH_3_ from ester group at the 2-position; and the presence of a singlet in the range 2.90–2.96 ppm corresponds to three protons of –CH_3_ group at the 6-position on the pyridine ring. ^13^C-NMR spectra from all pyridine-2,5-dicarboxylates fit with the number of carbons for each molecule. High-resolution mass spectrometry analysis was performed for each compound, which agreed with the mass of the proposed structures. Additionally, the structure of compound **6a** was confirmed by X-ray crystallography studies (See Supporting Information).

### 4.2. Biological Evaluation Against T. cruzi and L. mexicana

#### 4.2.1. SAR Analysis Against *T. cruzi* Epimastigotes NINOA Strain

The study encompassed all pyridine derivatives of series a, which contained a phenyl group at the C3 position, with **3a** serving as the initial reference. The results are shown in Table 2. The compound **3a** with an ethyl group at the C5-position had moderate activity against *T. cruzi* NINOA strain. Compounds **4a** and **5a**, which featured benzyl and *para*-methylbenzyl ester at the C5-position on the pyridine moiety, were also moderately active (IC_50_ = 42.44 and 56.58 µM, respectively).

The presence of piperonyl and cinnamyl esters at the C5-position on the pyridine ring in compounds **6a** and **7b**, respectively, improved trypanocidal activity by two-fold against the NINOA strain (IC_50_ < 28 µM). Moreover, **6a** and **7a** had better trypanocidal activity than the reference drug Benznidazole (IC_50_ = 30.3 µM).

Pyridine derivatives **8a** and **9a** with cyclic terpenoid ester systems bearing menthyl (monocyclic) and borneyl (bicyclic) groups, respectively, induced dissimilar trypanocidal activities. Compound **8a**, which is more sterically compressed, is inactive, while **9a** is moderately active against the NINOA strain. In contrast, **10a** with adamantanyl ester (tricyclic ring system) at the C5-position on the pyridine moiety was slightly active against the NINOA strain (IC_50_ = 105.43 µM). Saturation in the terpenoid esters of citronellyl and geranyl within compounds **11a** and **12a** produces different biological behavior. It is emphasized that compound **11a**, an unsaturated terpenoid with citronellyl ester, was inactive against the NINOA strain, whereas **12a** of the saturated terpenoid ester had slight trypanocidal activity.

Series b of pyridine compounds was designed by introducing the *para*-methyl group on the phenyl ring at the C3-position of the pyridine moiety to examine the impact of an EDG on the trypanocidal activity of pyridine compounds. The introduction of EDG in **3b** did not improve its trypanocidal activity (IC_50_ = 59.34 µM), which was as active as its analog **3a**. However, the presence of the *para*-methylphenyl substituent in compounds **4b**, **9b**, and **10b** induced high trypanocidal activity against the NINOA strain (IC_50_ < 26.4 µM). This trypanocidal activity was superior to Benznidazole (IC_50_ = 30.3 µM). This behavior could be due to a steric effect caused by EDG. In compound **8b**, the *para*-methylphenyl group caused slight activity against the NINOA strain. In contrast to analogs of series a, EDG resulted in a decrease in the trypanocidal activity in compounds **6b** and **7b**. Additionally, the trypanocidal activity of compounds **5b** and **12b** was significantly diminished by the presence of a *para*-methyl phenyl group on their pyridine moiety (IC_50_ > 200 µM). Pyridine **11b** remained inactive against the NINOA strain.

The *para*-fluorophenyl group, an EGW, was incorporated into series c of pyridine derivatives to investigate its influence on the antiparasitic activity against *T. cruzi* NINOA strain. The presence of the *para*-fluorophenyl group at the C3-position on the pyridine ring in compounds **4c**, **5c**, and **6c** led to the loss of trypanocidal activity (IC_50_ > 200 µM). Additionally, the trypanocidal activity of pyridine derivatives **3c** and **7c** was diminished by the effect of EWG. Observably, the trypanocidal activity of compounds **8c**, **9c**, **10c**, **11c**, and **12c** was enhanced by the inductive effect of the *para*-fluorophenyl group on the pyridine moiety in comparison to its analogs in series a. Particularly, pyridine **9c** demonstrated to be highly active (IC_50_ = 26.2 µM), even more active than Benznidazole.

#### 4.2.2. SAR Analysis Against *T. cruzi* Epimastigotes A1 Strain

From series a, compounds **3a**, **4a**, **5a**, and **9a** with ethyl, benzyl, 4-methylbenzyl, and borneyl esters at the C5-position on the pyridine moiety had better trypanocidal activity (IC_50_ < 29 µM) than Benznidazole (IC_50_ < 39 µM) against *T. cruzi* A1 strain. Remarkably, **5a** with the incorporation of the *para*-methyl group on the benzyl ring slightly increased the activity (IC_50_ = 21.31 µM), similar to Nifurtimox (IC_50_ = 19.3 µM). The presence of *para*-methylbenzyl and borneyl esters at the C5 position on the pyridine moiety in **5a** and **9a** could induce a steric effect for both compounds that enhanced the trypanocidal activity. Compound **10a** with adamantanyl ester was moderately active against the A1 strain (IC_50_ = 46.89 µM). The geranyl ester was incorporated at the C5 position on the pyridine scaffold, causing slight activity of **12a** against the A1 strain. In contrast to citronellyl, the presence of an additional double bond on geranyl ester may decrease the flexibility of the ester group, resulting in improved trypanocidal activity. Compounds **6a**, **7a**, **8a**, and **11a** were not active against *T. cruzi* A1 strain (IC_50_ > 200 µM).

In series b, the addition of an EDG reduces two-fold the trypanocidal activity in compounds **3b**, **4b**, **9b**, and **10b** against the A1 strain. Additionally, the EDG at the C3 position on the pyridine moiety led **5b**, **7b**, and **12b** to completely lose their trypanocidal activity. Notably, the *para*-methylphenyl group at the C3-position in 5b resulted in a biologically inactive compound when compared to **5a**. However, compound 9b had trypanocidal activity (IC_50_ = 38.49 µM) against A1, comparable to Benznidazole. Therefore, the EDG reduced, and in some cases, abolished trypanocidal activity, except in compounds **6b**, **8b**, and **11b**, where the EDG improved the trypanocidal activity against the A1 strain.

The trypanocidal activity of compound **3c** in series c was not comparable to that of **3a**, but it was superior to that of **3b**. This means that the presence of EWG (fluorine) at the *para*-position on the phenyl ring improves the biological properties. Furthermore, compound **5c** exhibited comparable trypanocidal activity to **5a** (IC_50_ = 20.8 µM) and Nifurtimox (IC_50_ = 19.30 µM).

Surprisingly, compounds **6c**, **7c**, **8c**, **10c**, **11c**, and **12c** exhibited superior antiparasitic activity to their analogs of series a and b and were superior to Benznidazole. This suggests that the next order in the trypanocidal activity is –F > –CH_3_ > –H. Specifically, compounds **5c**, **7c**, and **8c** had trypanocidal activity similar to Nifurtimox (IC_50_ = 19.30 µM) compared to their analogs of series a and b. In summary, all compounds bearing *para*-fluorophenyl instead of *para*-methyl phenyl group at the C3-position had an increase in their trypanocidal activity, except for **4c** and **9c** (IC_50_ > 200 µM).

#### 4.2.3. Biological Activity Against *L. mexicana*

The leishmanicidal activity (IC_50_) of the pyridine-2,5-dicarboxylate esters series a, b, and c against two *L. mexicana* strains, M379 and FCQEPS, was also evaluated. The IC_50_ values of pyridine compounds against *L. mexicana* M379 strain were from 54.79 to 85.89 µM, and against FCQEPS from 57.03 to 161.53 µM. The leishmanicidal activity of compound **9b** was the most effective, with an IC_50_ of less than 40 µM. Moreover, compounds **12b** and **10c** also had significant leishmanicidal activity (IC_50_ values < 56 µM). Except for compound **10a**, all pyridine-2,5-dicarboxylate esters exhibited leishmanicidal activity superior to the reference drug Glucantime (IC_50_ > 125 µM).

#### 4.2.4. SAR Analysis Against *L. mexicana* M379 Strain

From series a, only compounds 8a with a menthyl ester at the C5 position on the pyridine ring (IC_50_ = 79.09 µM) and **10a** with an adamantanyl ester at the C5 position (IC_50_ = 82.19 µM) had moderate leishmanicidal activity against the M379 strain. The biological effect of both compounds was superior to that of the reference drug Glucantime (IC_50_ = 133.96 µM). Compounds **3a**, **4a**, **5a**, **6a**, **7a**, **9a**, **11a**, and **12a** did not have antiparasitic activity.

In compounds of series b, the addition of the *para*-methyl group on the phenyl ring at C3-position in compounds **8b** and **10b** caused the loss of leishmanicidal activity (IC_50_ > 200 µM) in comparison to their analogs of series a. Conversely, compounds **9b** with borneyl and **12b** with geranyl ester groups had better leishmanicidal activity against M379 strain (IC_50_ = 39.95 and 55.17 µM, respectively) than glucantime. This increase in activity may be due to the steric effect of esters and *para*-methylphenyl groups. However, compounds **3b**, **4b**, **5b**, **6b**, **7b**, and **11b** did not have antiparasitic activity.

Furthermore, the presence of the *para*-fluorophenyl group at the C3-position in compounds of series c enhanced the leishmanial activity of pyridine **10c** with adamantanyl ester (IC_50_ = 54.79 µM) compared to **10a** and **10b**. The same biological effect is noted for **12c** (IC_50_ = 85.98 µM) compared to **12a**. Nevertheless, the same structural change decreased the activity of **12c** compared to its analog **12b**. In both cases, the leishmanial activity of **12b** and **12c** is better than Glucantime. Like their analogs in series a, **3c**, **4c**, **5c**, **6c**, **7c**, **9c**, and **11c** were inactive.

#### 4.2.5. SAR Analysis Against *L. mexicana* FCQEPS Strain

Compounds **3a**, **4a**, **6a**, **7a**, and **9a**, with ethyl, benzyl, piperonyl, cinnamyl, and borneyl ester groups at the C5 position on the pyridine moiety, did not have leishmanicidal activity against FCQEPS strain. However, the addition of the *para*-methyl group on the benzyl ester caused compound **5a** to have moderate leishmanicidal activity (IC_50_ = 94.95 µM). This biological response could be attributed to the steric effect of the *para*-methyl group on the benzyl ring. Moreover, compounds **8a** and **10a** bearing monocyclic and tricyclic alkane esters had different leishmanicidal effects, **8a** being the most active one (IC_50_ = 82.72). Compounds **11a** with citronellyl (IC_50_ = 73.04 µM) and **12a** with geranyl (IC_50_ = 75.34 µM) esters had similar leishmanicidal activity. In summary, compounds **5a**, **8a**, **11a**, and **12a** had better leishmanicidal activity than Glucantime (IC_50_ = 125.23 µM).

In series b, compounds **3b**, **4b**, and **6b** did not exhibit an increase in leishmanicidal activity when the *para*-methyl group (EDG) was incorporated on the phenyl ring at the C3 position on the pyridine moiety (IC_50_ > 200 µM). In the same vein, the leishmanicidal activity of compounds **5b**, **8b**, and **11b** (IC_50_ > 200 µM) was abrogated by the incorporation of EDG in comparison to the analogs of series a. Nevertheless, the leishmanicidal activity of compounds **7b** (IC_50_ = 116.71 µM) and **9b** (IC_50_ = 79.87 µM) was enhanced in comparison to **7a** and **9a** by the incorporation of EDG at the C3 position on the pyridine moiety. The EDG enhanced the leishmanicidal activity of compounds **10b** (IC_50_ = 92.93 µM) and **12b** (IC_50_ = 57.03 µM) in comparison to analogs of series a, in accordance with this biological tendency. In summary, the leishmanicidal activity of compounds **7b**, **9b**, **10b**, and **12b** was better than that of Glucantime (IC_50_ = 125.23 µM) against the FCQEPS strain.

In series c, the addition of an EWG in compound **3c** improves the leishmanicidal activity (IC_50_ = 118.48 µM) compared to **3a** and **3b**. Similarly, compound 8c had higher leishmanicidal activity (IC_50_ = 90.85 µM) compared to **8b**. However, the EWG caused the reduction in leishmanicidal activity of 12c (IC_50_ = 113.75 µM) compared to **12a** and **12b** analogs. The EWG did not improve the leishmanicidal activity of compounds **4c**, **6c**, **7c**, and **9c** (IC_50_ > 200 µM) compared to analogs of series a. While for compounds **5c**, **10c**, and **11c**, the presence of an EWG abolished their leishmanicidal activity when compared to their analogs of series a. In summary, compounds **3c**, **8c**, and **12c** have leishmanicidal activity superior to Glucantime (IC_50_ = 125.23 µM) despite the introduction of the EWG against FCQEPS.

#### 4.2.6. Cytotoxicity and Selectivity Index

The A1 strain of *T. cruzi* exhibited SI values (>3.8) that were superior to Benznidazole (SI = 3.42) in sixteen compounds. Surprisingly, the SI values of compounds **5a**, **5c**, **7c**, and **8c** surpassed the Nifurtimox SI (8.50). The results of this indicate that the pyridine derivatives **5a**, **9b**, **10b**, **5c**, **7c**, and **8c** are promising structures that have the potential to provide more effective therapeutic options while reducing the risk of adverse effects. These structures may offer better selectivity against *T. cruzi* epimastigotes.

The SI values of compounds **8a**, **10a**, **9b**, **12b**, **10c**, and **12c** were better than the standard drug Glucantime (SI = 2.03) against *L. mexicana* M379 strain. Therefore, these compounds are suitable candidates for further structure optimization to result in better selectivity. Cytotoxic studies against *L. mexicana* FCQEPS strain showed that only compounds **8a**, **9b**, **12b**, and **8c** had better SI than Glucantime (SI = 2.18).

Although some pyridine derivatives had SI values superior to the reference drugs, careful modification may be performed to reach the ideal SI value (SI = 10). However, the SI values of compounds **5a**, **9b**, **10b**, **5c**, **7c**, and **8c** approach the ideal SI value (>10) against tested parasites.

### 4.3. ADMET In Silico Properties

Prediction of absorption, distribution, metabolism, and excretion (ADME) parameters by computational methods aids in the selection of drug candidates that are more promising to have an ideal behavior in the body. Additionally, Lipinski’s Rule of 5 (molecular weight ≤ 500, hydrogen bond acceptors ≤ 10, hydrogen bond donors ≤ 5, and logP ≤ 5) and Veber’s Rule (rotatable bonds ≤ 10 and polar surface area ≤ 140 Å^2^) are the metrics for the selection of compounds more likely to become orally available [26,27,28]. The ADME profile of all synthesized pyridine derivatives (Table 4) was calculated using the SwissADME web server [25].

In summary, all pyridine compounds satisfy the criteria for MW, HBA, and HBD for Lipinski’s Rule. Compounds **8a**, **10a**, **11a**, and **12a** had a logP above 5. The same tendency is noted for their analogs of series b and c. Moreover, the presence of EDG and EWG in compounds **7b** and **7c**, respectively, led to an increase in their logP value (logP = 5.8). According to the results obtained, compounds are considered to comply with the Lipinski rule when they meet 3 of the 4 criteria established for this rule, as is shown in Table 4.

All pyridine derivatives comply with Veber’s Rule criteria for TPSA and rotatable bonds, except for compounds containing citronellyl and geranyl ester groups (**11a**, **12a**, **11b**, **12b**, **11c**, and **12c**) with RB > 10. These results imply that pyridine derivatives are highly bioavailable orally. In addition, compounds with ethyl, benzyl, *para*-methylbenzyl, piperonyl, and cinnamyl esters are more soluble than those with methyl, borneyl, adamantanyl, citronellyl, and geranyl ester groups (Table 4).

In Table 5, pyridine derivatives also showed high gastrointestinal absorption and low blood–brain barrier permeability. Only compounds of series **3a**, **4a**, **5a**, and **6a** from series a are BBB permeant. However, the addition of EDG caused **3b**, **4b**, and **5b** to be BBB permeants, while **3c** and **4c** with EWG were BBB permeants. The analysis of the data suggested that most of these compounds exhibit favorable drug-likeness properties. According to the results, the compounds with the best in vitro activity (**9b**, **10b**, **5c**, **7c**, **8c**, **11c**, **12b**) showed good absorption but should be evaluated with caution due to CYP inhibition. Lipophilic terpene derivatives (geranyl, citronellyl, bornyl) show relative potency against *L. mexicana* but limited penetration of the BBB, making them more suitable for cutaneous leishmaniasis than for Chagas disease with systemic involvement.

The toxicity predictions (hepatotoxicity, mutagenicity, carcinogenicity, and cytotoxicity) were negative, except for the compounds **11a**, **11b** (Citronellyl-), **12a**, and **12b** (Geranyl-), which are potentially carcinogenic (Table 6).

In this context, integrating ADMET predictions with computational studies, such as molecular docking, will be key to obtaining information about the potential pharmacological target of these pyridine derivatives. Previous research has postulated that the mode of action of pyridine derivatives against trypanosomatid parasites could be related to the enzymes such as trypanothione reductase, NADH-fumarate reductase [29], or the inhibition of enzymes belonging to the P450 system, mainly CYP51, as in the case of [1,2,3]triazolo[1,5-a]pyridyl ketones and pyridyl ketone derivatives [30]. Therefore, it is suggested that in future research, the mode of action could be confirmed through in silico and enzymatic studies.

## 5. Conclusions

Thirty pyridine-2,5-dicarboxylate-based esters, grouped in series a, b, and c, were synthesized by inverse electron demand Diels–Alder (IEDDA) reaction using ten different β-keto-esters and three 1,2,3-triazine-1-oxides as new antiprotozoal agents. Compounds **6a**, **7a**, **4b**, **9b**, **10b**, and **9c** stood out for anti-*T. cruzi* activity against the epimastigote form of the NINOA strain. These compounds also had low cytotoxicity (CC_50_ > 200 µM) and an SI superior to Benznidazole (SI = 4.41), which makes them promising drug candidates. In addition, compounds **3a**, **4a**, **5a**, **9a**, **9b**, **5c**, **6c**, **7c**, **8c**, **10c**, and **11c** were also noted for having superior antiparasitic activity than Benznidazole against strain A1. Additionally, compounds **5a**, **5c**, **7c**, and **8c** are similar in activity to Nifurtimox. These compounds are also not cytotoxic and show higher SI values superior to Benznidazole (SI = 3.4) and Nifurtimox (SI = 8.5). Compounds **3a**, **4a**, **5a**, **4b**, and **8c** showed the best activity for both strains of *T. cruzi* with IC_50_ ≤ 56.58 µM.

On the other hand, compounds **8a**, **10a**, **9b**, **12b**, **10c**, and **12c** had better leishmanicidal activity than Glucantime against the M379 strain. Additionally, compounds **5a**, **8a**, **11a**, **12a**, **7b**, **9b**, **10b**, **12b**, **3c**, **8c**, and **12c** were highlighted for their leishmanicidal activity against the FCQEPS strain. In the same fashion, these compounds were non-cytotoxic and had SI values superior to Glucantime (SI = 2). Of the three series of pyridine derivatives, compound **9b** stands out due to its potent antiparasitic activity, low cytotoxicity, and high selectivity against both parasites. Compounds **8b**, **10a**, **9b**, and **12b** have better activity (IC_50_ ≤ 161.53 µM) in both strain of *L. Mexicana*.

Finally, the computational prediction of ADMET properties indicates that most of the pyridine-based derivatives possess favorable bioavailability characteristics and promising drug-likeness profiles. The results obtained in the study indicate that pyridine-2,5-dicarboxylate derivatives are easy to obtain by chemical synthesis, which offer novel structures and are compounds with potent and selective antiparasitic activity. Future work will be carried out to lead optimization and gain insight into possible modes of action of these pyridine-based compounds.

## Figures and Tables

**Table 1:**
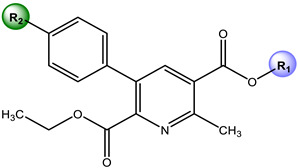
Chemical structures and yields of the isolated compounds **3a**–**12a**, **3b**–**12b**, and **3c**–**12c**.

Compound	R_1_	R_2_					
		Series a	Yield %	Series b	Yield %	Series c	Yield %
**3**	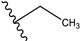	–H	56	–CH_3_	95	–F	68
**4**	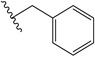	–H	72	–CH_3_	63	–F	71
**5**	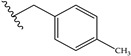	–H	74	–CH_3_	86	–F	68
**6**	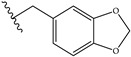	–H	71	–CH_3_	71	–F	75
**7**	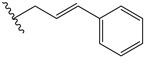	–H	66	–CH_3_	77	–F	75
**8**	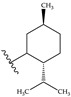	–H	80	–CH_3_	75	–F	73
**9**	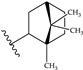	–H	75	–CH_3_	99	–F	81
**10**	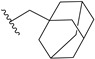	–H	75	–CH_3_	72	–F	95
**11**	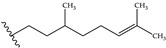	–H	85	–CH_3_	67	–F	70
**12**	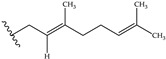	–H	89	–CH_3_	77	–F	79

**Table 2:** Trypanocidal (IC_50_ in µM against epimastigotes) and leishmanicidal (IC_50_ in µM against promastigotes) activity of pyridine-2,5-dicarboxylate compounds.

Compound	R_1_	R_2_	*T. cruzi* IC_50_ (µM ± SD)		*L. mexicana* IC_50_ (µM ± SD)	
			NINOA	A1	M379	FCQEPS
**3a**	Et-	–H	54.48 ± 2.36	26.65 ± 3.75 *	>200	>200
**4a**	Benzyl-	–H	42.44 ± 1.88 *	28.53 ± 1.50 *	>200	>200
**5a**	4-methylbenzyl-	–H	56.58 ± 1.26	21.31 ± 1.8 *	>200	94.95 ± 1.36 *
**6a**	Piperonyl-	–H	27.15 ± 0.84 *	>200	>200	>200
**7a**	Cinnamyl-	–H	27.77 ± 1.66 *	>200	>200	>200
**8b**	(–)-Menthyl-	–H	>200	>200	79.09 ± 0.01 *	82.72 ± 0.03 *
**9a**	(–)-Borneyl-	–H	55.75 ± 2.50	25.03 ± 2.98 *	>200	>200
**10a**	Adamantanyl-	–H	105.44 ± 1.34	46.90. ± 1.96	82.19 ± 0.01 *	161.53 ± 0.03
**11a**	Citronellyl-	–H	>200	>200	>200	73.04 ± 1.78 *
**12a**	Geranyl-	–H	106.30 ± 1.43	117.80 ± 0.94	>200	75.34 ± 4.28 *
**3b**	Et-	–Me	59.40 ± 1.45	106.40 ± 1.163	>200	>200
**4b**	Benzyl-	–Me	26.40 ± 1.66 *	52.55 ± 1.87	>200	>200
**5b**	4-methylbenzyl–	–Me	>200	>200	>200	>200
**6b**	Piperonyl-	–Me	53.96 ± 2.29	50.18 ± 2.31	>200	>200
**7b**	Cinnamyl-	–Me	99.23 ± 0.76	>200	>200	116.71 ± 0.03
**8b**	(–)-Menthyl-	–Me	106.85 ± 0.50	51.64 ± 1.60	>200	>200
**9b**	(–)-Borneyl-	–Me	22.29 ± 1.60 *	38.50 ± 2.20	39.96 ± 0.01 *	79.87 ± 0.02 *
**10b**	Adamantanyl-	–Me	20.98 ± 0.15 *	111.38 ± 1.14	>200	92.93 ± 0.02 *
**11b**	Citronellyl-	–Me	>200	51.61 ± 0.59	>200	>200
**12b**	Geranyl-	–Me	>200	>200	55.18 ± 2.75 *	57.03 ± 0.02 *
**3c**	Et-	–F	100.00 ± 1.86	91.73 ± 1.72	>200	118.48 ± 2.08
**4c**	Benzyl-	–F	>200	>200	>200	>200
**5c**	4-methylbenzyl-	–F	>200	20.80 ± 0.10 *	>200	>200
**6c**	Piperonyl-	–F	>200	25.43 ± 0.60 *	>200	>200
**7c**	Cinnamyl-	–F	63.09 ± 1.64	22.79 ± 0.69 *	>200	>200
**8c**	(–)-Menthyl-	–F	46.02 ± 3.33	21.64 ± 1.51 *	>200	90.85 ± 5.0 *
**9c**	(–)-Borneyl-	–F	26.23 ± 1.55 *	>200	>200	>200
**10c**	Adamantanyl-	–F	50.56 ± 1.06	38.23 ± 2.86	54.80 ± 0.02 *	>200
**11c**	Citronellyl-	–F	79.88 ± 2.60	24.88 ± 4.10 *	>200	>200
**12c**	Geranyl-	–F	49.10 ± 2.5	50.08 ± 1.0	85.98 ± 0.02 *	113.75 ± 7.03 *
Nfx			7.09 ± 0.12	19.30 ± 0.08		
Bzn			30.3 ± 0.03	39.08 ± 0.07		
Glc					133.96 ± 4.32	125.23 ± 11.64

Nfx = Nifurtimox; Bzn = Benznidazole; Glc = Glucantime. M379:MNYC/BZ/62/M379. FCQEPS:MHOM/MX/2017/UABJO17FCQEPS. IC_50_: Half-maximal inhibitory concentration. SD: standard deviation. The * indicates statistically significant differences with the drug reference (*p* < 0.05).

**Table 3:** Cytotoxicity (CC_50_ in µM) against macrophage J774.2 cell line and selectivity index of pyridine-2,5-dicarboxylate compounds.

Compound	Macrophage J774.2 CC_50_ (µM ± SD)	Selectivity Index			
		*T. cruzi*		*L. mexicana*	
		NINOA	A1	M379	FCQEPS
**3a**	>200	>3.6	>7.5	>1	>1
**4a**	>200	>4.7	>7.0	>1	>1
**5a**	>200	>3.5	>9.3	>1	>2.1
**6a**	>200	>7.3	>1	>1	>1
**7a**	>200	>7.20	>1	>1	>1
**8a**	>200	>1	>1	>2.5	>2.4
**9a**	>200	>3.6	>8.0	>1	>1
**10a**	>200	>1.9	>4.3	>2.4	>1.2
**11a**	118.7 ± 7.3	0.5	0.6	0.6	1.6
**12a**	25.0 ± 0.8	0.2	0.2	0.1	0.3
**3b**	>200	>3.3	>1.9	>1	>1
**4b**	>200	>7.5	>3.8	>1	>1
**5b**	>200	>1	>1	>1	>1
**6b**	>200	>3.7	>4.0	>1	>1
**7b**	>200	>2.0	>1	>1	>1.7
**8b**	>200	>1.8	>3.9	>1	>1
**9b**	>200	>8.9	>5.2	>5.0	>2.5
**10b**	>200	>9.5	>1.8	>1	>2.1
**11b**	169.8 ± 1.3	0.8	3.3	0.8	0.8
**12b**	>200	>1	>1	>3.6	>3.5
**3c**	>200	>1.9	>2.2	>1	>1.7
**4c**	>200	>1	>1	>1	>1
**5c**	>200	>1	>9.6	>1	>1
**6c**	>200	>1	>7.9	>1	>1
**7c**	>200	>3.1	>8.8	>1	>1
**8c**	>200	>4.3	>9.2	>1	>2.2
**9c**	>200	>7.6	>1	>1	>1
**10c**	>200	>4.0	>5.2	>3.6	>1
**11c**	>200	>2.5	>8.0	>1	>1
**12c**	>200	>4.1	>4.0	>2.3	>1.7
**Nfx**	164.2	23.2	8.5		
**Bzn**	133.9	4.4	3.4		
**Glc**	273.2			2.0	2.2

Nfx = Nifurtimox; Bnz = Benznidazole; Glc = Glucantime; CC_50_: Half-maximal cytotoxic concentration; Selectivity index:(CC_50_/IC_50_).

**Table 4:** Physicochemical properties of pyridine-2,5-dicarboxylate compounds by SwissADME.

Compound	Physicochemical Properties							
	MW (g/mol)	HBA	HBD	RB	TPSA Å^2^	Log P	RB	Log S
**3a**	313.35	5	0	7	65.49	3.37	7	Moderately soluble
**4a**	375.42	5	0	8	65.49	4.25	8	Moderately soluble
**5a**	389.44	5	0	8	65.49	4.57	8	Moderately soluble
**6a**	419.43	7	0	8	83.95	4.07	8	Moderately soluble
**7a**	401.45	5	0	9	65.49	4.77	9	Moderately soluble
**8a**	423.54	5	0	8	65.49	5.42	8	Poorly soluble
**9a**	421.53	5	0	7	65.49	4.39	7	Poorly soluble
**10a**	433.54	5	0	8	65.49	5.19	8	Poorly soluble
**11a**	423.54	5	0	12	65.49	5.73	12	Poorly soluble
**12a**	421.53	5	0	11	65.49	5.53	11	Poorly soluble
**3b**	327.37	5	0	7	65.49	3.73	7	Moderately soluble
**4b**	389.44	5	0	8	65.49	4.56	8	Moderately soluble
**5b**	403.47	5	0	8	65.49	4.93	8	Moderately soluble
**6b**	433.45	7	0	8	83.95	4.42	8	Moderately soluble
**7b**	415.48	5	0	9	65.49	5.08	9	Moderately soluble
**8b**	437.57	5	0	8	65.49	5.75	8	Poorly soluble
**9b**	435.56	5	0	7	65.49	4.67	7	Poorly soluble
**10b**	447.57	5	0	8	65.49	5.55	8	Poorly soluble
**11b**	437.57	5	0	12	65.49	6.07	12	Poorly soluble
**12b**	435.56	5	0	11	65.49	5.93	11	Poorly soluble
**3c**	331.34	6	0	7	65.49	3.69	7	Moderately soluble
**4c**	393.41	6	0	8	65.49	4.54	8	Moderately soluble
**5c**	407.43	6	0	8	65.49	4.88	8	Moderately soluble
**6c**	437.42	8	0	8	83.95	4.4	8	Moderately soluble
**7c**	419.44	6	0	9	65.49	5.08	9	Moderately soluble
**8c**	441.53	6	0	8	65.49	5.76	8	Poorly soluble
**9c**	439.52	6	0	7	65.49	4.68	7	Poorly soluble
**10c**	451.53	6	0	8	65.49	5.5	8	Poorly soluble
**11c**	441.53	6	0	12	65.49	6.04	12	Poorly soluble
**12c**	439.52	6	0	11	65.49	5.83	11	Poorly soluble
Nfx	287.29	6	0	3	117.08	0.54	3	Soluble
Bzn	260.25	4	1	6	92.74	0.49	6	Soluble
Glc	365.98	9	7	6	167.55	−2.9	6	Highly soluble

MW = Molecular Weight; HBA = Hydrogen Bond Acceptor; HBD = Hydrogen Bond Donor; RB = Rotatable Bond; TPSA = Topological Polar Surface Area; Log P = partition coefficient; Log S = solubility coefficient.

**Table 5:** Pharmacokinetic properties of pyridine-2,5-dicarboxylate compounds by SwissADME.

Compound	Pharmacokinetic Properties							
	GI Absorption	BBB Permeant	Pgp Substrate	CYP1A2 Inhibitor	CYP2C19 Inhibitor	CYP2C9 Inhibitor	CYP2D6 Inhibitor	CYP3A4 Inhibitor
**3a**	High	Yes	No	Yes	Yes	Yes	No	No
**4a**	High	Yes	No	Yes	Yes	Yes	No	Yes
**5a**	High	Yes	No	Yes	Yes	Yes	No	Yes
**6a**	High	No	No	Yes	Yes	Yes	No	Yes
**7a**	High	Yes	No	Yes	Yes	Yes	No	Yes
**8a**	High	No	No	No	Yes	Yes	Yes	Yes
**9a**	High	No	Yes	No	No	Yes	No	Yes
**10a**	High	No	Yes	No	Yes	Yes	Yes	Yes
**11a**	High	No	No	No	Yes	Yes	No	Yes
**12a**	High	No	No	No	No	Yes	No	Yes
**3b**	High	Yes	No	Yes	Yes	Yes	No	No
**4b**	High	Yes	No	Yes	Yes	Yes	No	Yes
**5b**	High	Yes	No	No	Yes	Yes	No	Yes
**6b**	High	No	No	No	Yes	Yes	No	Yes
**7b**	High	No	No	Yes	Yes	Yes	No	Yes
**8b**	High	No	Yes	No	Yes	Yes	Yes	Yes
**9b**	High	No	Yes	No	No	Yes	No	Yes
**10b**	High	No	Yes	No	Yes	Yes	Yes	Yes
**11b**	High	No	Yes	No	No	Yes	No	Yes
**12b**	High	No	No	No	No	Yes	No	Yes
**3c**	High	Yes	No	Yes	Yes	Yes	No	No
**4c**	High	Yes	No	Yes	Yes	Yes	No	Yes
**5c**	High	No	No	Yes	Yes	Yes	No	Yes
**6c**	High	No	No	Yes	Yes	Yes	No	Yes
**7c**	High	No	No	Yes	Yes	Yes	No	Yes
**8c**	High	No	No	No	Yes	Yes	Yes	Yes
**9c**	High	No	Yes	No	No	Yes	No	No
**10c**	High	No	Yes	No	Yes	Yes	Yes	Yes
**11c**	High	No	Yes	No	No	Yes	No	Yes
**12c**	High	No	No	No	No	Yes	No	Yes
Nfx	Low	No	No	No	No	No	No	No
Bzn	High	No	No	No	No	No	No	No
Glc	High	No	No	No	No	No	No	No

GI Absorption = Gastrointestinal Absorption; Pgp = P-glycoprotein; BBB Permeant = Blood–Brain Barrier Permeability.

**Table 6:** Toxicity predictions of pyridine-2,5-dicarboxylate compounds by ProTox 3.0.

Compound	Toxicity			
	Hepatoxicity	Carcinogenicity	Mutagenicity	Cytotoxicity
**3a**	Inactive	Inactive	Inactive	Inactive
**4a**	Inactive	Inactive	Inactive	Inactive
**5a**	Inactive	Inactive	Inactive	Inactive
**6a**	Inactive	Inactive	Inactive	Inactive
**7a**	Inactive	Inactive	Inactive	Inactive
**8a**	Inactive	Inactive	Inactive	Inactive
**9a**	Inactive	Inactive	Inactive	Inactive
**10a**	Inactive	Inactive	Inactive	Inactive
**11a**	Inactive	Active	Inactive	Inactive
**12a**	Inactive	Active	Inactive	Inactive
**3b**	Inactive	Inactive	Inactive	Inactive
**4b**	Inactive	Inactive	Inactive	Inactive
**5b**	Inactive	Inactive	Inactive	Inactive
**6b**	Inactive	Inactive	Inactive	Inactive
**7b**	Inactive	Inactive	Inactive	Inactive
**8b**	Inactive	Inactive	Inactive	Inactive
**9b**	Inactive	Inactive	Inactive	Inactive
**10b**	Inactive	Inactive	Inactive	Inactive
**11b**	Inactive	Active	Inactive	Inactive
**12b**	Inactive	Active	Inactive	Inactive
**3c**	Inactive	Inactive	Inactive	Inactive
**4c**	Inactive	Inactive	Inactive	Inactive
**5c**	Inactive	Inactive	Inactive	Inactive
**6c**	Inactive	Inactive	Inactive	Inactive
**7c**	Inactive	Inactive	Inactive	Inactive
**8c**	Inactive	Inactive	Inactive	Inactive
**9c**	Inactive	Inactive	Inactive	Inactive
**10c**	Inactive	Inactive	Inactive	Inactive
**11c**	Inactive	Inactive	Inactive	Inactive
**12c**	Inactive	Inactive	Inactive	Inactive

## Data Availability

The datasets used and/or analyzed during the current study are available from the corresponding author on reasonable request.

## Supplementary Materials

The following supporting information can be downloaded at: https://www.mdpi.com/article/10.3390/pharmaceutics17101271/s1, Table S1: Analytical and spectral characterization of the pyridine compounds (**3a**–**c**–**12a**–**c**), Figures S1–S60: NMR spectra; Figure S61: ORTEP drawing of the molecular structure of 6a.

## Abbreviations

*T. cruzi*	*Trypanosoma cruzi*
*L. mexicana*	*Leishmania mexicana*
TLC	Thin Layer Chromatography
IC_50_	half-maximum inhibitory concentration
MTT	3-(4,5-Dimethylthiazol-2-yl)-2,5-diphenyltetrazolium bromide
CC_50_	Half-maximal cytotoxic concentration
SI	Selectivity index
ESI	Electrospray ionization
ADMET	Administration Distribution Metabolism Excretion Toxicity
EWG	Electron-withdrawing Group
EDG	electron-donating group
DMSO	Dimethyl sulfoxide
FBS	Fetal Bovine Serum
CO_2_	Carbon dioxide
