# Supplementary material for: Design, Synthesis, In Vitro and In Silico Biological Evaluation of New Pyridine-2,5-Dicarboxylates Esters Bearing Natural Source Fragments as Anti-Trypanosomatid Agents
Source: Pharmaceutics. 2025 Sep 28;17(10):1271. doi: 10.3390/pharmaceutics17101271 (PMC12566703; doi:10.3390/pharmaceutics17101271)
Supplement: Supplementary file 1 [file pharmaceutics-17-01271-s001.zip › pharmaceutics-3869870-supplementary.pdf]

SUPPLEMENTARY MATERIAL

Design, Synthesis, and Biological Evaluation of New Pyridine-2,5-Dicarboxylates Esters Bearing Natural Source Fragments as Anti-Trypanosomatid Agents

Luis M. Sánchez-Palestino[1,2,3], Adriana Moreno-Rodríguez[4], Diana V. Navarrete-Carriola[1,2], Marlet Martínez-Archundia3, Marhian Lopez-Vargas[4], Liliana Argueta-Figueroa[5], Lenci K. Vazquez-Jimenez[1], Alma D. Paz-Gonzalez[1], Eyra Ortiz-Perez[1], Michael P. Doyle[2*], Gildardo Rivera[1*]

[1]Laboratorio de Biotecnología Farmacéutica, Centro de Biotecnología Genómica, Instituto Politécnico nacional, Reynosa 88710, México; lsanchezp2000@alumno.ipn.mx (L.M.S.-P.); dnavarretec1900@alumno.ipn.mx (D.V.N.-C.); lenka.18@hotmail.com (L.K.V.-J.); apazg@ipn.mx (A.D.P.-G.); eortizp@ipn.mx (E.O.-P.); giriveras@ipn.mx (G.R.)

[2] Department of Chemistry, University of Texas at San Antonio, One UTSA Circle, San Antonio, TX 78249 USA; michael.doyle@utsa.edu (M.P.D.)

[3] Laboratory for the Design and Development of New Drugs and Biotechnological Innovation, Escuela Superior de Medicina, Instituto Politécnico Nacional, Plan de San Luis y Díaz Mirón, Ciudad de México, México; mtmartineza@ipn.mx (M.M.-A.);

[4] Laboratorio de Estudios Epidemiológicos, Clínicos, Diseños Experimentales e Investigación, Facultad de Ciencias Químicas, Universidad Autónoma "Benito Juárez" de Oaxaca, Avenida Universidad S/N, Ex Hacienda Cinco Señores, Oaxaca 68120, México, augl770623.fmc@uabjo.mx (A.M.-R.), mar-hianlv9@gmail.com (M.L.-V.)

[5] SECIHTI - Tecnológico Nacional de México-Instituto Tecnológico de Toluca, Metepec, 52149 Estado de México, México, liliana.argueta@secihti.mx (L.A.-F.)

*Correspondence: michael.doyle@utsa.edu (M.P.D.); giriveras@ipn.mx (G.R.).

CONTENTS

1. Spectral information 2
2. NMR spectra 10
3. Crystallographic information 41

1. Spectral information

Table S1. Analytical and spectral characterization of the pyridine compounds (3a-c – 12a-c). All compounds were prepared, purified and analyzed by similar procedures. Isomerization and, were appropriate, racemization were not detected.

Structure	Description
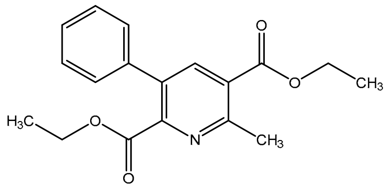	**Diethyl 6-methyl-3-phenylpyridine-2,5-dicarboxylate, 3a.** Yellow oil, 0.3 mmol, Y= 56%. Flash column chromatography conditions hexane: ethyl acetate = 3:1. **^{1}H-NMR** (500 MHz, $CDCl_3$) δ 8.27 (s, 1H), 7.48 – 7.42 (Comp, 3H), 7.38 (dd, *J* = 7.7, 1.8 Hz, 2H), 4.43 (q, *J* = 7.2 Hz, 2H), 4.22 (q, *J* = 7.2 Hz, 2H), 2.93 (s, 3H), 1.42 (t, *J* = 7.1 Hz, 3H), 1.07 (t, *J* = 7.2 Hz, 3H). **^{13}C-NMR** (126 MHz, $CDCl_3$) δ 166.8, 165.8, 158.4, 150.8, 140.4, 137.3, 134.0, 128.6, 128.3, 128.2, 126.8, 61.9, 61.7, 24.5, 14.3, 13.7. **HRMS** (ESI) m/z: $[M+H]^+$ Calculated for $C_{18}H_{19}NO_4$ 314.1387; Found 314.1386.
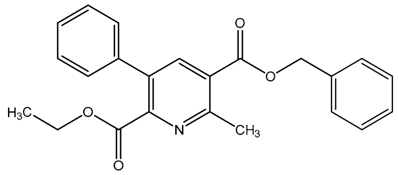	**5-Benzyl 2-ethyl 6-methyl-3-phenylpyridine-2,5-dicarboxylate, 4a.** Colorless oil, 0.3 mmol, Y= 72%. Flash column chromatography conditions hexane: ethyl acetate = 3:1.**^{1}H-NMR** (500 MHz, $CDCl_3$) δ 8.30 (s, 1H), 7.48 – 7.35 (Comp, 10H), 5.41 (s, 2H), 4.22 (q, *J* = 7.1 Hz, 2H), 2.94 (s, 3H), 1.07 (t, *J* = 7.2 Hz, 3H). **^{13}C-NMR** (126 MHz, CDCl3) δ 166.7, 165.7, 158.6, 151.1, 140.4, 137.2, 135.4, 134.0, 128.8, 128.6, 128.6, 128.3, 128.3, 126.4, 67.6, 61.7, 24.6, 13.7. **HRMS** (ESI) m/z: $[M+H]^+$ Calculated for $C_{23}H_{21}NO_4$ 376.1543; Found 376.1543.
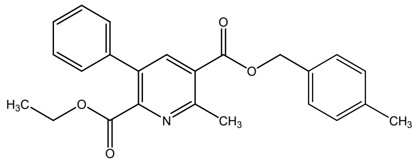	**2-Ethyl 5-(4-methylbenzyl) 6-methyl-3-phenylpyridine-2,5-dicarboxylate, 5a.** Colorless oil. Y= 74%. Flash column chromatography conditions hexane: ethyl acetate = 3:1. **^{1}H-NMR** (500 MHz, $CDCl_3$) δ 8.27 (s, 1H), 7.45 – 7.34 (Comp, 7H), 7.22 (d, *J* = 7.7 Hz, 2H), 5.36 (s, 2H), 4.21 (q, *J* = 7.1 Hz, 2H), 2.92 (s, 3H), 2.38 (s, 3H), 1.07 (t, *J* = 7.1 Hz, 3H). **^{13}C-NMR** (126 MHz, $CDCl_3$) δ 166.7, 165.7, 158.5, 151.0, 140.5, 138.5, 137.2, 134.0, 132.3, 129.4, 128.7, 128.6, 128.4, 128.2, 126.5, 67.4, 61.9, 24.5, 21.2, 13.7. **HRMS** (ESI) m/z: $[M+H]^+$ Calculated for $C_{24}H_{23}NO_4$ 390.1700; Found 390.1703.
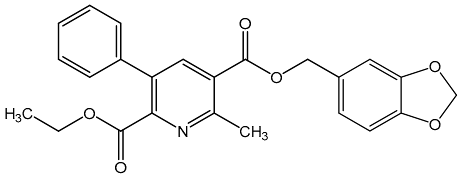	**5-(Benzo[d][1,3]dioxol-5-ylmethyl) 2-ethyl 6-methyl-3-phenylpyridine-2,5-dicarboxylate, 6a.** White solid, 0.3 mmol, Y= 71%. M.P= ND. Flash column chromatography conditions hexane: ethyl acetate = 3:1. **^{1}H-NMR** (300 MHz, $CDCl_3$) δ 8.3 (s, 1H), 7.4 (comp, 3H), 7.4 (comp, *J* = 7.3, 2.5 Hz, 2H), 6.9 (dd, *J* = 4.8, 1.7 Hz, 2H), 6.8 (d, *J* = 8.3 Hz, 1H), 6.0 (s, 2H), 5.3 (s, 2H), 4.2 (q, *J* = 7.2 Hz, 2H), 2.9 (s, 3H), 1.1 (t, *J* = 7.1 Hz, 3H). **^{13}C-NMR** (75 MHz, $CDCl_3$) δ 166.7, 165.6, 158.6, 151.0, 147.9, 140.4, 137.2, 133.9, 129.0, 128.6, 128.3, 128.3, 126.4, 122.7, 109.3, 108.4, 101.3, 67.4, 61.9, 29.7, 24.5, 13.7. **HRMS** (ESI) m/z: $[M+H]^+$ Calculated for $C_{24}H_{21}NO_6$ 420.1442; Found 420.1442.

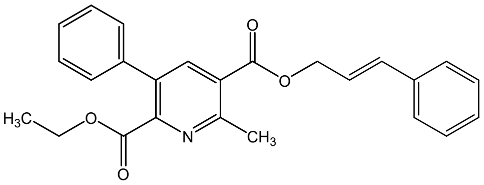	**5-Cinnamyl 2-ethyl 6-methyl-3-phenylpyridine-2,5-dicarboxylate, 7a.** Colorless oil, 0.3 mmol, Y= 66%. Flash column chromatography conditions hexane: ethyl acetate = 3:1. **^{1}H-NMR** (500 MHz, $CDCl_3$) δ 8.3 (s, 1H), 7.4 (Comp, 5H), 7.4 (dd, *J* = 7.8, 1.8 Hz, 2H), 7.4 (t, *J* = 7.4 Hz, 2H), 7.3 (m, 1H), 6.8 (d, *J* = 15.9 Hz, 1H), 6.5 – 6.4 (m, 1H), 5.0 (dd, *J* = 6.6, 1.4 Hz, 2H), 4.2 (q, *J* = 7.1 Hz, 2H), 3.0 (s, 3H), 1.1 (t, *J* = 7.1 Hz, 3H). **^{13}C-NMR** (126 MHz, $CDCl_3$) δ 166.7, 165.6, 158.6, 151.0, 140.5, 137.2, 136.0, 135.3, 134.0, 128.7, 128.6, 128.3, 128.3, 126.7, 126.5, 122.5, 66.3, 61.9, 24.5, 13.7. **HRMS** (ESI) m/z: $[M+H]^+$ Calculated for $C_{25}H_{23}NO_4$ 402.1700; Found 402.1699.
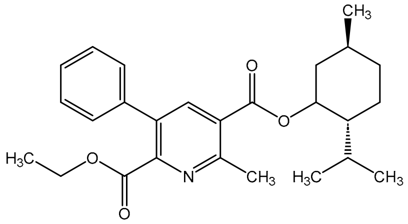	**2-Ethyl 5-((1*R*,2*R*,5*S*)-2-isopropyl-5-methylcyclohexyl) 6-methyl-3-phenylpyridine-2,5-dicarboxylate, 8a.** Colorless oil. 0.3 mmol. Y=80%. Flash column chromatography conditions hexane: ethyl acetate = 3:1. **^{1}H-NMR** (500 MHz, $CDCl_3$) δ 8.2 (s, 1H), 7.4 (q, 3H), 7.4 (d, *J* = 8.1 Hz, 2H), 5.0 (td, *J* = 10.9, 4.4 Hz, 1H), 4.2 (q, *J* = 7.2 Hz, 2H), 2.9 (s, 3H), 2.2 (d, *J* = 12.2 Hz, 1H), 1.9 (td, *J* = 7.0, 2.6 Hz, 1H), 1.8 (d, *J* = 11.4 Hz, 2H), 1.5 (t, *J* = 11.5 Hz, 2H), 1.2 (q, *J* = 12.0 Hz, 3H), 1.1 (t, *J* = 7.1 Hz, 3H), 1.0 (d, *J* = 6.6 Hz, 3H), 0.9 (d, *J* = 6.9 Hz, 3H), 0.8 (d, *J* = 6.9 Hz, 3H). **^{13}C-NMR** (126 MHz, CDC13) δ 166.77, 165.55, 158.18, 150.72, 140.09, 137.35, 133.91, 128.59, 128.35, 128.22, 127.33, 75.85, 61.83, 47.10, 40.98, 34.20, 31.64, 26.51, 24.45, 23.47, 22.03, 20.74, 16.32, 13.68. **HRMS** (ESI) m/z: $[M+H]^+$ Calculated for $C_{26}H_{33}NO_4$ 424.2482; Found 424.2485.
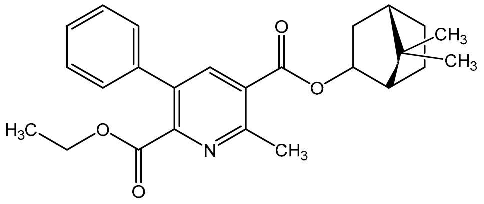	**2-Ethyl 5-((1*S*,2*R*,4*R*)-1,7,7-trimethylbicyclo[2.2.1]heptan-2-yl) 6-methyl-3-phenylpyridine-2,5-dicarboxylate, 9a.** Colorless oil, 0.3 mmol, Y= 75%. Flash column chromatography conditions hexane: ethyl acetate = 3:1. **^{1}H-NMR** (500 MHz, $CDCl_3$) δ 8.2 (s, 1H), 7.5 (Comp, 3H), 7.4 (d, *J* = 8.1 Hz, 2H), 5.2 – 5.2 (m, 1H), 4.2 (q, *J* = 7.2 Hz, 2H), 2.9 (s, 3H), 2.6 – 2.5 (m, 1H), 2.0 (ddd, *J* = 13.4, 9.5, 4.4 Hz, 1H), 1.9 – 1.8 (m, 1H), 1.8 (t, *J* = 4.5 Hz, 1H), 1.5 – 1.4 (m, 1H), 1.3 – 1.3 (m, 1H), 1.1 (dd, *J* = 13.9, 3.7 Hz, 1H), 1.1 (t, *J* = 7.2 Hz, 3H), 1.0 (s, 3H), 0.9 (d, *J* = 4.3 Hz, 6H). **^{13}C-NMR** (126 MHz, $CDCl_3$) δ 166.8, 166.4, 158.0, 150.8, 140.2, 137.3, 133.9, 128.6, 128.3, 128.3, 127.4, 82.0, 61.8, 49.0, 48.0, 44.9, 37.0, 28.1, 27.5, 24.7, 19.7, 18.9, 13.7, 13.7. **HRMS** (ESI) m/z: $[M+H]^+$ Calculated for $C_{26}H_{31}NO_4$ 422.2326; Found 422.2324.
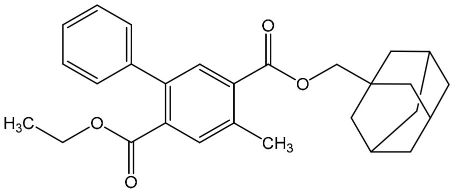	**5-(Adamantan-1-ylmethyl) 2-ethyl 6-methyl-3-phenylpyridine-2,5-dicarboxylate, 10a**: Colorless oil, 0.3 mmol, Y=75%. Flash column chromatography conditions hexane: ethyl acetate = 3:1. **^{1}H-NMR** (500 MHz, $CDCl_3$) δ 8.3 (s, 1H), 7.4 (t, *J* = 8.3 Hz, 3H), 7.4 (dd, *J* = 7.9, 1.8 Hz, 2H), 4.2 (q, *J* = 7.2 Hz, 2H), 4.0 (s, 2H), 2.9 (s, 3H), 2.0 (s, 3H), 1.8 (d, *J* = 12.2 Hz, 3H), 1.7 (d, *J* = 12.7 Hz, 3H), 1.6 (d, *J* = 3.2 Hz, 6H), 1.1 (t, *J* = 7.2 Hz, 3H). **^{13}C-NMR** (126 MHz, $CDCl_3$) δ 166.8, 166.2, 158.2, 150.9, 140.3, 137.3, 133.9, 128.6, 128.3, 128.3, 127.1, 75.4, 61.9, 39.5, 36.9, 33.5, 28.0, 24.8. **HRMS** (ESI) m/z: $[M+H]^+$ Calculated for $C_{27}H_{31}NO_4$ 434.2326; Found 434.2327.

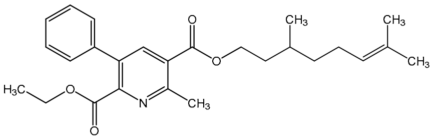	**5-(3,7-Dimethyloct-6-en-1-yl) 2-ethyl 6-methyl-3-phenylpyridine-2,5-dicarboxylate, 11a.** Colorless oil. 0.3 mmol. Y=85%. Flash column chromatography conditions hexane: ethyl acetate = 3:1. **^{1}H-NMR** (500 MHz, $CDCl_3$) δ 8.3 (s, 1H), 7.4 (t, *J* = 7.1 Hz, 3H), 7.4 (dd, *J* = 7.7, 1.8 Hz, 2H), 5.1 – 5.1 (m, 1H), 4.4 (comp, *J* = 10.9, 9.0, 5.0 Hz, 2H), 4.2 (q, *J* = 7.2 Hz, 2H), 2.9 (s, 3H), 2.0 (dt, *J* = 14.8, 7.4 Hz, 2H), 1.8 (ddd, *J* = 12.3, 7.1, 4.8 Hz, 1H), 1.7 (d, *J* = 1.5 Hz, 3H), 1.6 (Comp, 2H), 1.6 (s, 4H), 1.5 – 1.4 (m, 1H), 1.3 – 1.2 (m, 1H), 1.1 (t, *J* = 7.1 Hz, 3H), 1.0 (d, *J* = 6.3 Hz, 3H). **^{13}C-NMR** (126 MHz, $CDCl_3$) δ 166.8, 166.0, 158.3, 150.9, 140.4, 137.3, 133.9, 131.6, 128.6, 128.3, 128.2, 126.9, 124.5, 64.4, 61.9, 36.9, 35.5, 29.6, 25.7, 25.4, 24.5, 19.5, 17.7, 13.7. **HRMS** (ESI) m/z: $[M+H]^+$ Calculated for $C_{26}H_{33}NO_4$ 424.2482; Found 424.2483.
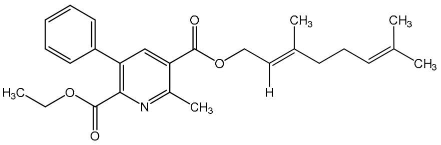	**(*E*)-5-(3,7-dimethylocta-2,6-dien-1-yl) 2-ethyl 6-methyl-3-phenylpyridine-2,5-dicarboxylate, 12a.** Colorless oil. 0.3 mmol. Y=89%. Flash column chromatography conditions hexane: ethyl acetate = 3:1. **^{1}H-NMR** (500 MHz, $CDCl_3$) δ 8.3 (s, 1H), 7.4 (comp, 2H), 7.4 (dd, *J* = 7.9, 1.7 Hz, 3H), 5.5 – 5.4 (m, 1H), 5.1 – 5.1 (m, 1H), 4.9 (d, *J* = 7.1 Hz, 3H), 4.2 (q, *J* = 7.1 Hz, 3H), 2.1 (comp, *J* = 13.4, 6.2 Hz, 4H), 1.8 (s, 3H), 1.7 (s, 2H), 1.6 (s, 2H), 1.1 (t, *J* = 7.1 Hz, 4H). **^{13}C-NMR** (126 MHz, $CDCl_3$) δ 166.8, 165.9, 158.4, 150.8, 143.3, 140.4, 137.3, 133.9, 132.0, 128.6, 128.3, 128.2, 126.9, 123.7, 117.9, 62.6, 61.9, 39.6, 26.2, 25.7, 24.5, 17.7, 16.6, 13.6. **HRMS** (ESI) m/z: $[M+H]^+$ Calculated for $C_{26}H_{31}NO_4$ 422.2326; Found 422.2328.
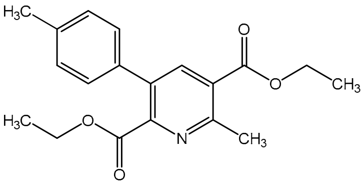	**Diethyl 6-methyl-3-(*p*-tolyl)pyridine-2,5-dicarboxylate, 3b.** Yellow oil, 0.3 mmol, Y= 95%. Flash column chromatography conditions hexane: ethyl acetate = 3:1. **^{1}H-NMR** (500 MHz, $CDCl_3$) δ 8.25 (s, 1H), 7.30 – 7.24 (comp, 4H), 4.42 (q, *J* = 7.2 Hz, 2H), 4.24 (q, *J* = 7.1 Hz, 2H), 2.92 (s, 3H), 2.42 (s, 3H), 1.42 (t, *J* = 7.1 Hz, 3H), 1.12 (t, *J* = 7.2 Hz, 3H). **^{13}C-NMR** (126 MHz, $CDCl_3$) δ 166.9, 165.9, 158.1, 150.8, 140.4, 138.2, 134.3, 133.9, 129.3, 128.2, 126.7, 61.8, 61.6, 24.4, 21.2, 14.3, 13.8. **HRMS** (ESI) m/z: $[M+H]^+$ Calculated for $C_{19}H_{21}NO_4$ 328.1543; Found 328.1537.
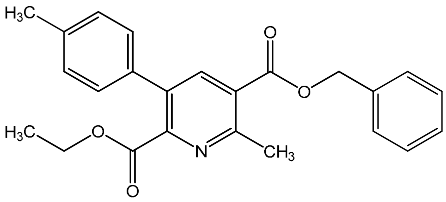	**5-Benzyl 2-ethyl 6-methyl-3-(p-tolyl)pyridine-2,5-dicarboxylate, 4b.** Colorless oil, 0.3 mmol, Y= 63%. Flash column chromatography conditions hexane: ethyl acetate = 3:1. **^{1}H-NMR** (500 MHz, $CDCl_3$) δ 8.3 (s, 1H), 7.5 (d, *J* = 6.6 Hz, 2H), 7.4 (comp, *J* = 13.1, 6.9 Hz, 3H), 7.3 (q, 4H), 5.4 (s, 2H), 4.2 (q, *J* = 7.2 Hz, 2H), 2.9 (s, 3H), 2.4 (s, 3H), 1.1 (t, *J* = 7.2 Hz, 3H). **^{13}C-NMR** (126 MHz, $CDCl_3$) δ 166.9, 165.7, 158.3, 151.1, 140.4, 138.2, 135.4, 134.2, 133.9, 129.3, 128.7, 128.5 (d, *J* = 12.3 Hz), 128.2, 126.4, 67.4, 61.9, 24.6, 21.2, 13.8.**HRMS** (ESI) m/z: $[M+H]^+$ Calculated for $C_{24}H_{23}NO_4$ 390.1700; Found 390.1699.
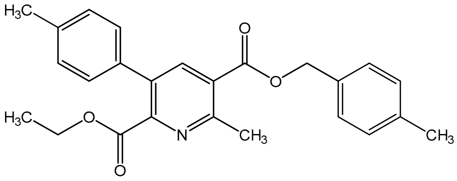	**2-Ethyl 5-(4-methylbenzyl) 6-methyl-3-(p-tolyl)pyridine-2,5-dicarboxylate, 5b.** Colorless oil. 0.3 mmol. Y= 86%. Flash column chromatography conditions hexane: ethyl acetate = 3:1. **^{1}H-NMR** (300 MHz, $CDCl_3$) δ 8.3 (s, 1H), 7.3 (d, *J* = 8.0 Hz, 2H), 7.3 (s, 4H), 7.2 (d, *J* = 7.9 Hz, 2H), 5.4 (s, 2H), 4.2 (q, *J* = 7.1 Hz, 2H), 2.9 (s, 3H), 2.4 (s, 3H), 2.4 (s, 3H), 1.1 (t, *J* = 7.1 Hz, 3H). **^{13}C-NMR** (75 MHz, $CDCl_3$) δ 166.9, 165.8, 158.3, 151.0, 140.4, 138.5, 138.2, 134.2, 133.9, 132.4, 129.4, 129.3, 128.6, 128.2, 126.5, 67.4, 61.9, 24.5, 21.2,

	13.8. **HRMS** (ESI) m/z: [M+H]$^{+}$ Calculated for $C_{25}H_{25}NO_4$ 404.1856; Found 404.1857.
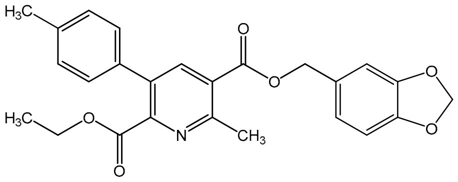	**5-(Benzo[d][1,3]dioxol-5-ylmethyl) 2-ethyl 6-methyl-3-(p-tolyl)pyridine-2,5-dicarboxylate, 6b.** Colorless oil, 0.3 mmol, Y= 71%. Flash column chromatography conditions hexane: ethyl acetate = 3:1. **^{1}H-NMR** (500 MHz, $CDCl_3$) δ 8.2 (s, 1H), 7.3 – 7.2 (m, 4H), 6.9 (d, *J* = 6.4 Hz, 2H), 6.8 (d, *J* = 8.4 Hz, 1H), 6.0 (s, 2H), 5.3 (s, 2H), 4.2 (q, *J* = 7.1 Hz, 2H), 2.9 (s, 3H), 2.4 (s, 3H), 1.1 (t, *J* = 7.1 Hz, 3H). **^{13}C-NMR** (126 MHz, $CDCl_3$) δ 166.8, 165.7, 158.2, 151.0, 147.9, 140.4, 138.2, 134.2, 133.8, 129.3, 129.1, 128.2, 126.4, 122.7, 109.2, 108.3, 101.3, 67.4, 61.8, 24.5, 21.2, 13.6. **HRMS** (ESI) m/z: [M+H]$^{+}$ Calculated for $C_{25}H_{23}NO_6$ 434.1598; Found 434.1597.
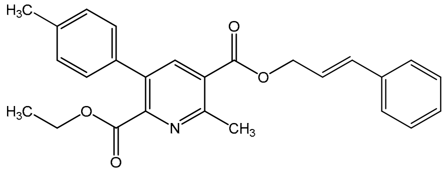	**5-Cinnamyl 2-ethyl 6-methyl-3-(p-tolyl)pyridine-2,5-dicarboxylate, 7b.** Colorless oil, 0.3 mmol, Y= 77%. Flash column chromatography conditions hexane: ethyl acetate = 3:1. **^{1}H-NMR** (500 MHz, $CDCl_3$) δ 8.3 (s, 1H), 7.4 (d, *J* = 7.0 Hz, 2H), 7.4 (t, *J* = 7.5 Hz, 2H), 7.3 (comp, 5H), 6.8 (d, *J* = 15.9 Hz, 1H), 6.4 (dt, *J* = 15.7, 6.6 Hz, 1H), 5.0 (d, *J* = 6.6 Hz, 2H), 4.3 (q, *J* = 7.1 Hz, 2H), 3.0 (s, 3H), 2.4 (s, 3H), 1.1 (t, *J* = 7.1 Hz, 3H). **^{13}C-NMR** (126 MHz, $CDCl_3$) δ 166.9, 165.6, 158.3, 151.0, 140.5, 138.2, 136.0, 135.2, 134.2, 133.9, 129.3, 128.7, 128.3, 128.2, 126.7, 126.4, 122.5, 66.3, 61.9, 24.5, 21.2, 13.8. **HRMS** (ESI) m/z: [M+H]$^{+}$ Calculated for $C_{26}H_{25}NO_4$ 416.1856; Found 416.1865.
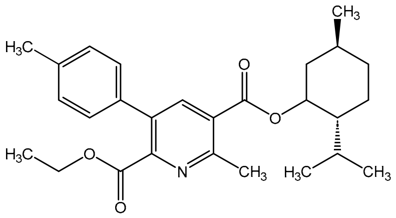	**2-Ethyl 5-((1*R*,2*R*,5*S*)-2-isopropyl-5-methylcyclohexyl) 6-methyl-3-(p-tolyl)pyridine-2,5-dicarboxylate, 8b.** Colorless oil. 0.3 mmol. Y=75%. Flash column chromatography conditions hexane: ethyl acetate = 3:1. **^{1}H-NMR** (300 MHz, $CDCl_3$) δ 8.2 (s, 1H), 7.3 (s, 4H), 5.0 (td, *J* = 10.9, 4.4 Hz, 1H), 4.3 (q, 2H), 2.9 (s, 3H), 2.4 (s, 3H), 2.1 (d, *J* = 12.0 Hz, 1H), 2.0 – 1.9 (m, 1H), 1.8 (d, *J* = 11.6 Hz, 2H), 1.6 (comp, 3H), 1.1 (q, *J* = 6.6 Hz, 5H), 1.0 (d, *J* = 6.5 Hz, 3H), 0.9 (d, *J* = 7.0 Hz, 3H), 0.8 (d, *J* = 6.9 Hz, 3H). **^{13}C-NMR** (75 MHz, $CDCl_3$) δ 166.9, 165.6, 157.9, 150.7, 140.1, 138.2, 134.3, 133.8, 129.3, 128.2, 127.3, 75.8, 61.8, 47.2, 41.0, 34.2, 31.5, 26.5, 24.5, 23.5, 22.0, 21.2, 20.7, 16.4, 13.8. **HRMS** (ESI) m/z: [M+H]$^{+}$ Calculated for $C_{27}H_{35}NO_4$ 438.2639; Found 438.2640.
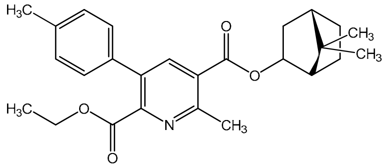	**2-Ethyl 5-((1*S*,2*R*,4*R*)-1,7,7-trimethylbicyclo[2.2.1]heptan-2-yl) 6-methyl-3-(p-tolyl)pyridine-2,5-dicarboxylate, 9b.** Yellow oil, 0.3 mmol, Y= 99%. Flash column chromatography conditions hexane: ethyl acetate = 3:1. **^{1}H-NMR** (500 MHz, $CDCl_3$) δ 8.2 (s, 1H), 7.3 (t, *J* = 6.6 Hz, 4H), 5.2 (dt, *J* = 9.9, 2.9 Hz, 1H), 4.2 (q, *J* = 7.2 Hz, 2H), 2.9 (s, 3H), 2.5 (ddt, *J* = 14.0, 9.9, 4.0 Hz, 1H), 2.4 (s, 3H), 2.0 (ddd, *J* = 13.4, 9.4, 4.4 Hz, 1H), 1.8 (dp, *J* = 11.9, 4.2 Hz, 1H), 1.8 (t, *J* = 4.6 Hz, 1H), 1.5 – 1.4 (m, 1H), 1.3 (comp, 2H), 1.1 (t, *J* = 7.2 Hz, 3H), 1.0 (s, 3H), 0.9 (d, *J* = 3.4 Hz, 6H). **^{13}C-NMR** (126 MHz, $CDCl_3$) δ 166.9, 166.5, 157.7, 150.8, 140.2, 138.2, 134.3, 133.8, 129.4, 128.2, 127.3, 81.9, 61.8, 49.0, 48.0, 44.9, 37.0, 28.1, 27.5, 24.6, 21.2, 19.7, 18.9, 13.8, 13.7. **HRMS** (ESI) m/z: [M+H]$^{+}$ Calculated for $C_{27}H_{33}NO_4$ 436.2482; Found 436.2480.

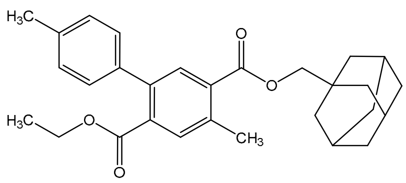	**5-(Adamantan-1-ylmethyl) 2-ethyl 6-methyl-3-(p-tolyl)pyridine-2,5-dicarboxylate, 10b.** Colorless oil, 0.3 mmol, Y= 72%. Flash column chromatography conditions hexane: ethyl acetate = 3:1. **^{1}H-NMR** (500 MHz, $CDCl_3$) δ 8.26 (s, 1H), 7.31 – 7.25 (q, 4H), 4.25 (q, *J* = 7.1 Hz, 2H), 3.98 (s, 2H), 2.93 (s, 3H), 2.43 (s, 3H), 2.03 (s, 3H), 1.77 (d, *J* = 12.2 Hz, 3H), 1.68 (d, *J* = 11.0 Hz, 3H), 1.63 (d, *J* = 3.2 Hz, 6H), 1.13 (t, *J* = 7.2 Hz, 3H). **^{13}C-NMR** (126 MHz, $CDCl_3$) δ 166.9, 166.3, 157.9, 150.9, 140.3, 138.2, 134.3, 133.8, 129.4, 128.2, 127.1, 75.4, 61.7, 39.5, 36.9, 33.5, 28.0, 24.7, 21.2, 13.8. **HRMS** (ESI) m/z: [M+H]$^+$ Calculated for $C_{28}H_{33}NO_4$ 448.2482; Found 448.2480.
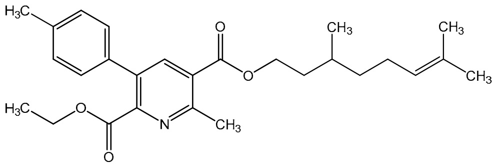	**5-(3,7-Dimethyloct-6-en-1-yl) 2-ethyl 6-methyl-3-(p-tolyl)pyridine-2,5-dicarboxylate, 11b.** Colorless oil. 0.3 mmol. Y= 67%. Flash column chromatography conditions hexane: ethyl acetate = 3:1. **^{1}H-NMR** (300 MHz, $CDCl_3$) δ 8.2 (s, 1H), 7.3 (d, *J* = 1.5 Hz, 4H), 5.1 – 5.1 (m, 1H), 4.4 (comp, 2H), 4.2 (q, *J* = 7.1 Hz, 2H), 2.9 (s, 3H), 2.4 (s, 3H), 2.0 (p, *J* = 7.1 Hz, 2H), 1.9 – 1.8 (m, 1H), 1.6 (d, 8H), 1.5 – 1.3 (m, 1H), 1.3 – 1.2 (m, 1H), 1.1 (t, *J* = 7.1 Hz, 3H), 1.0 (d, *J* = 6.3 Hz, 3H). **^{13}C-NMR** (75 MHz, $CDCl_3$) δ 166.9, 166.0, 158.0, 150.9, 140.4, 138.2, 134.3, 133.9, 131.5, 129.3, 128.2, 126.8, 124.4, 64.3, 61.9, 37.0, 35.5, 29.6, 25.7, 25.4, 24.5, 21.2, 19.5, 17.7, 13.8. **HRMS** (ESI) m/z: [M+H] $^+$ Calculated for $C_{27}H_{35}NO_4$ 438.2639; Found 438.2641.
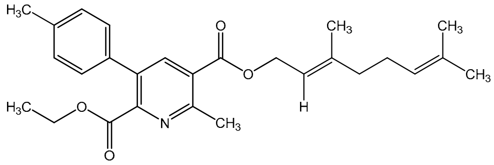	**(*E*)-5-(3,7-Dimethylocta-2,6-dien-1-yl) 2-ethyl 6-methyl-3-(p-tolyl)pyridine-2,5-dicarboxylate, 12b.** Colorless oil. 0.3mmol. Y=77%. Flash column chromatography conditions hexane: ethyl acetate = 3:1. **^{1}H-NMR** (300 MHz, $CDCl_3$) δ 8.2 (s, 1H), 7.3 (d, *J* = 1.5 Hz, 4H), 5.5 (td, *J* = 7.3, 1.4 Hz, 1H), 5.1 – 5.1 (m, 1H), 4.9 (d, *J* = 7.2 Hz, 2H), 4.2 (q, *J* = 7.1 Hz, 2H), 2.9 (s, 3H), 2.4 (s, 3H), 2.1 (d, *J* = 5.9 Hz, 4H), 1.8 (s, 3H), 1.7 (s, 3H), 1.6 (s, 3H), 1.1 (t, *J* = 7.1 Hz, 3H). **^{13}C-NMR** (75 MHz, $CDCl_3$) δ 166.9, 166.0, 158.1, 150.8, 143.2, 140.4, 138.1, 134.3, 133.9, 131.9, 129.2, 128.2, 126.8, 123.6, 117.8, 62.5, 61.8, 39.5, 26.2, 25.7, 24.4, 21.2, 17.7, 16.6, 13.8. **HRMS** (ESI) m/z: [M+H]$^+$ Calculated for $C_{27}H_{33}NO_4$ m/z: 436.2482; Found 436.2485.
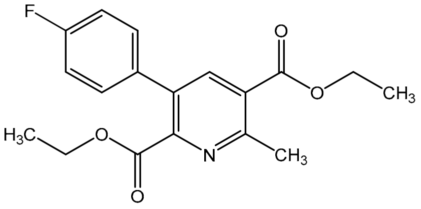	**Diethyl 3-(4-fluorophenyl)-6-methylpyridine-2,5-dicarboxylate, 3c**. Yellow oil, 0.3 mmol, Y= 68%. Flash column chromatography conditions hexane: ethyl acetate = 3:1. **^{1}H-NMR** (500 MHz, $CDCl_3$) δ 8.2 (s, 1H), 7.4 (q, 2H), 7.1 (t, *J* = 8.7 Hz, 2H), 4.4 (p, *J* = 7.1 Hz, 2H), 4.2 (q, *J* = 7.2 Hz, 2H), 2.9 (s, 3H), 1.4 (t, *J* = 7.1 Hz, 3H), 1.1 (t, *J* = 7.1 Hz, 3H). **^{13}C-NMR** (126 MHz, $CDCl_3$) δ 166.6, 165.8, 163.8, 161.9, 158.6, 150.7, 140.4, 133.3, 133.3, 132.9, 130.2, 130.1, 126.9, 115.7, 115.6, 61.9, 61.7, 24.5, 14.3, 13.8. **HRMS** (ESI) m/z: [M+H]$^+$ Calculated for $C_{18}H_{18}FNO_4$ 332.1293; Found 332.1292.
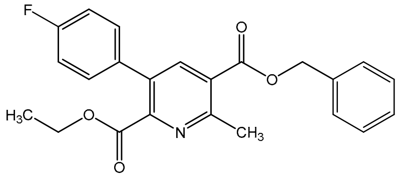	**5-Benzyl 2-ethyl 3-(4-fluorophenyl)-6-methylpyridine-2,5-dicarboxylate, 4c.** Colorless oil, 0.3 mmol, Y= 71%. Flash column chromatography conditions hexane: ethyl acetate = 3:1. **^{1}H-NMR** (500 MHz, $CDCl_3$) δ 8.2 (s, 1H), 7.4 (comp, 5H), 7.3 (comp, 2H), 7.1 (p, *J* = 8.7 Hz, 2H), 5.4 (s, 2H), 4.2 (q, *J* = 7.2 Hz, 2H), 2.9 (s, 3H), 1.1 (t, *J* = 7.1 Hz, 3H). **^{13}C-NMR** (126 MHz, $CDCl_3$) δ 166.5,

	165.6, 163.9, 161.9, 158.8, 151.0, 140.4, 135.3, 133.2, 133.2, 132.9, 130.2, 130.1, 128.8, 128.6, 128.5, 126.5, 115.7, 115.6, 67.5, 62.0, 24.6, 13.8. **HRMS** (ESI) m/z: $[M+H]^+$ Calculated for $C_{23}H_{20}NO_4$ 394.1449; Found 394.1450.
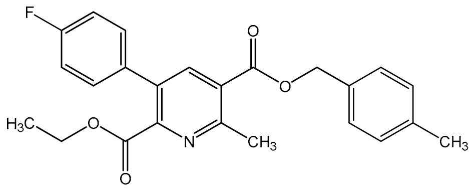	**2-Ethyl 5-(4-methylbenzyl) 3-(4-fluorophenyl)-6-methylpyridine-2,5-dicarboxylate, 5c.** Colorless oil, 0.3 mmol, Y= 68%. Flash column chromatography conditions hexane: ethyl acetate = 3:1. **^{1}H-NMR** (500 MHz, $CDCl_3$) δ 8.2 (s, 1H), 7.3 (comp, 4H), 7.2 (d, *J* = 8.1 Hz, 2H), 7.1 (t, *J* = 8.6 Hz, 2H), 5.4 (s, 2H), 4.2 (q, *J* = 7.1 Hz, 2H), 2.9 (s, 3H), 2.4 (s, 3H), 1.1 (t, *J* = 7.2 Hz, 3H). **^{13}C-NMR** (126 MHz, $CDCl_3$) δ 166.5, 165.6, 163.8, 161.9, 158.7, 150.9, 140.4, 138.6, 133.2, 133.2, 133.0, 132.3, 130.2, 130.1, 129.4, 128.7, 126.6, 115.7, 115.5, 67.5, 62.0, 24.6, 21.3, 13.8. **HRMS** (ESI) m/z: $[M+H]^+$ Calculated for $C_{24}H_{22}FNO_4$ 408.1606; Found 408.1597.
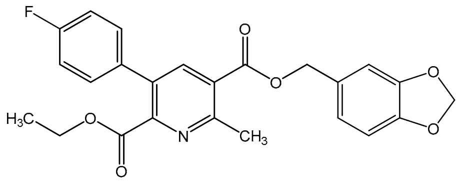	**5-(Benzo[d][1,3]dioxol-5-ylmethyl) 2-ethyl 3-(4-fluorophenyl)-6-methylpyridine-2,5-dicarboxylate, 6c.** Colorless oil, 0.3 mmol, Y= 75%. Flash column chromatography conditions hexane: ethyl acetate = 3:1. **^{1}H-NMR** (500 MHz, $CDCl_3$) δ 8.2 (s, 1H), 7.3 (comp, 2H), 7.1 (t, *J* = 8.7 Hz, 2H), 6.9 (dd, *J* = 5.3, 1.8 Hz, 2H), 6.8 (d, *J* = 8.4 Hz, 1H), 6.0 (s, 2H), 5.3 (s, 2H), 4.2 (q, *J* = 7.1 Hz, 2H), 2.9 (s, 3H), 1.1 (t, *J* = 7.2 Hz, 3H). **^{13}C-NMR** (126 MHz, $CDCl_3$) δ 166.5, 165.6, 163.8, 161.9, 158.7, 150.9, 148.0, 147.9, 140.4, 133.2, 133.2, 132.9, 129.0, 126.5, 122.8, 115.7, 115.5, 109.3, 108.4, 101.3, 67.5, 62.0, 24.6, 13.8. **HRMS** (ESI) m/z: $[M+H]^+$ Calculated for $C_{24}H_{20}FNO_6$ 438.1347; Found 438.1351.
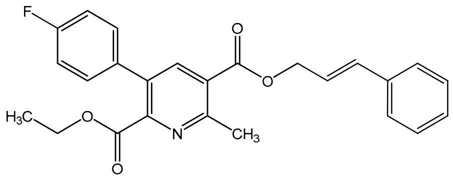	**5-Cinnamyl 2-ethyl 3-(4-fluorophenyl)-6-methylpyridine-2,5-dicarboxylate, 7c.** Colorless oil, 0.3 mmol, Y= 75%. Flash column chromatography conditions hexane: ethyl acetate = 3:1. **^{1}H-NMR** (500 MHz, $CDCl_3$) δ 8.28 (s, 1H), 7.43 (d, *J* = 8.1 Hz, 2H), 7.35 (q, *J* = 8.2 Hz, 4H), 7.28 (t, *J* = 7.3 Hz, 1H), 7.14 (t, *J* = 8.5 Hz, 2H), 6.77 (d, *J* = 15.7 Hz, 1H), 6.41 (dt, *J* = 15.9, 6.6 Hz, 1H), 5.02 (d, *J* = 6.6 Hz, 2H), 4.24 (q, *J* = 7.1 Hz, 2H), 2.96 (s, 3H), 1.13 (t, *J* = 7.1 Hz, 3H). **^{13}C-NMR** (126 MHz, $CDCl_3$) δ 166.5, 165.5, 163.8, 161.9, 158.8, 150.9, 140.5, 135.9, 135.4, 133.2, 133.2, 133.0, 130.2, 130.1, 128.7, 128.4, 126.7, 126.5, 122.4, 115.7, 115.6, 66.4, 62.0, 24.5, 13.8. **HRMS** (ESI) m/z: $[M+H]^+$ Calculated for $C_{25}H_{22}FNO_4$ 420.1606; Found 420.1605.
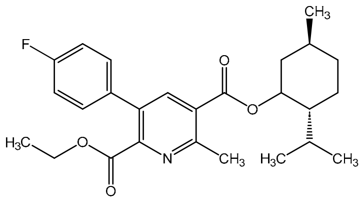	**2-Ethyl 5-((1*R*,2*S*,5*S*)-2-isopropyl-5-methylcyclohexyl) 3-(4-fluorophenyl)-6-methylpyridine-2,5-dicarboxylate, 8c.** Colorless oil, 0.3 mmol, Y= 73%. Flash column chromatography conditions hexane: ethyl acetate = 3:1. **^{1}H-NMR** (500 MHz, $CDCl_3$) δ 8.2 (s, 1H), 7.4 (comp, 2H), 7.2 (t, *J* = 8.7 Hz, 2H), 5.0 (td, *J* = 11.0, 4.5 Hz, 1H), 4.2 (q, *J* = 7.2 Hz, 2H), 2.9 (s, 3H), 2.2 – 2.1 (m, 1H), 1.9 (pd, *J* = 7.0, 2.8 Hz, 1H), 1.8 (dt, *J* = 14.6, 3.1 Hz, 2H), 1.6 – 1.5 (m, 1H), 1.2 (dd, 3H), 1.1 (t, *J* = 7.2 Hz, 3H), 0.9 (dd, *J* = 17.0, 6.8 Hz, 8H), 0.8 (d, *J* = 7.0 Hz, 3H). **^{13}C-NMR** (126 MHz, $CDCl_3$) δ 166.6, 165.5, 163.8, 161.9, 158.3, 150.6, 140.1, 133.4, 133.3, 132.9, 130.2, 130.1, 127.4, 115.7, 115.5, 75.9, 61.9, 47.1, 41.0, 34.2, 31.5, 26.5, 24.6, 23.5, 22.0, 20.8, 16.4, 13.8. **HRMS** (ESI) m/z: $[M+H]^+$ Calculated for $C_{26}H_{32}FNO_4$ 442.2388; Found 442.2379.

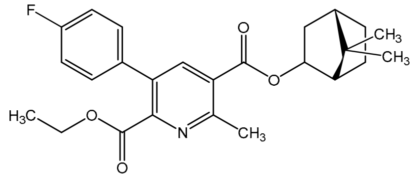	**2-Ethyl 5-((1*S*,2*R*,4*R*)-1,7,7-trimethylbicyclo[2.2.1]heptan-2-yl) 3-(4-fluorophenyl)-6-methylpyridine-2,5-dicarboxylate, 9c.** Yellow oil, 0.3 mmol, Y= 81%. Flash column chromatography conditions hexane: ethyl acetate = 3:1. **^{1}H-NMR** (500 MHz, $CDCl_3$) δ 8.2 (s, 1H), 7.4 (p, 2H), 7.2 (t, *J* = 8.7 Hz, 2H), 5.2 (td, 1H), 4.2 (q, *J* = 7.1 Hz, 2H), 2.9 (s, 3H), 2.5 (ddt, *J* = 13.7, 9.9, 4.0 Hz, 1H), 2.0 (ddd, *J* = 13.3, 9.5, 4.3 Hz, 1H), 1.8 (ddq, *J* = 12.2, 7.8, 4.0 Hz, 1H), 1.8 (t, *J* = 4.5 Hz, 1H), 1.5 – 1.4 (m, 1H), 1.3 (ddd, *J* = 11.9, 9.4, 4.4 Hz, 1H), 1.1 (dd, 1H), 1.1 (t, *J* = 7.0 Hz, 3H), 1.0 (s, 3H), 0.9 (s, 6H). **^{13}C-NMR** (126 MHz, $CDCl_3$) δ 166.6, 166.4, 163.9, 161.9, 158.2, 150.7, 140.2, 133.3, 133.3, 132.9, 130.1, 130.1, 127.4, 115.8, 115.6, 82.1, 61.9, 49.1, 48.0, 44.9, 37.0, 28.1, 27.5, 24.7, 19.7, 18.9, 13.8, 13.7. **HRMS** (ESI) m/z: $[M+H]^+$ Calculated for $C_{26}H_{30}FNO_4$ 440.2232; Found 440.2230.
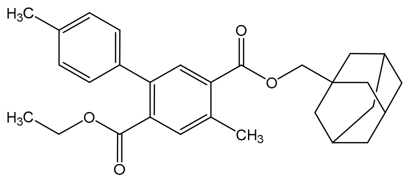	**5-(Adamantan-1-ylmethyl) 2-ethyl 3-(4-fluorophenyl)-6-methylpyridine-2,5-dicarboxylate, 10c.** Colorless oil, 0.3 mmol, Y= 95%. Flash column chromatography conditions hexane: ethyl acetate = 3:1. **^{1}H-NMR** (500 MHz, $CDCl_3$) δ 8.2 (s, 1H), 7.4 (p, 2H), 7.2 (t, *J* = 8.6 Hz, 2H), 4.2 (q, *J* = 7.1 Hz, 2H), 4.0 (s, 2H), 2.9 (s, 3H), 2.0 (s, 3H), 1.8 (d, *J* = 12.2 Hz, 3H), 1.7 (d, *J* = 12.5 Hz, 3H), 1.6 (d, *J* = 3.2 Hz, 6H), 1.1 (t, *J* = 7.1 Hz, 3H). **^{13}C-NMR** (126 MHz, $CDCl_3$) δ 166.6, 166.2, 163.9, 161.9, 158.3, 150.8, 140.3, 133.3, 133.3, 132.9, 130.1, 130.1, 127.2, 115.8, 115.6, 75.5, 62.0, 39.5, 36.9, 33.5, 28.0, 24.8. **HRMS** (ESI) m/z: $[M+H]^+$ Calculated for $C_{27}H_{30}FNO_4$ 452.2232; Found 452.2235.
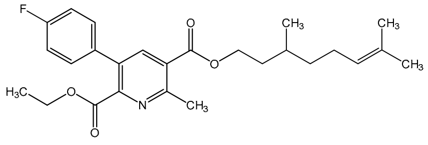	**5-(3,7-Dimethyloct-6-en-1-yl) 2-ethyl 3-(4-fluorophenyl)-6-methylpyridine-2,5-dicarboxylate, 11c.** Colorless oil, 0.3 mmol, Y= 70%. Flash column chromatography conditions hexane: ethyl acetate = 3:1. **^{1}H-NMR** (500 MHz, $CDCl_3$) δ 8.2 (s, 1H), 7.3 (p, 2H), 7.1 (t, *J* = 8.7 Hz, 2H), 5.1 (td, *J* = 7.2, 1.4 Hz, 1H), 4.4 (comp, 2H), 4.2 (q, *J* = 7.1 Hz, 2H), 2.9 (s, 3H), 2.0 (tp, *J* = 14.3, 7.2 Hz, 2H), 1.9 – 1.8 (m, 1H), 1.7 (s, 3H), 1.6 (comp, 5H), 1.4 – 1.4 (m, 1H), 1.3 – 1.2 (m, 1H), 1.1 (t, *J* = 7.2 Hz, 3H), 1.0 (d, *J* = 6.4 Hz, 3H). **^{13}C-NMR** (126 MHz, $CDCl_3$) δ 166.6, 165.9, 163.8, 161.9, 158.5, 150.8, 140.4, 133.3, 133.3, 132.9, 131.5, 130.1, 130.1, 126.9, 124.4, 115.7, 115.6, 64.4, 62.0, 37.0, 35.4, 29.6, 25.7, 25.4, 24.5, 19.5, 17.7, 13.7. **HRMS** (ESI) m/z: $[M+H]^+$ Calculated for $C_{26}H_{32}FNO_4$ 442.2388; Found 442.2379.
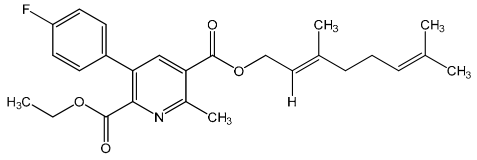	**(*E*)-5-(3,7-Dimethylocta-2,6-dien-1-yl) 2-ethyl 3-(4-fluorophenyl)-6-methylpyridine-2,5-dicarboxylate, 12c.** Colorless oil, 0.3 mmol, Y= 79%. Flash column chromatography conditions hexane: ethyl acetate = 3:1. **^{1}H-NMR** (500 MHz, $CDCl_3$) δ 8.2 (s, 1H), 7.3 (p, 2H), 7.1 (t, *J* = 8.8 Hz, 2H), 5.5 (t, *J* = 7.9 Hz, 1H), 5.1 (t, 1H), 4.9 (d, *J* = 7.2 Hz, 2H), 4.2 (q, *J* = 7.1 Hz, 2H), 2.9 (s, 3H), 2.1 (comp, *J* = 12.4, 6.3 Hz, 4H), 1.8 (s, 3H), 1.7 (s, 3H), 1.6 (s, 3H), 1.1 (t, *J* = 7.2 Hz, 3H). **^{13}C-NMR** (126 MHz, $CDCl_3$) δ 166.6, 165.8, 163.8, 161.9, 158.5, 150.7, 143.3, 140.4, 133.3, 133.3, 132.9, 132.0, 130.2, 130.1, 126.9, 123.6, 117.8, 115.7, 115.5,

	62.6, 61.9, 39.5, 26.2, 25.7, 24.5, 17.7, 16.6, 13.8. **HRMS** (ESI) m/z: $[M+H]^+$ Calculated for $C_{26}H_{30}FNO_4$ 440.2232; Found 440.2226.

2. NMR spectra

Figure S1. ^{1}H-NMR (500 MHz, $CDCl_3$) of compound **3a**

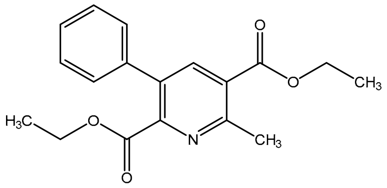

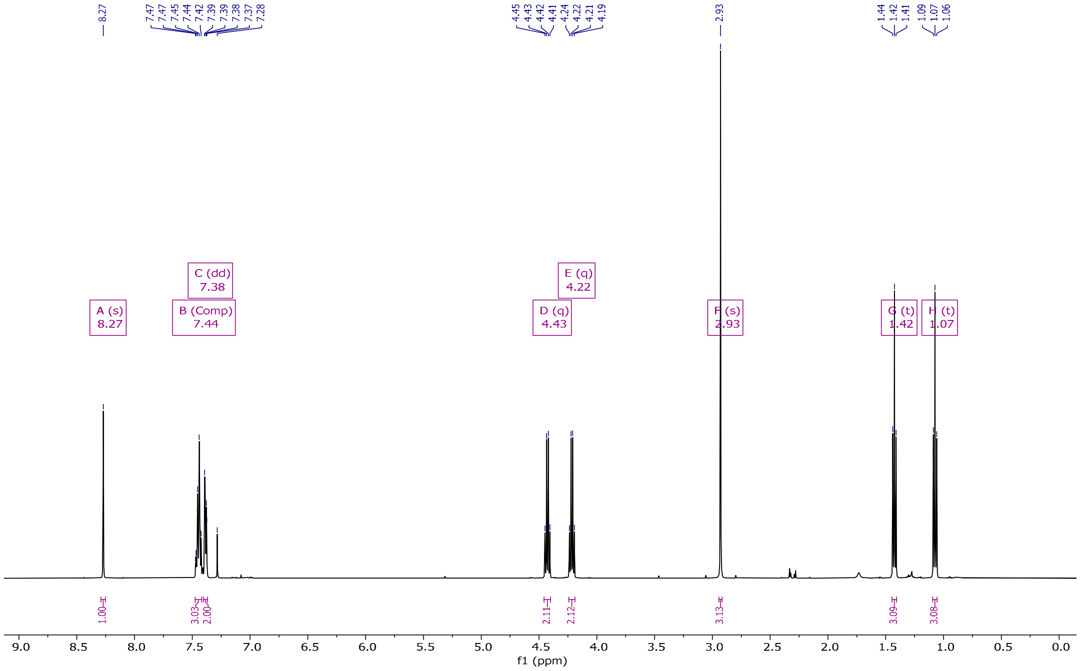

Figure S2. ^{13}C-NMR (126 MHz, $CDCl_3$) of compound **3a**

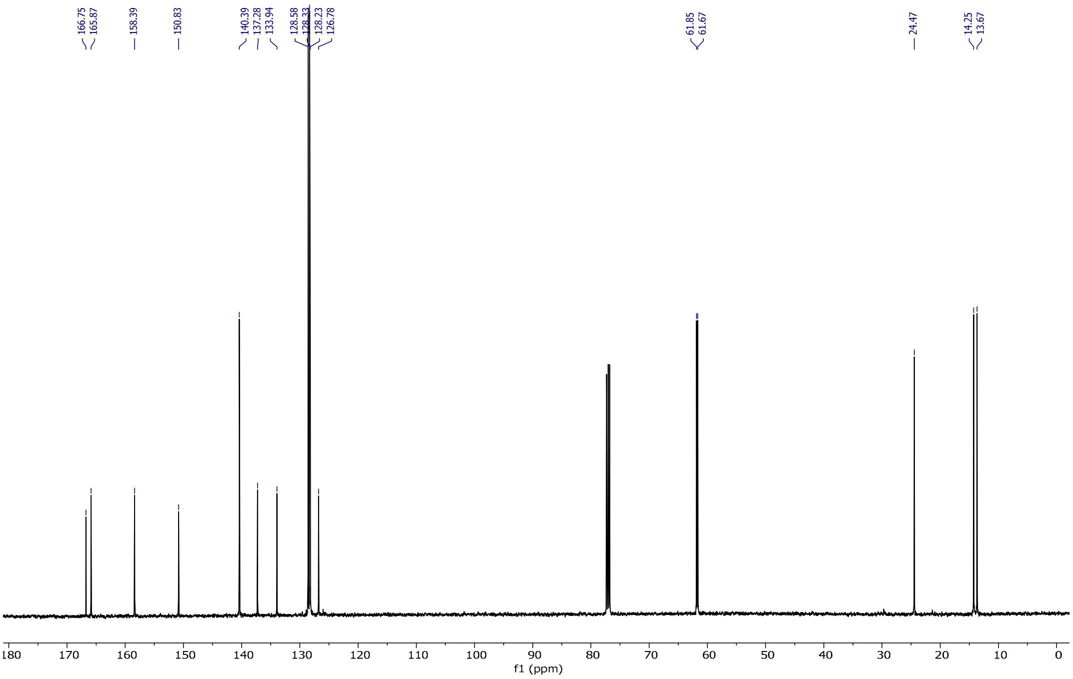

Figure S3. ^{1}H-NMR (500 MHz, $CDCl_3$) of compound **4a**

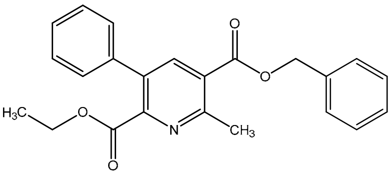

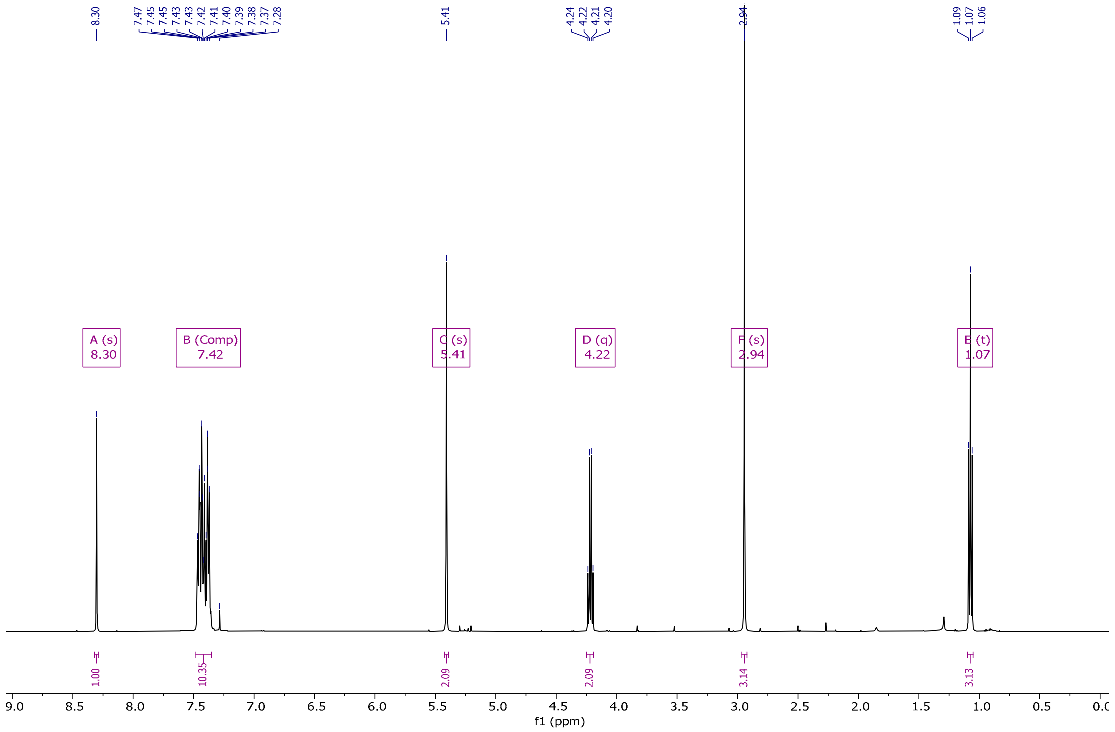

Figure S4. ^{13}C-NMR (126 MHz, $CDCl_3$) of compound **4a**

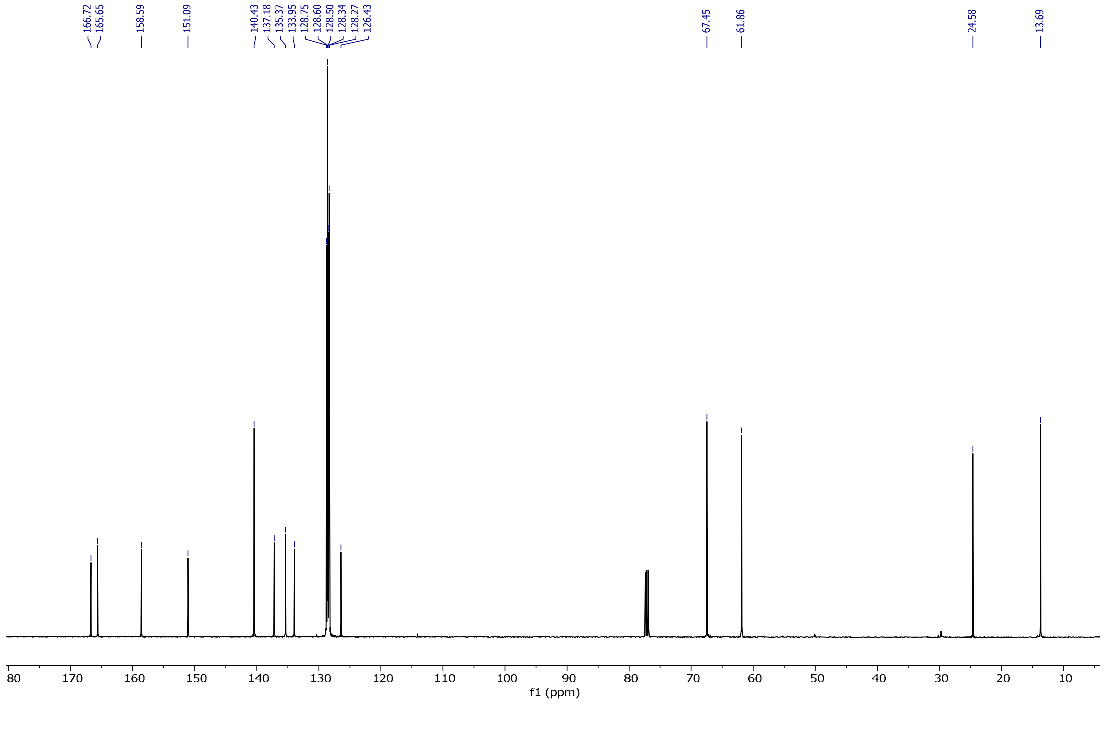

Figure S5. ¹H-NMR (500 MHz, $CDCl_3$) of compound **5a**

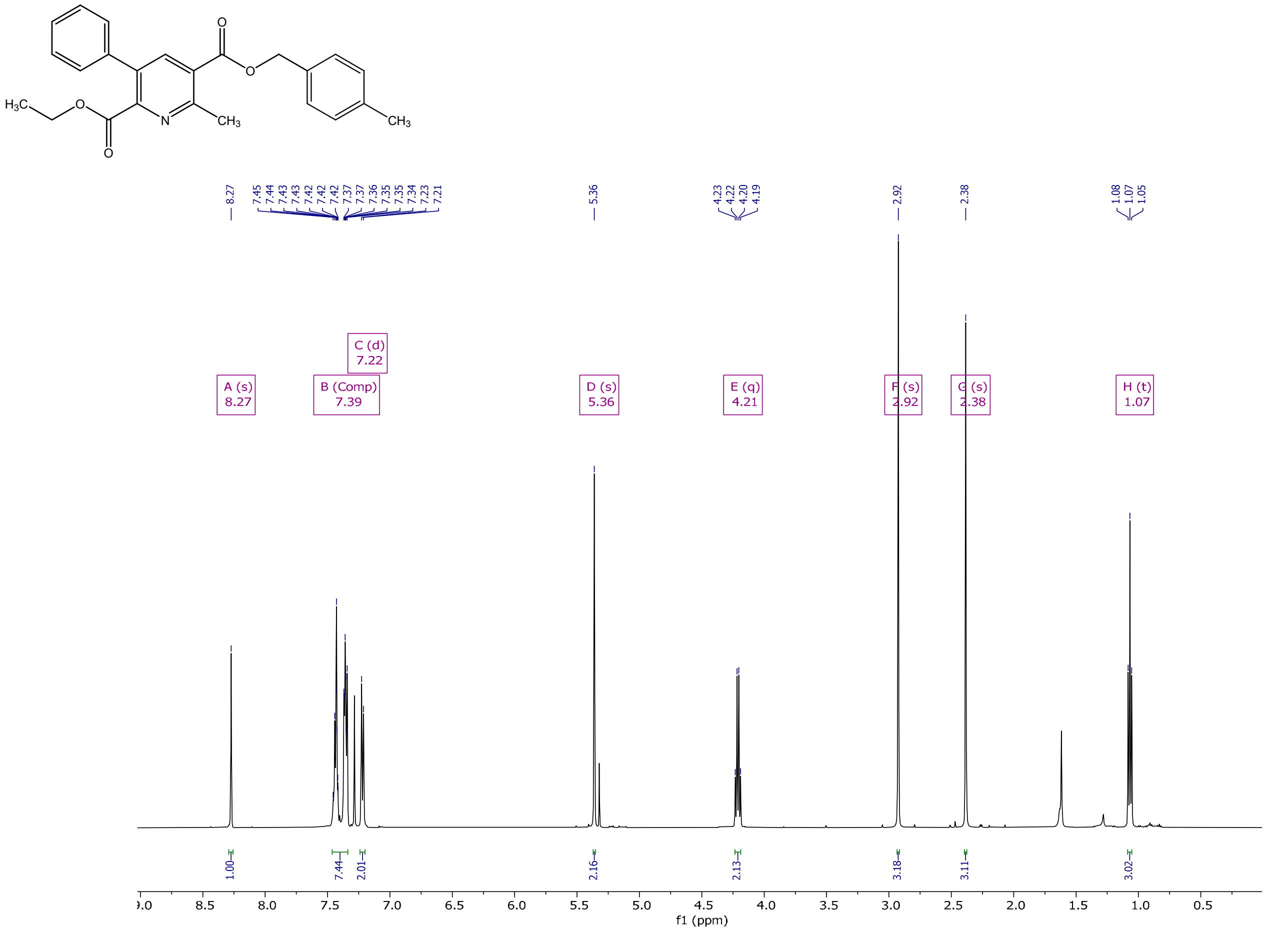

Figure S6. ¹³C-NMR (126 MHz, $CDCl_3$) of compound **5a**

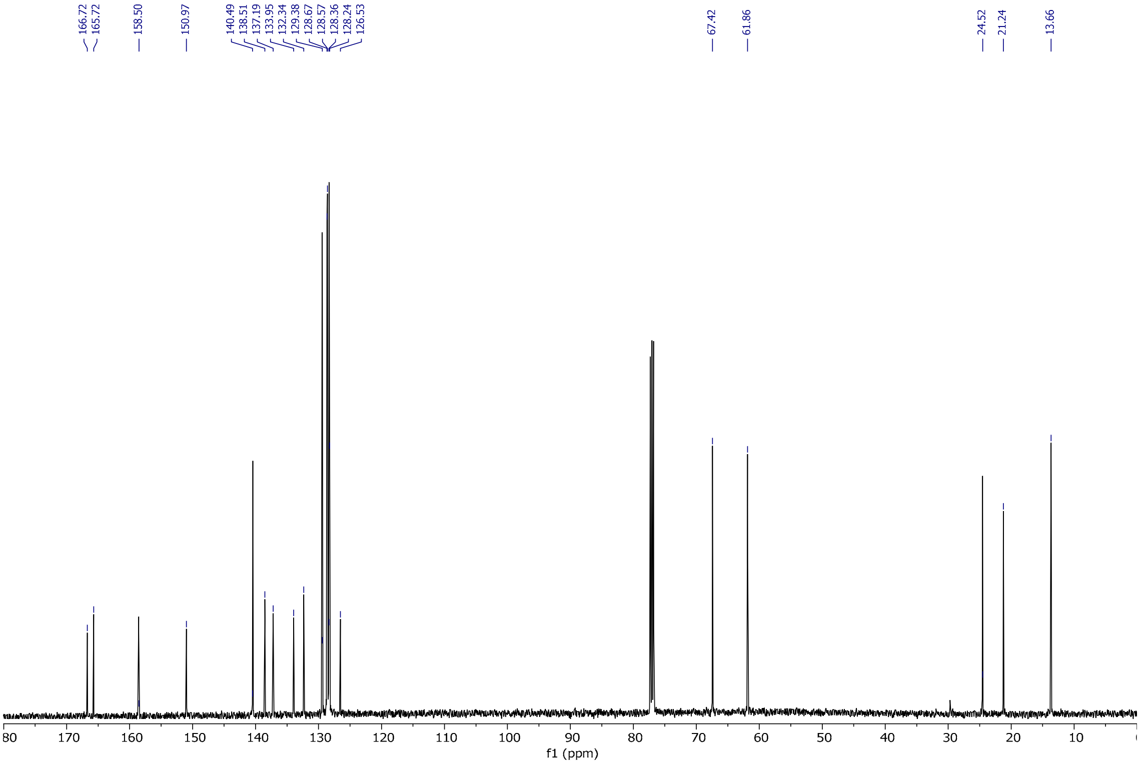

Figure S7. ^{1}H-NMR (300 MHz, $CDCl_3$) of compound **6a**

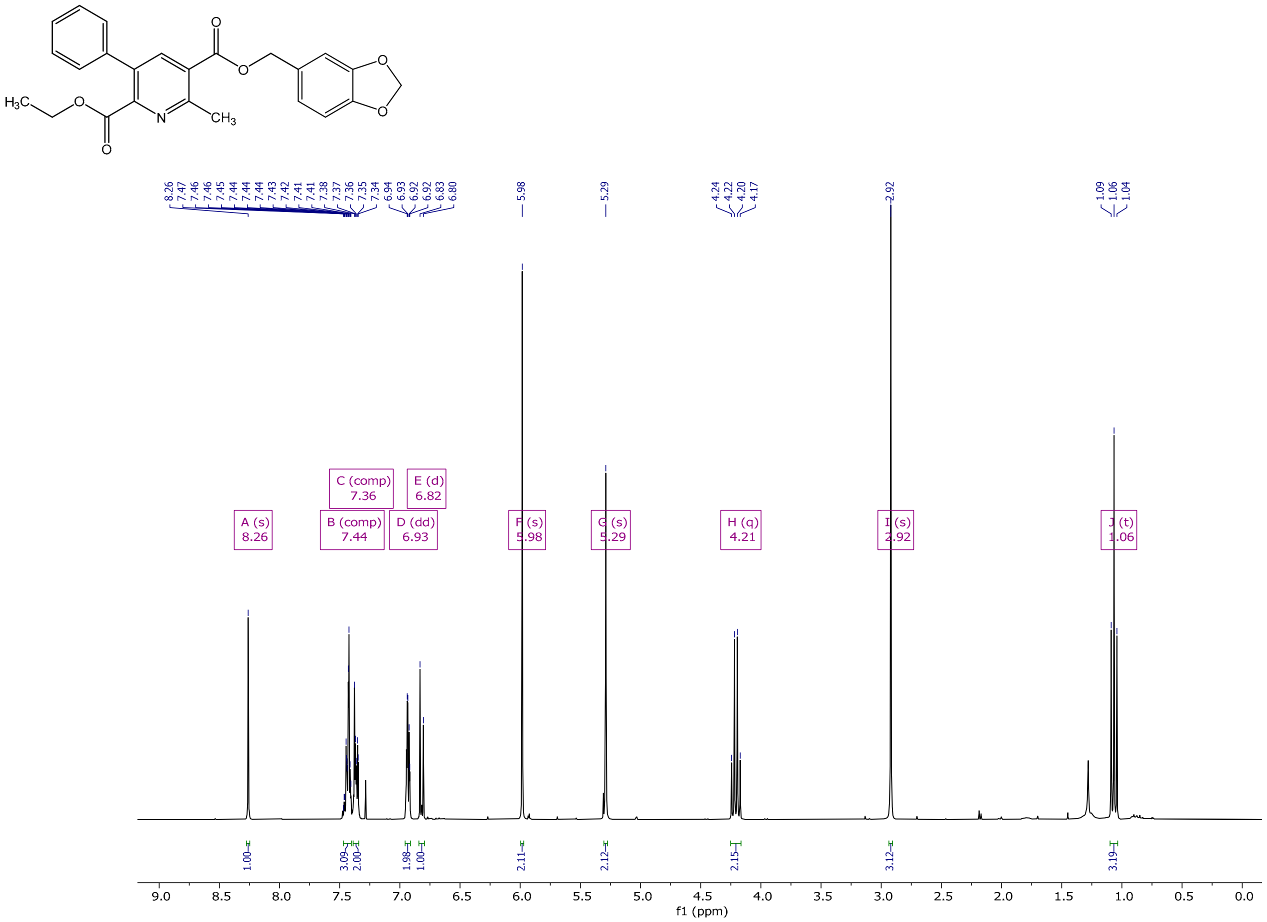

Figure S8. ^{13}C-NMR (75 MHz, $CDCl_3$) of compound **6a**

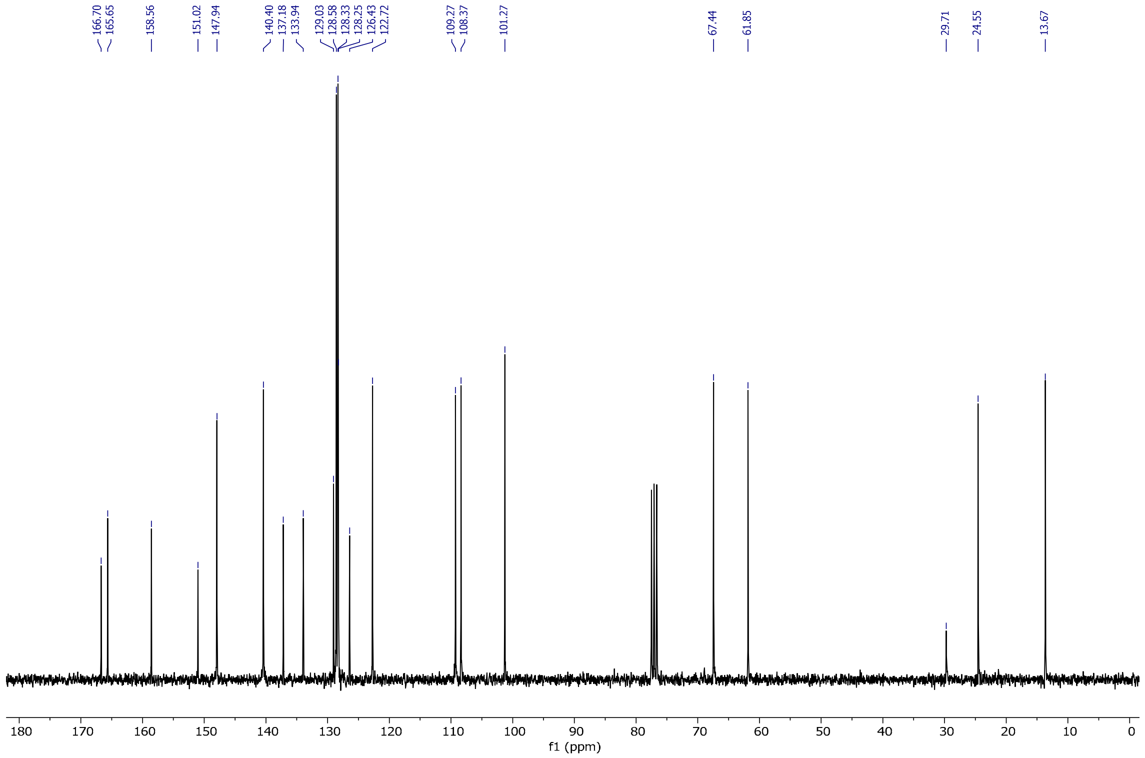
166.70
165.65
158.56
151.02
147.94
140.40
137.18
133.94
129.03
128.58
128.33
128.25
126.43
122.72
109.27
108.37
101.27
67.44
61.85
29.71
24.55
13.67
180 170 160 150 140 130 120 110 100 90 80 70 60 50 40 30 20 10 0
f1 (ppm)

Figure S9. ^{1}H-NMR (500 MHz, $CDCl_3$) of compound **7a**

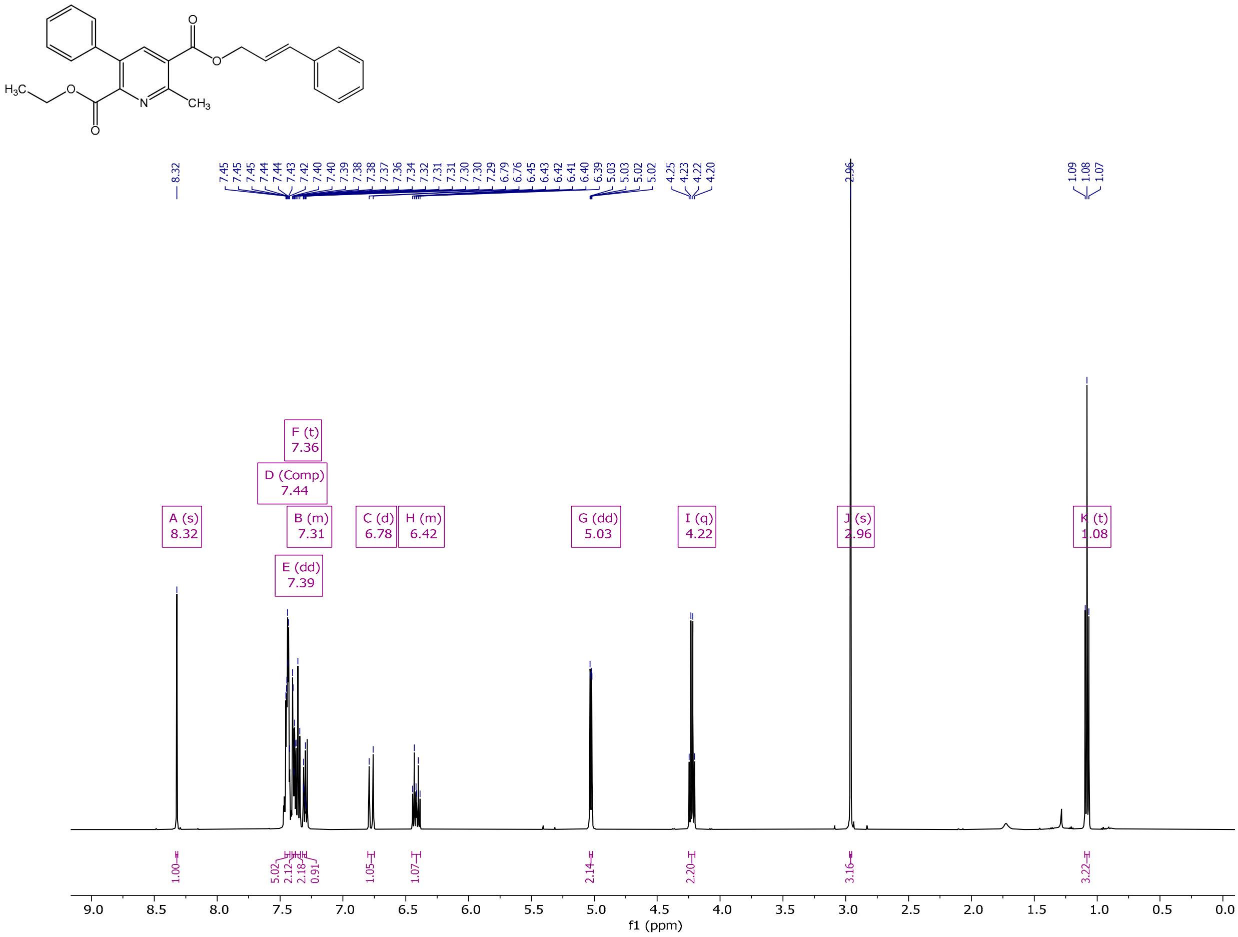

Figure S10. ^{13}C-NMR (126 MHz, $CDCl_3$) of compound **7a**

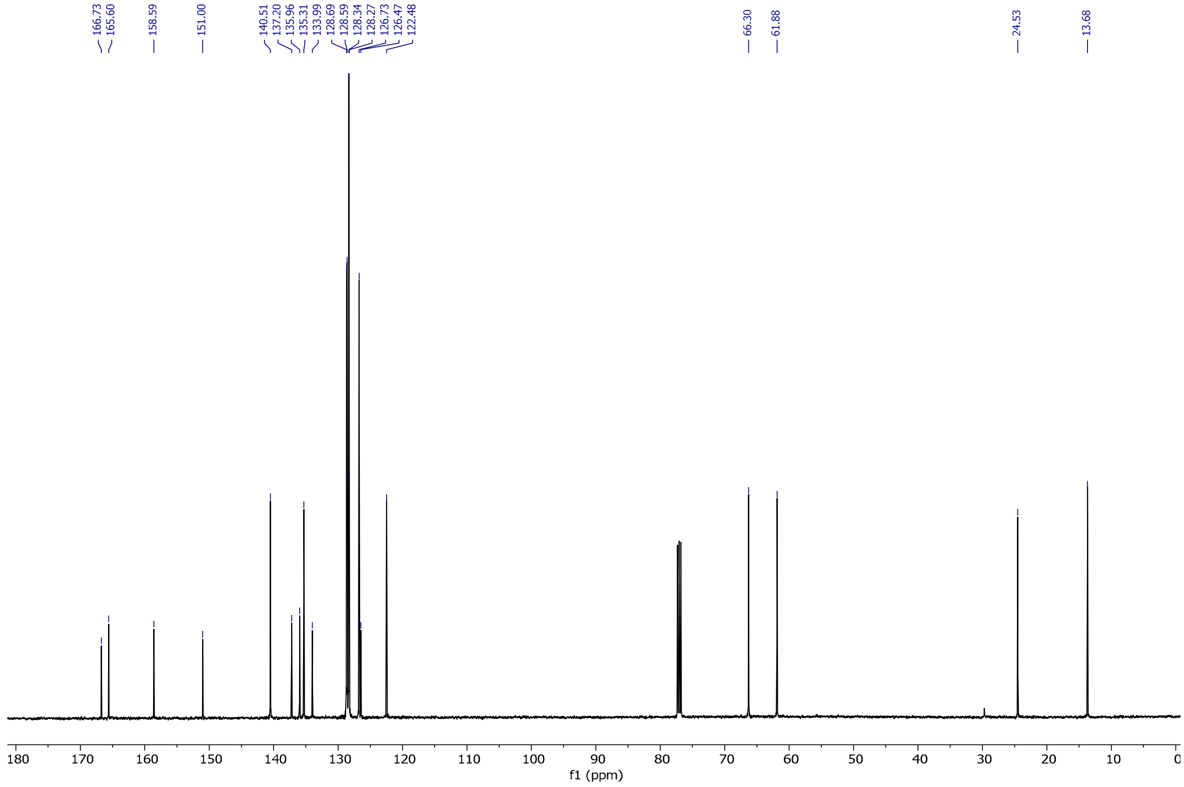

Figure S11. ^{1}H-NMR (500 MHz, $CDCl_3$) of compound **8a**

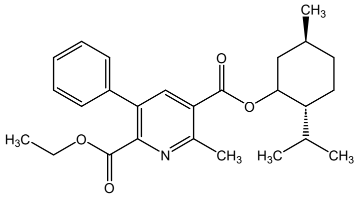

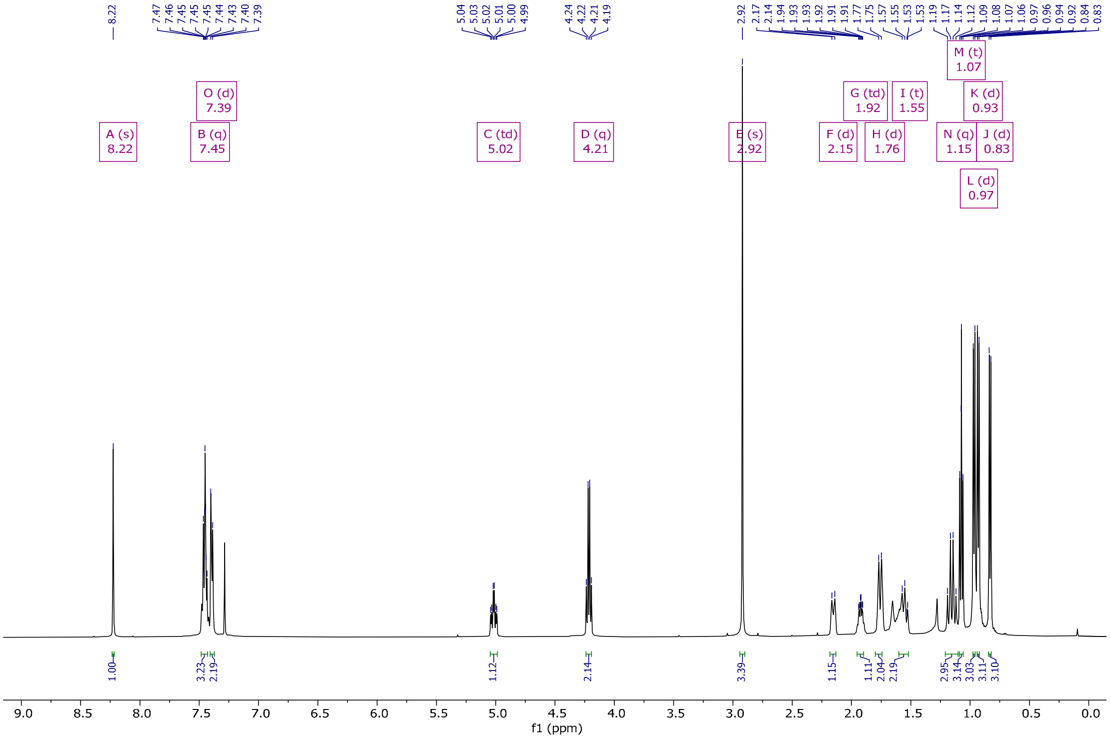

Figure S12. ^{13}C-NMR (126 MHz, $CDCl_3$) of compound **8a**

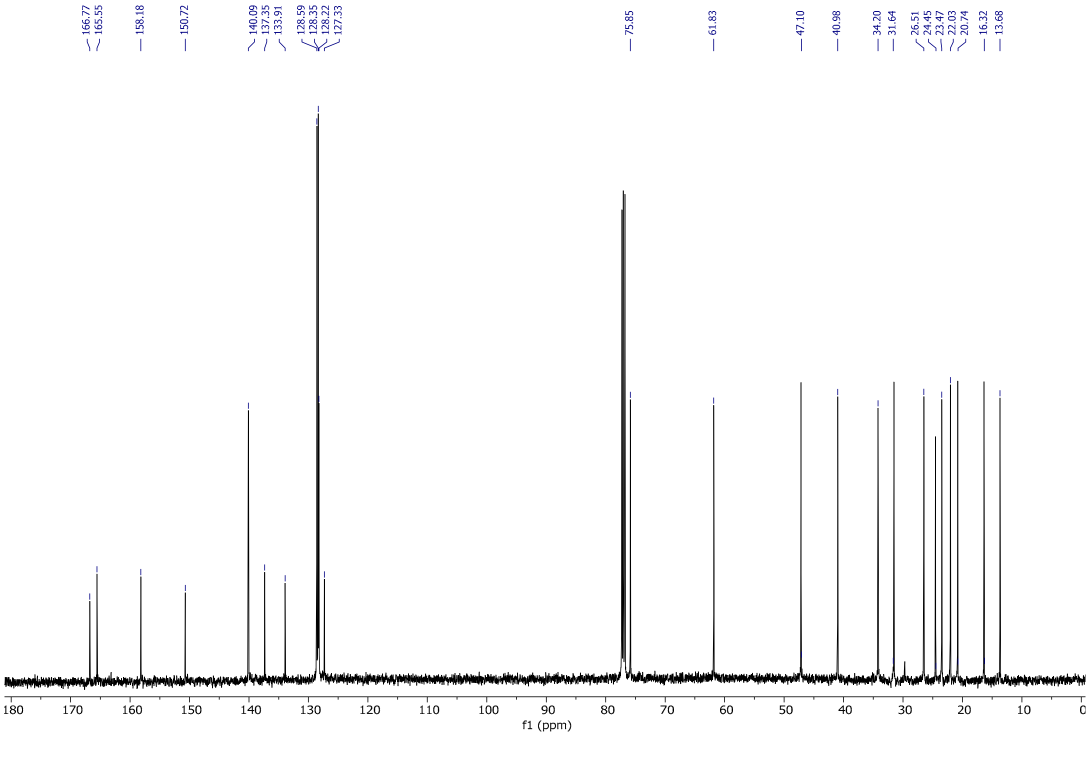

Figure S13. ^{1}H-NMR (500 MHz, $CDCl_3$) of compound **9a**

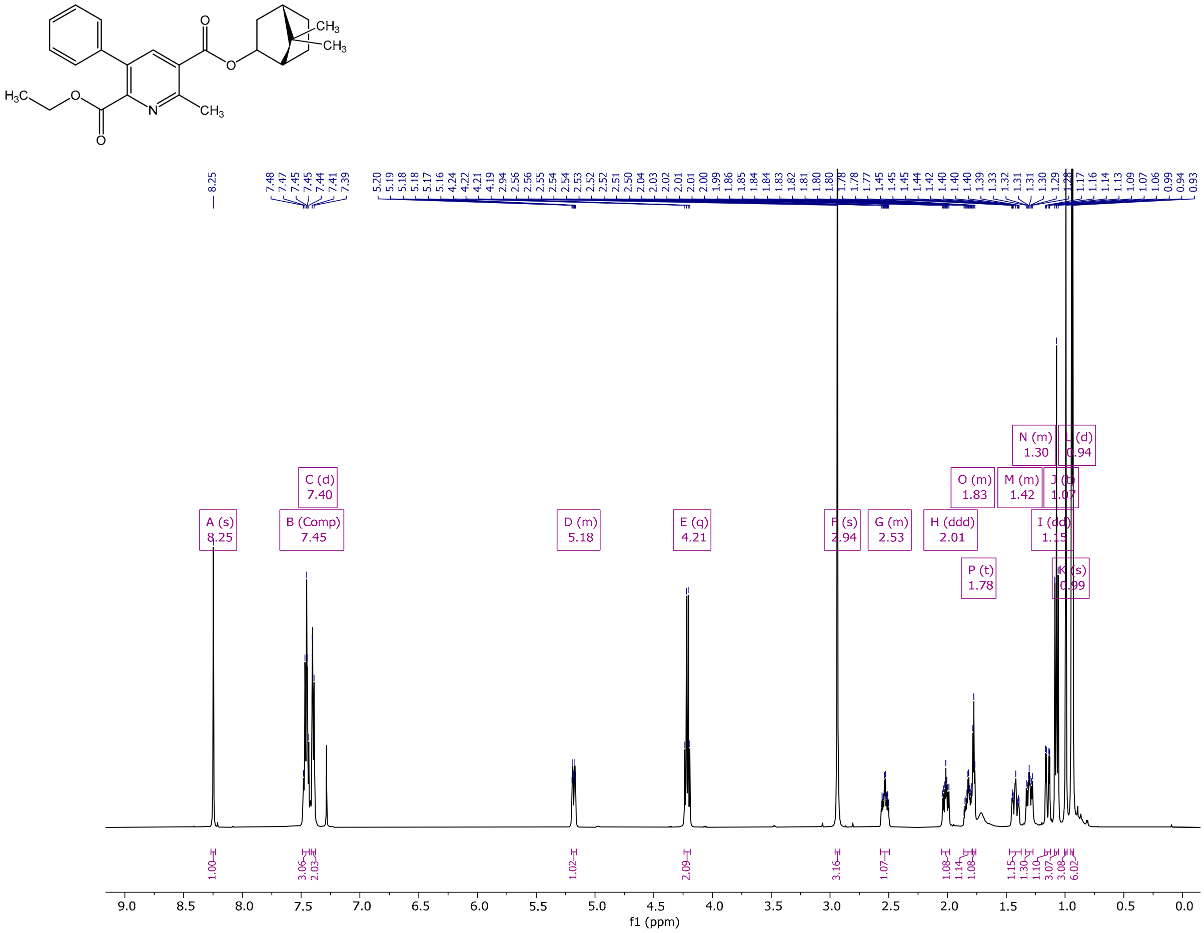

Figure S14. ^{13}C-NMR (126 MHz, $CDCl_3$) of compound **9a**

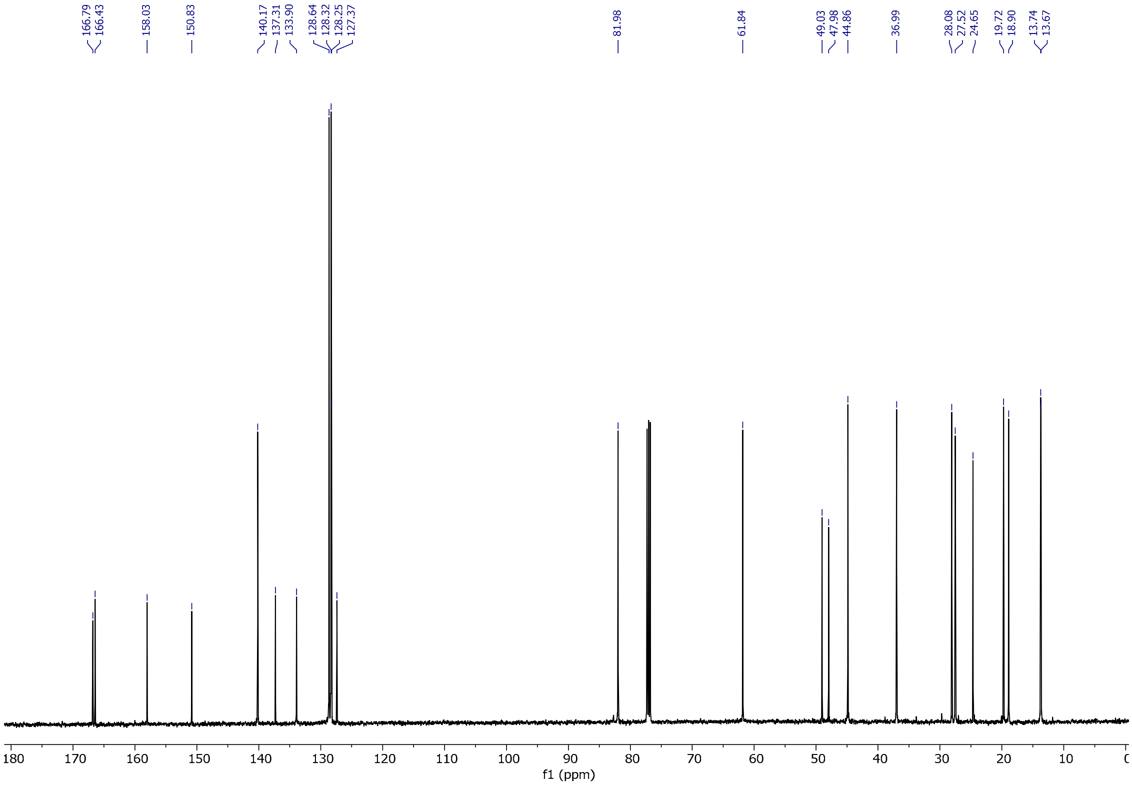

Figure S15. ^{1}H-NMR (500 MHz, $CDCl_3$) of compound **10a**

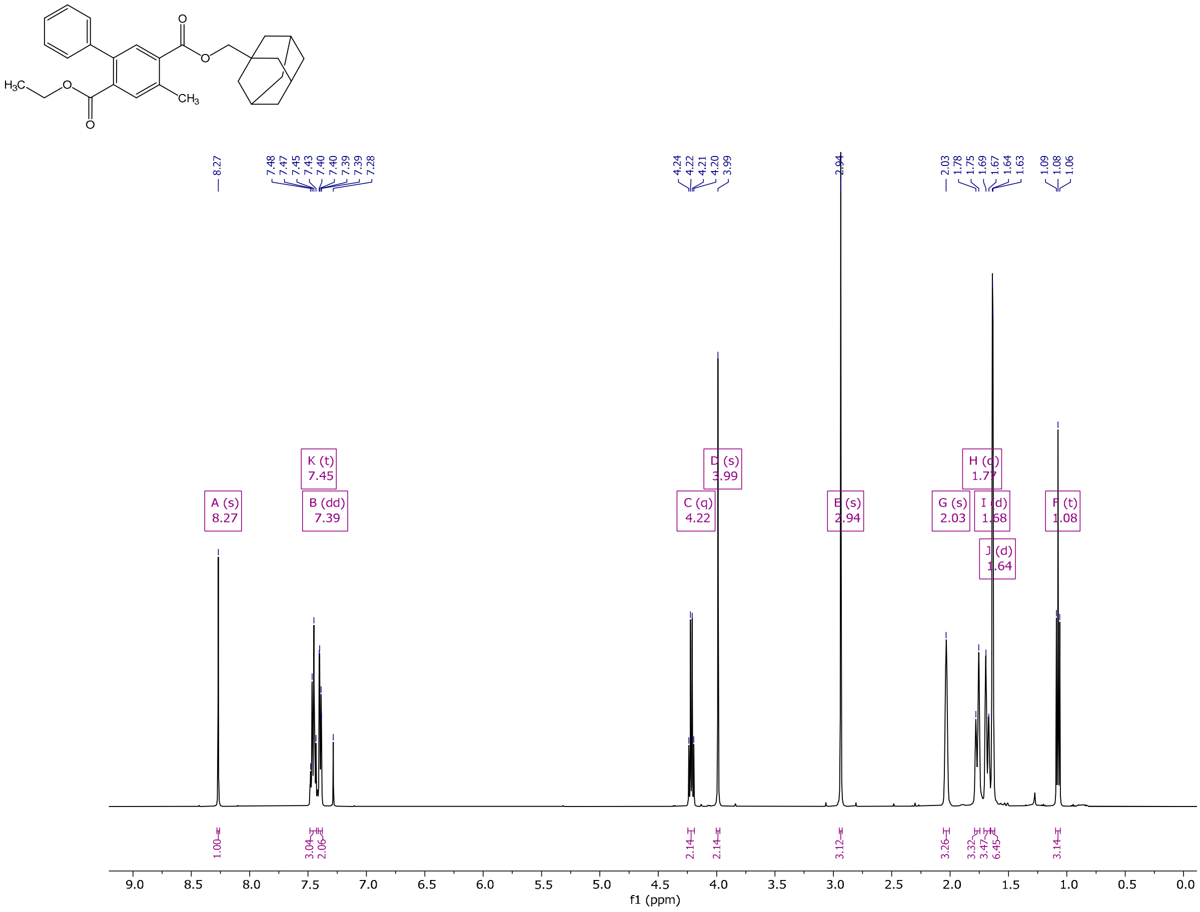

Figure S16. ^{13}C-NMR (126 MHz, $CDCl_3$) of compound **10a**

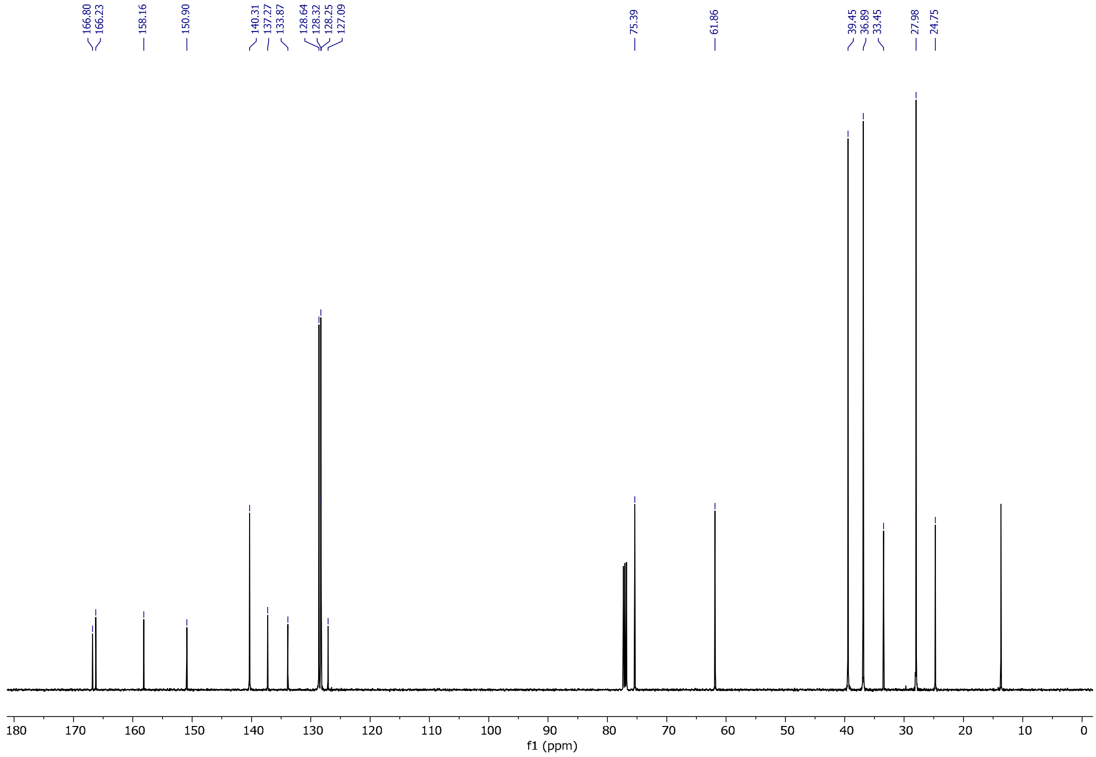

Figure S17. ^{1}H-NMR (500 MHz, $CDCl_3$) of compound **11a**

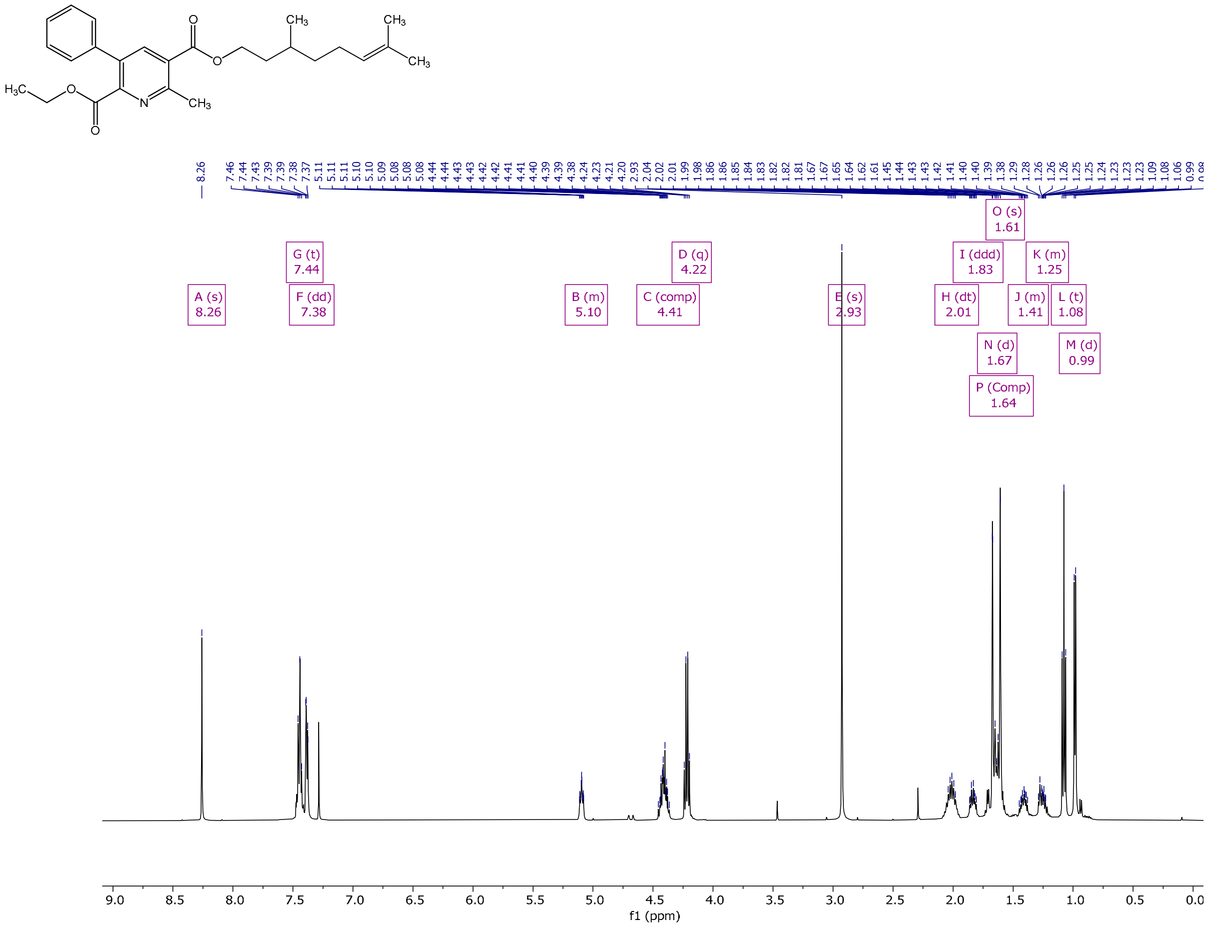

Figure S18. ^{13}C-NMR (126 MHz, $CDCl_3$) of compound **11a**

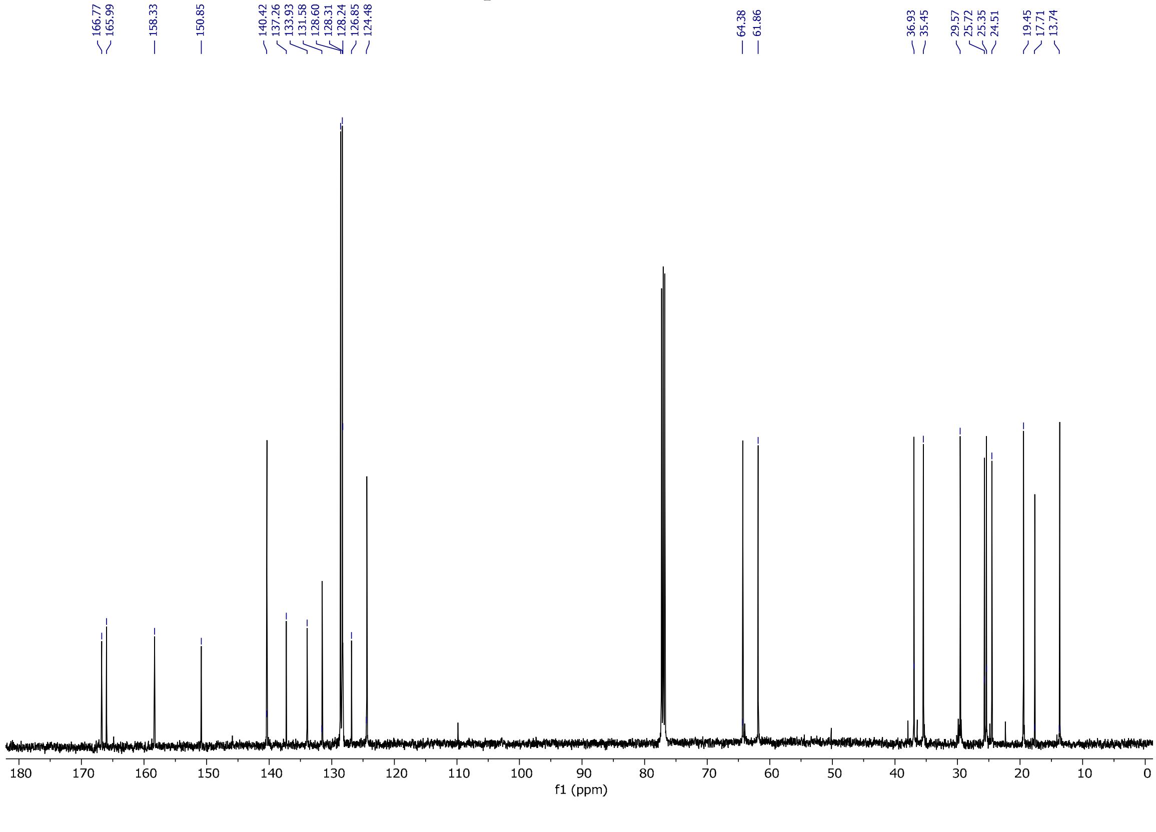

Figure S19. ^{1}H-NMR (500 MHz, $CDCl_3$) of compound **12a**

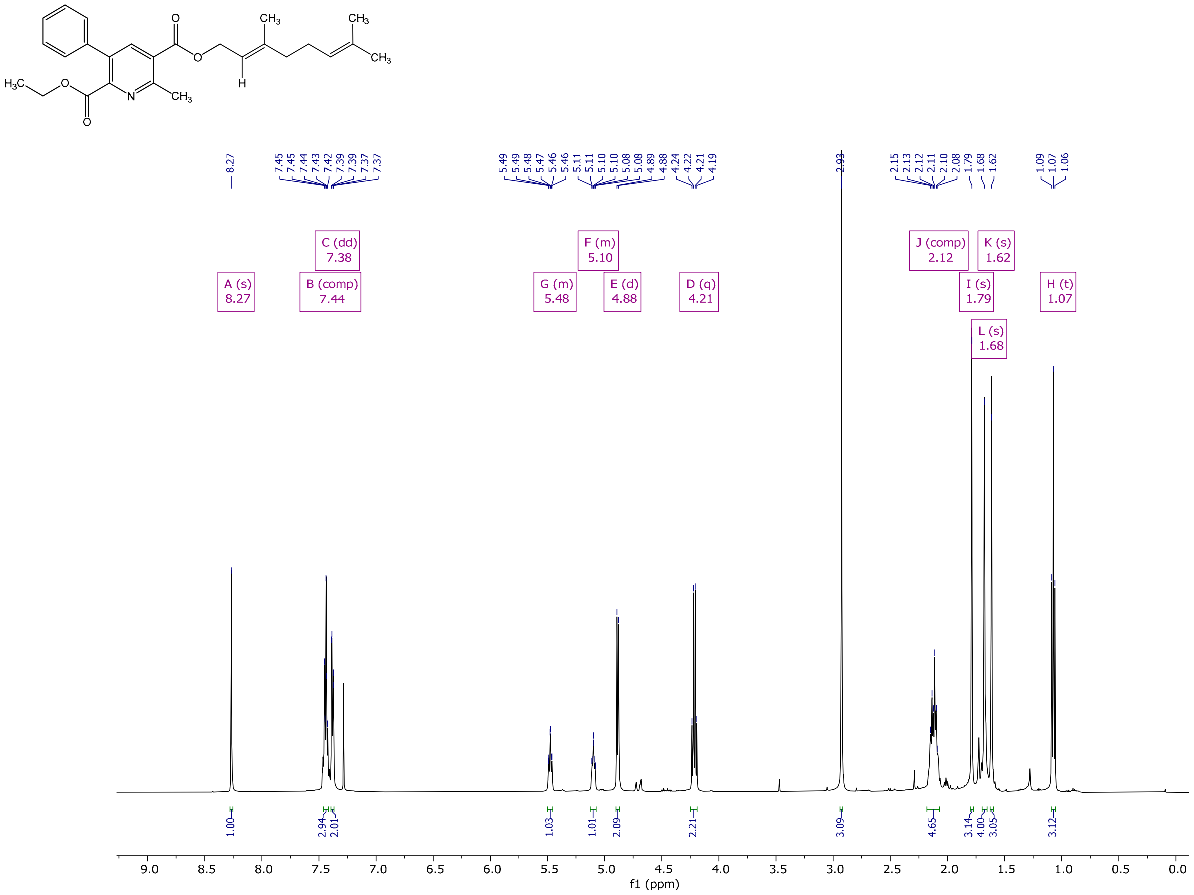

Figure S20. ^{13}C-NMR (126 MHz, $CDCl_3$) of compound **12a**

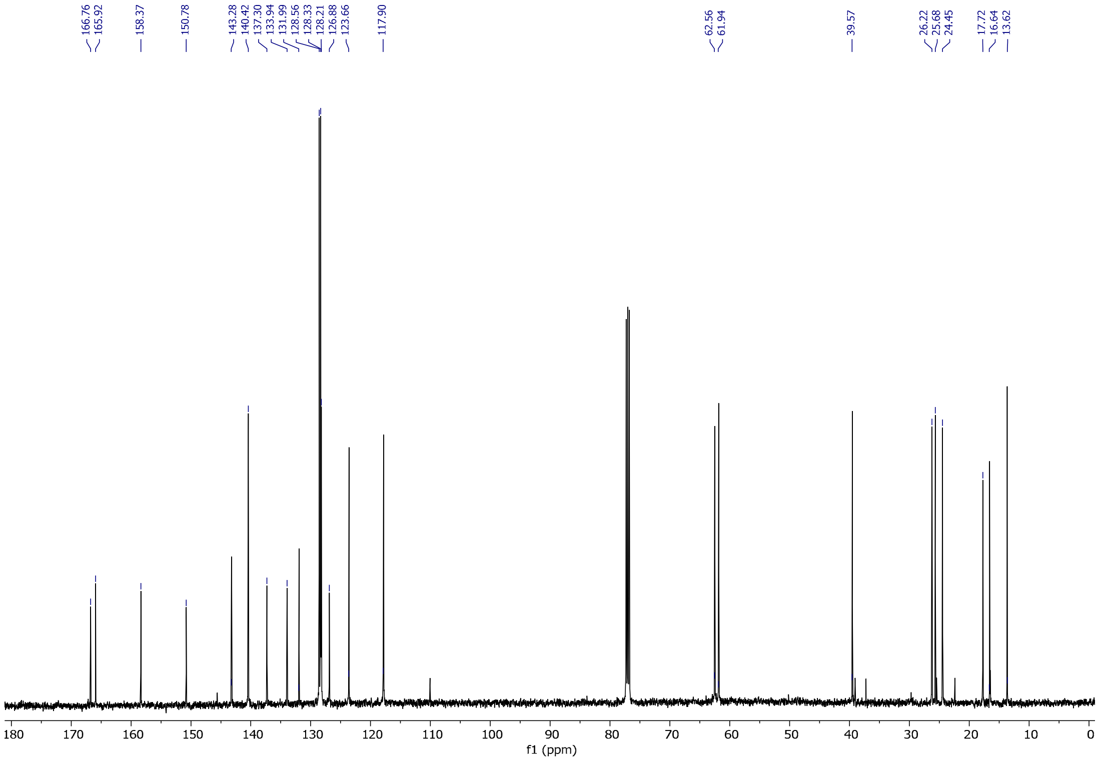

Figure S21. ^{1}H-NMR (500 MHz, $CDCl_3$) of compound **3b**

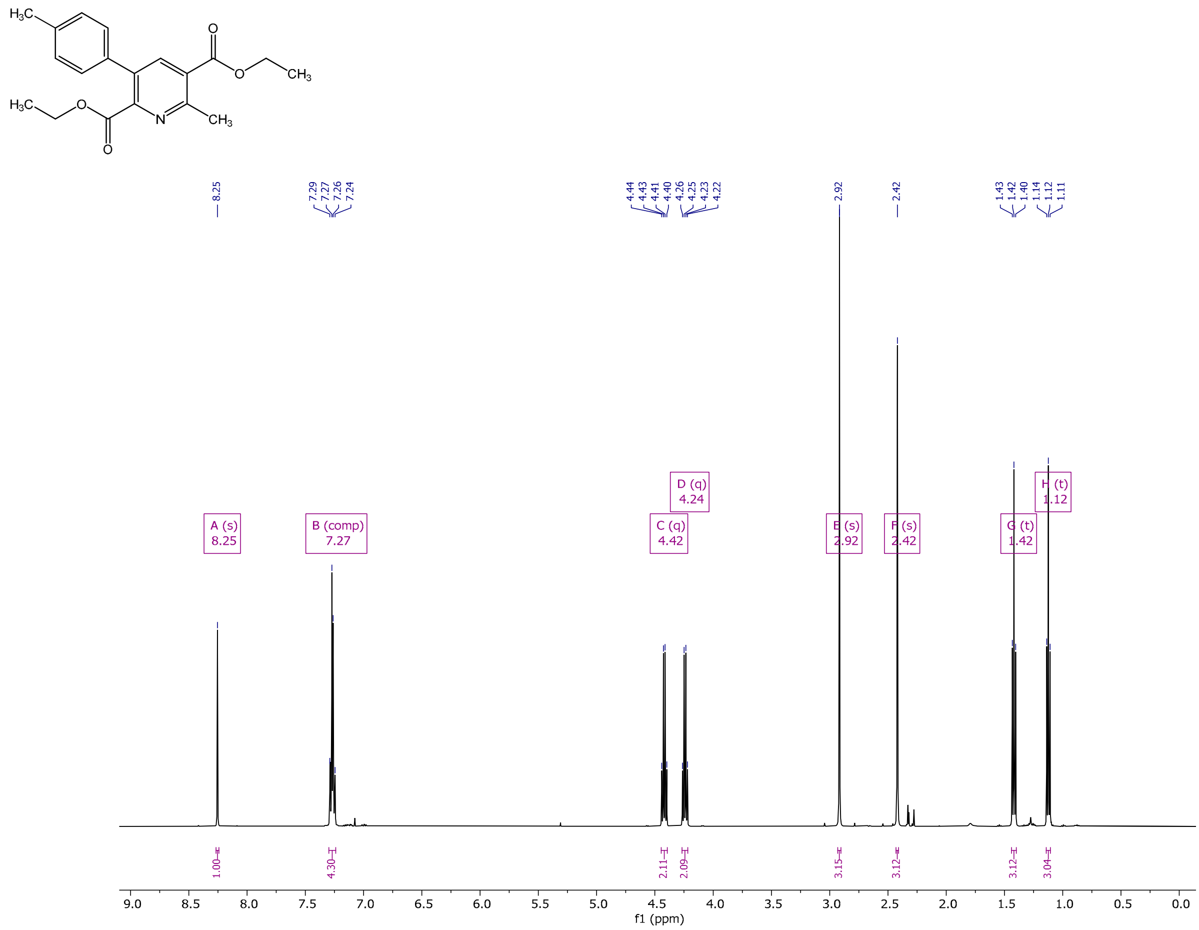

Figure S22. ^{13}C-NMR (126 MHz, $CDCl_3$) of compound **3b**

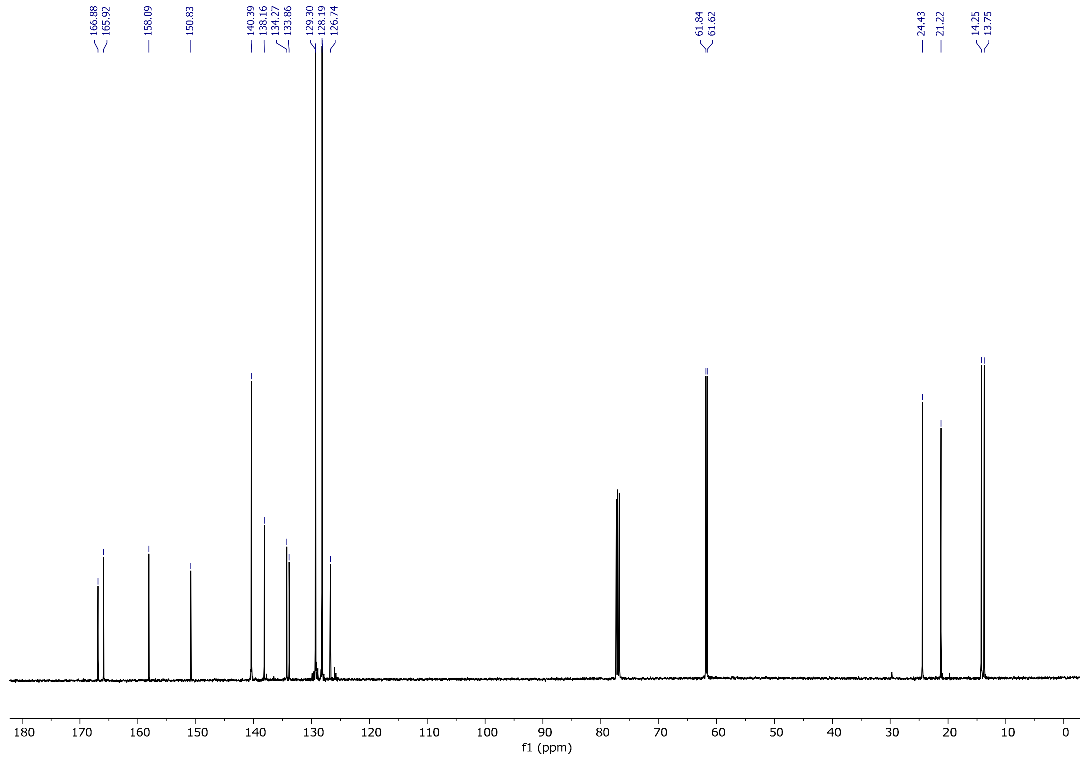

Figure S23. ^{1}H-NMR (500 MHz, $CDCl_3$) of compound **4b**

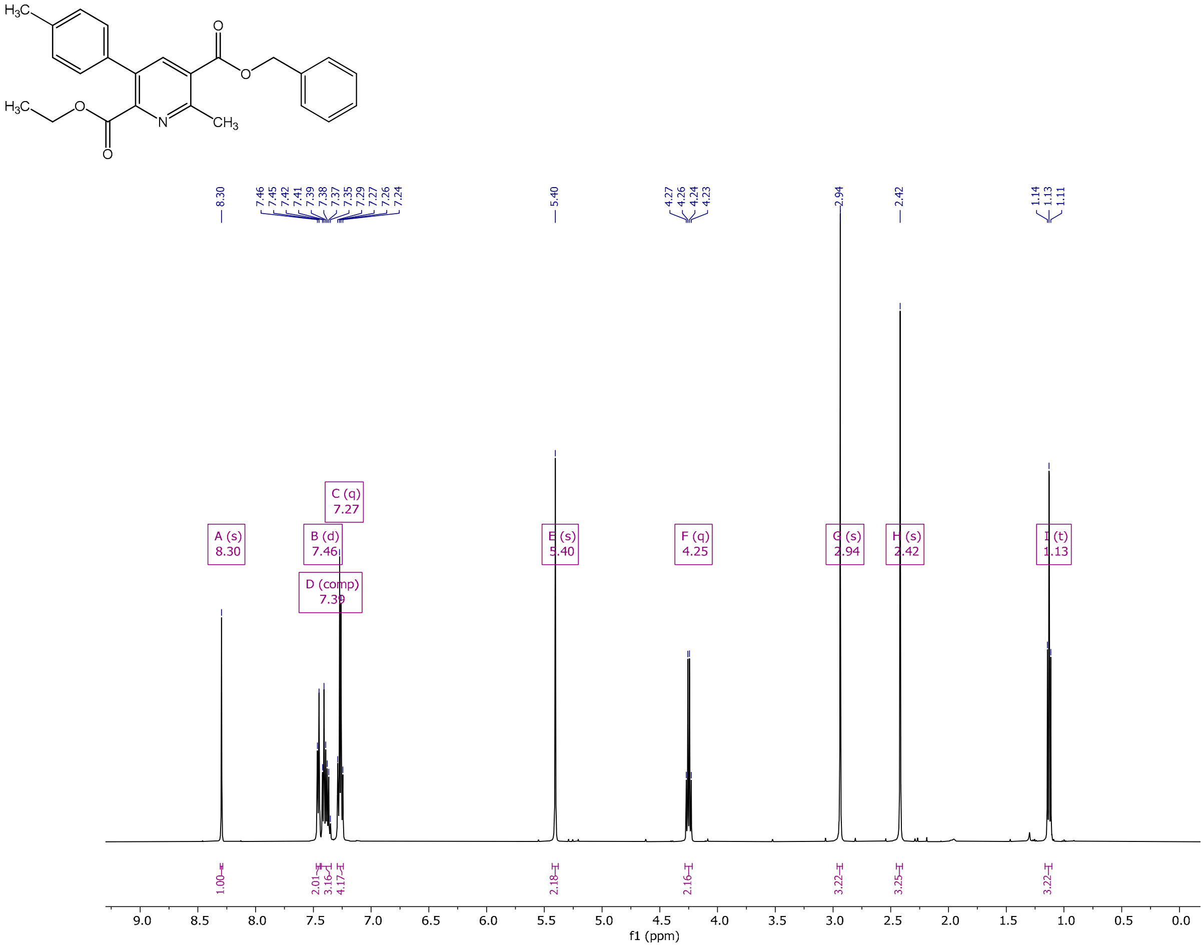

Figure S24. ^{13}C-NMR (126 MHz, $CDCl_3$) of compound **4b**

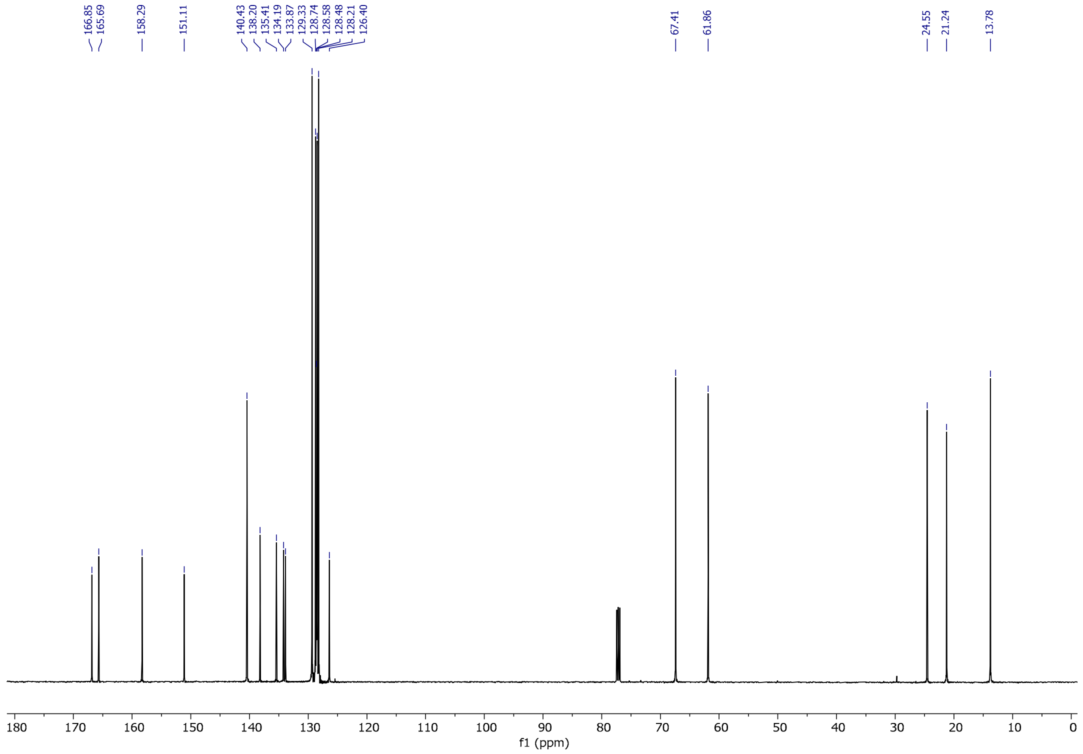

Figure S25. ^{1}H NMR (300 MHz, $CDCl_3$) of compound **5b**

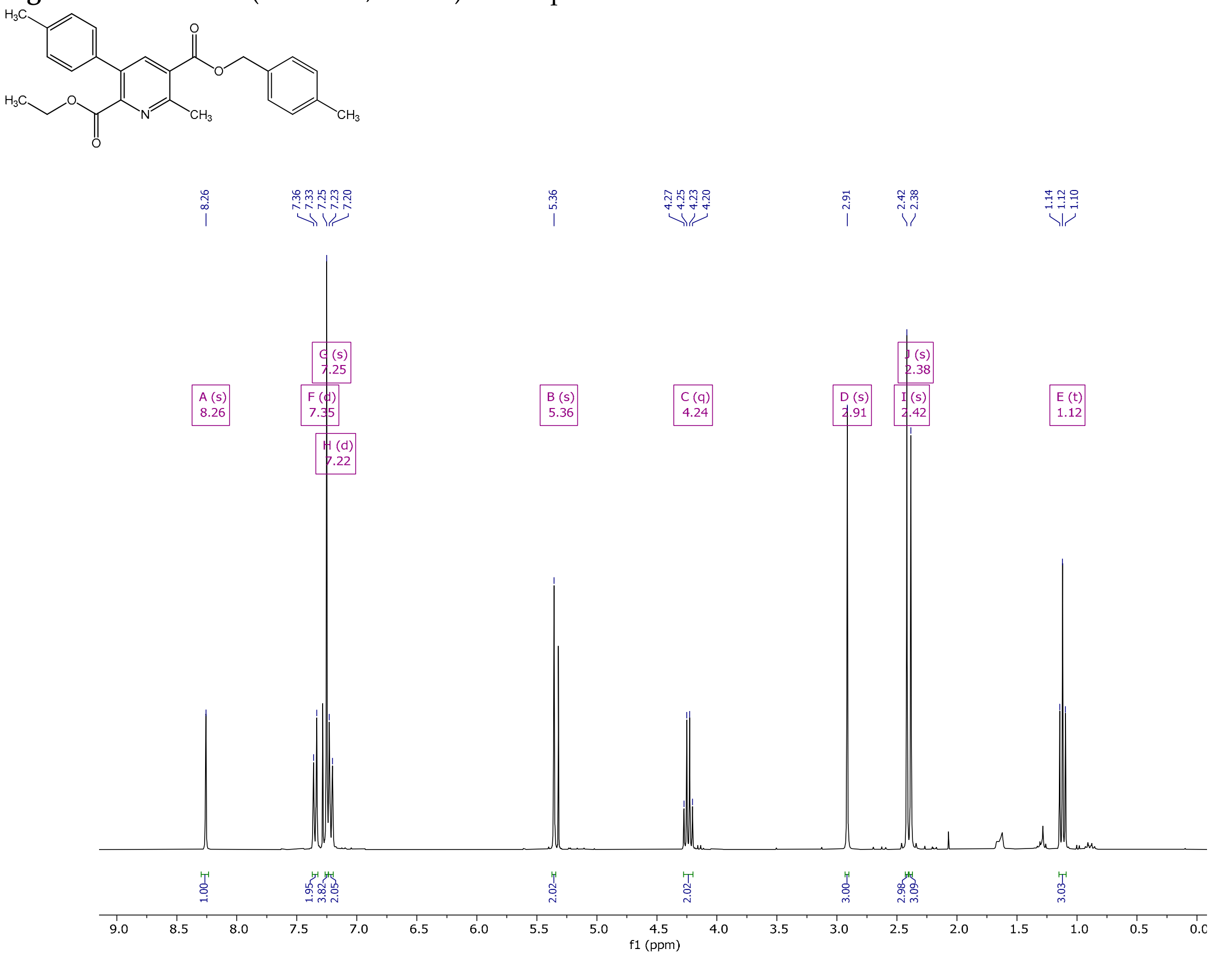

Figure S26. ^{13}C NMR (75 MHz, $CDCl_3$) of compound **5b**

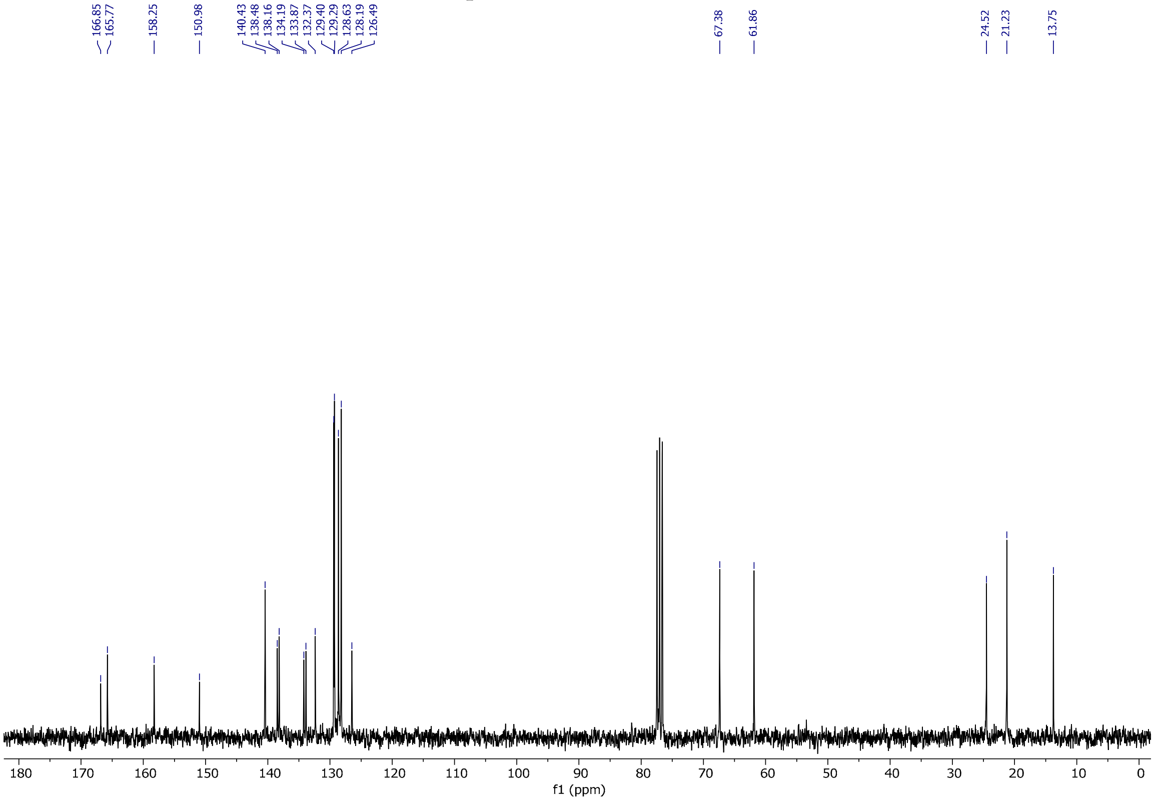

Figure S27. ^{1}H-NMR (500 MHz, $CDCl_3$) of compound **6b**

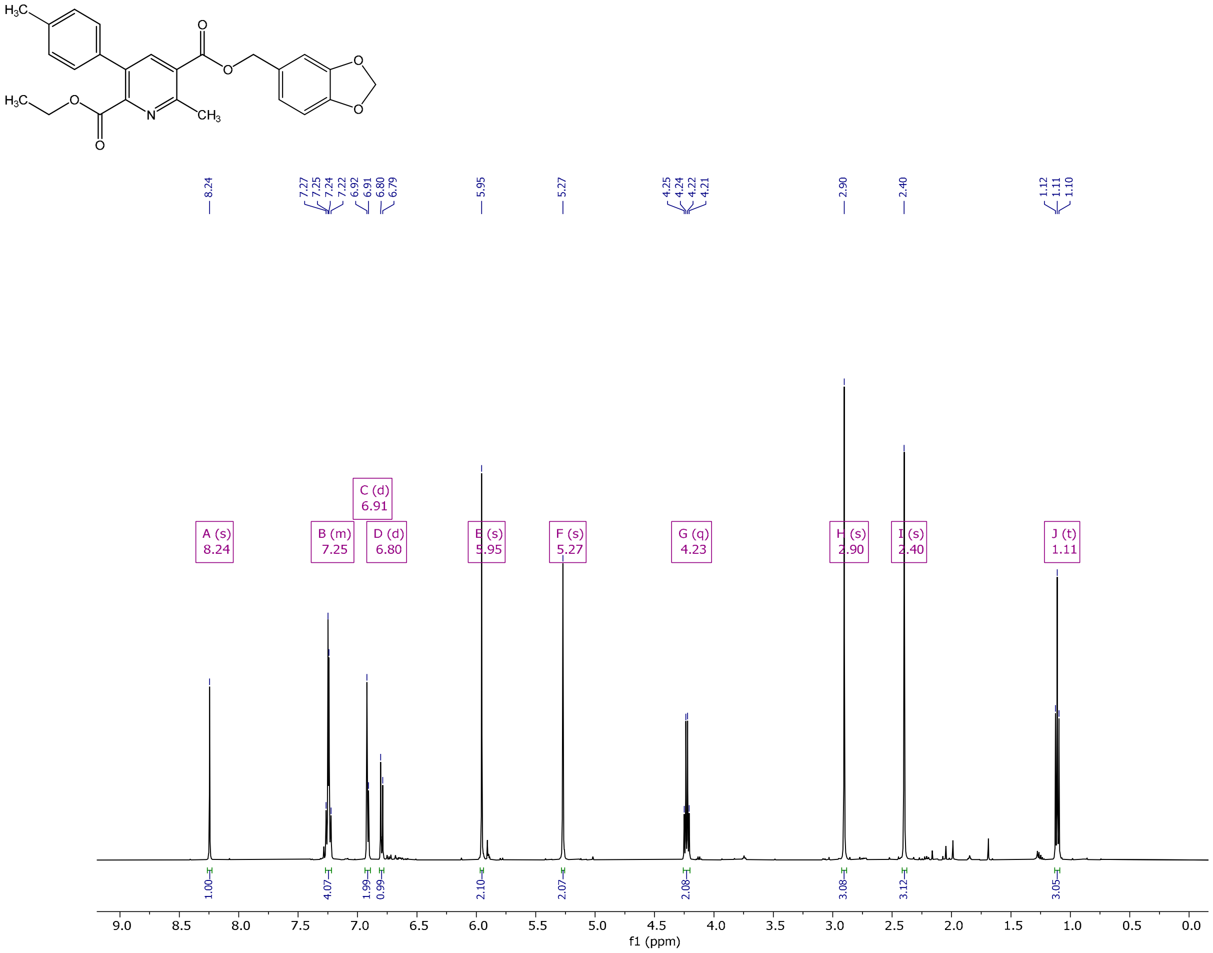

Figure S28. ^{13}C-NMR (126 MHz, $CDCl_3$) of compound **6b**

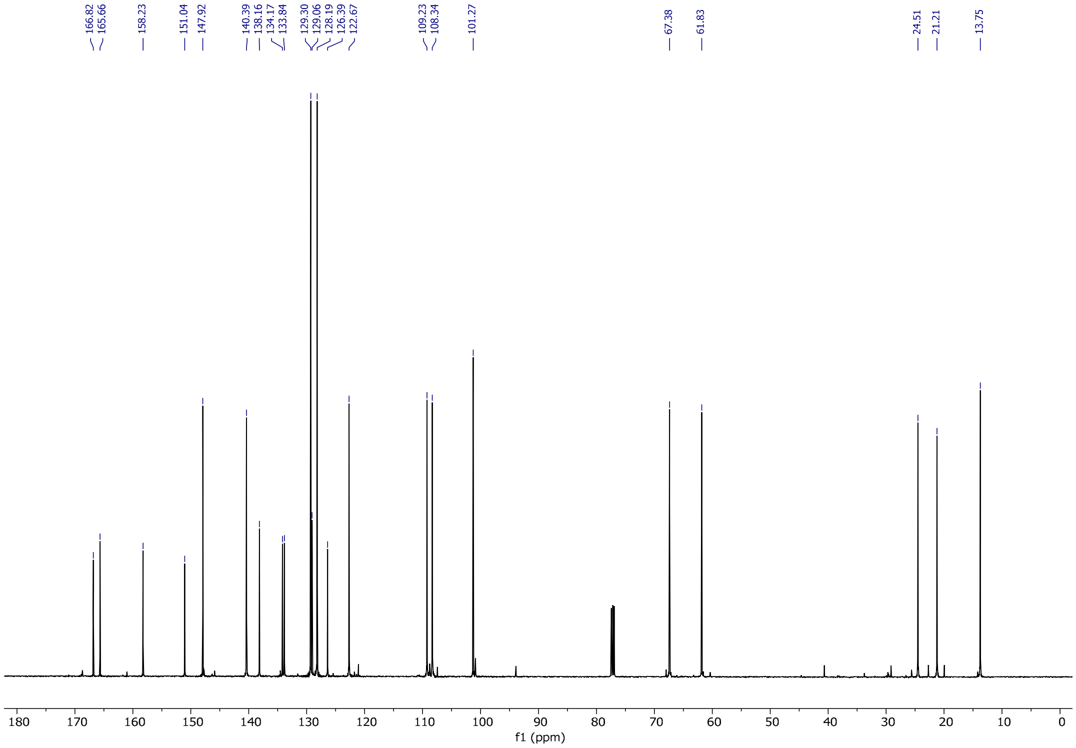

Figure S29. ^{1}H-NMR (500 MHz, $CDCl_3$) of compound **7b**

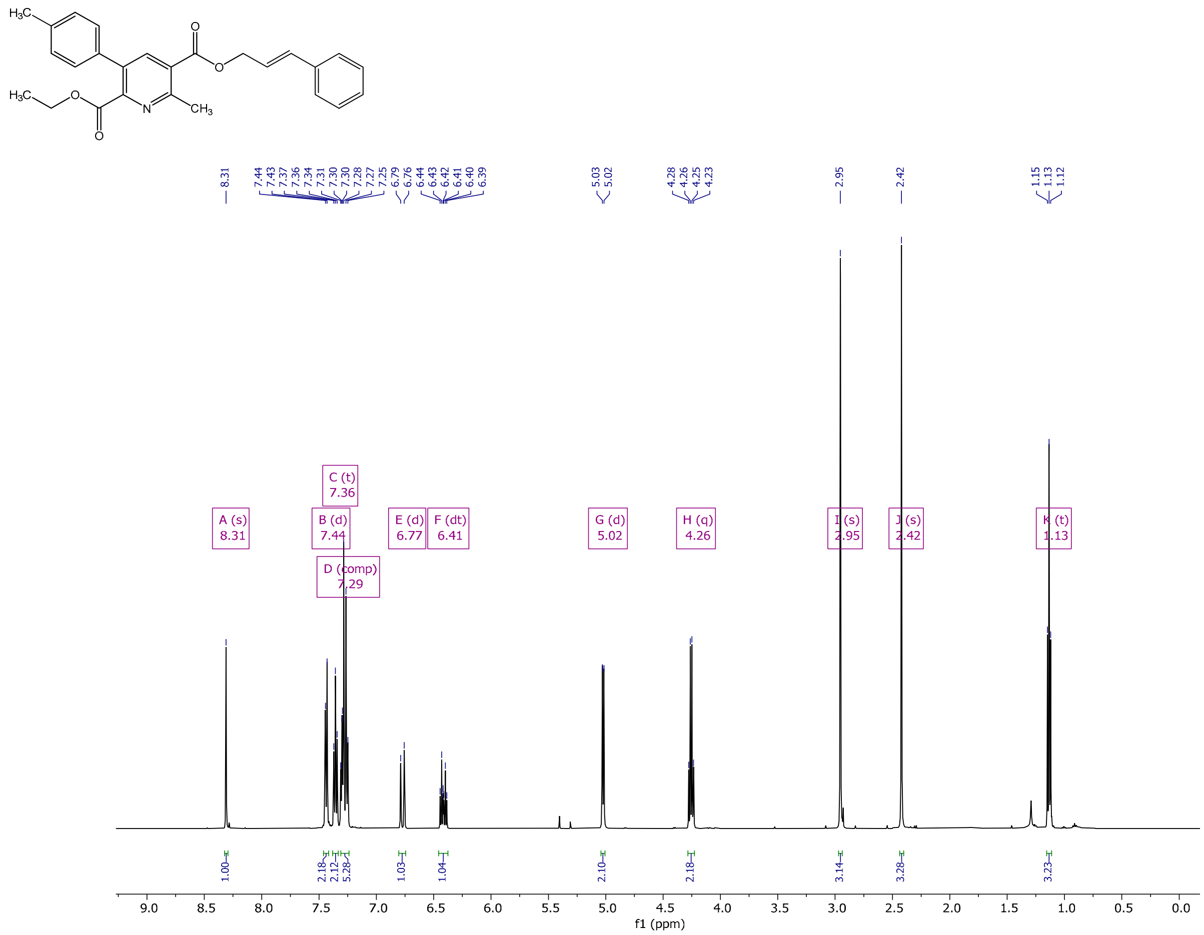

Figure S30. ^{13}C-NMR (126 MHz, $CDCl_3$) of compound **7b**

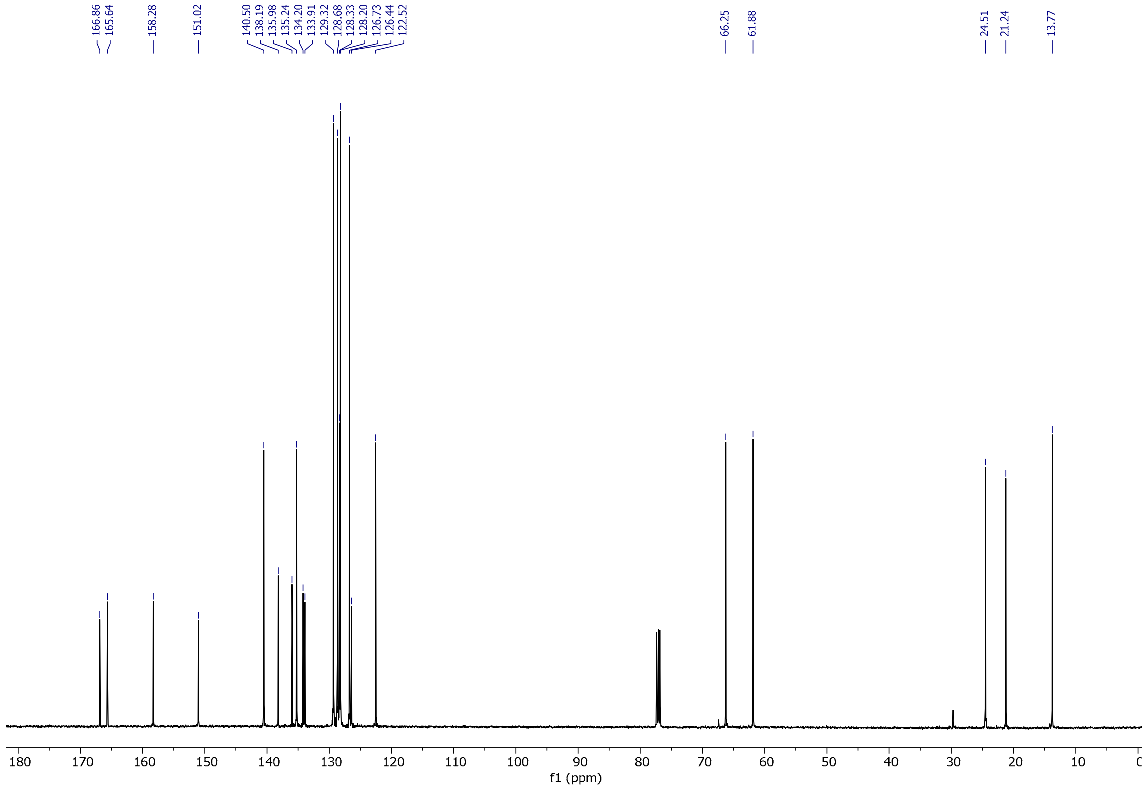

Figure S31. ^{1}H NMR (300 MHz, $CDCl_3$) of compound **8b**

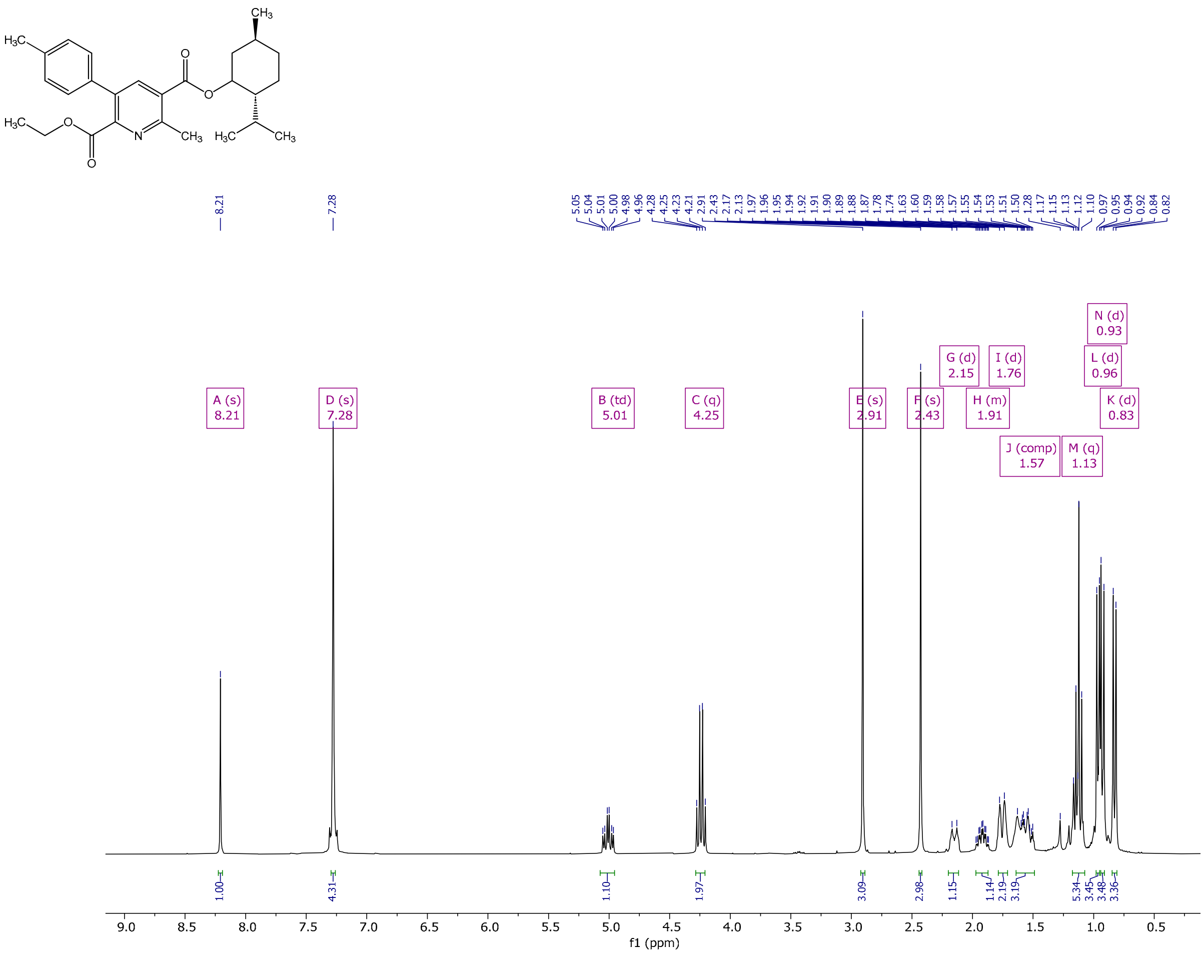

Figure S32. ^{13}C NMR (75 MHz, $CDCl_3$) of compound **8b**

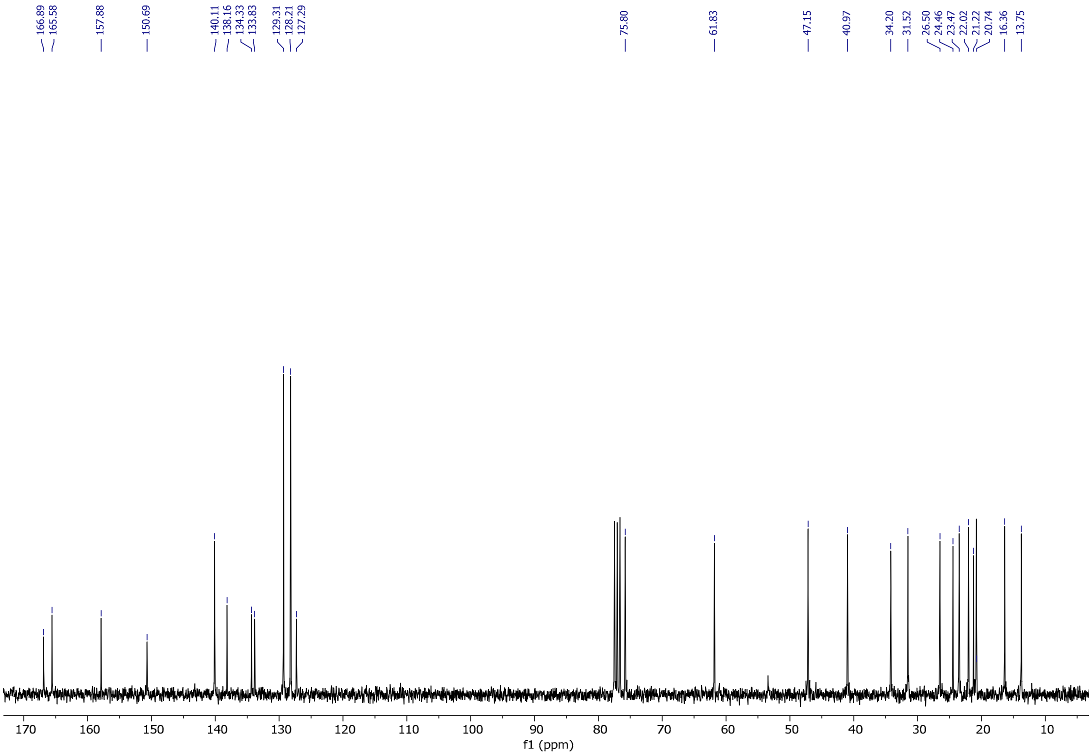

Figure S33. ^{1}H-NMR (500 MHz, $CDCl_3$) of compound **9b**

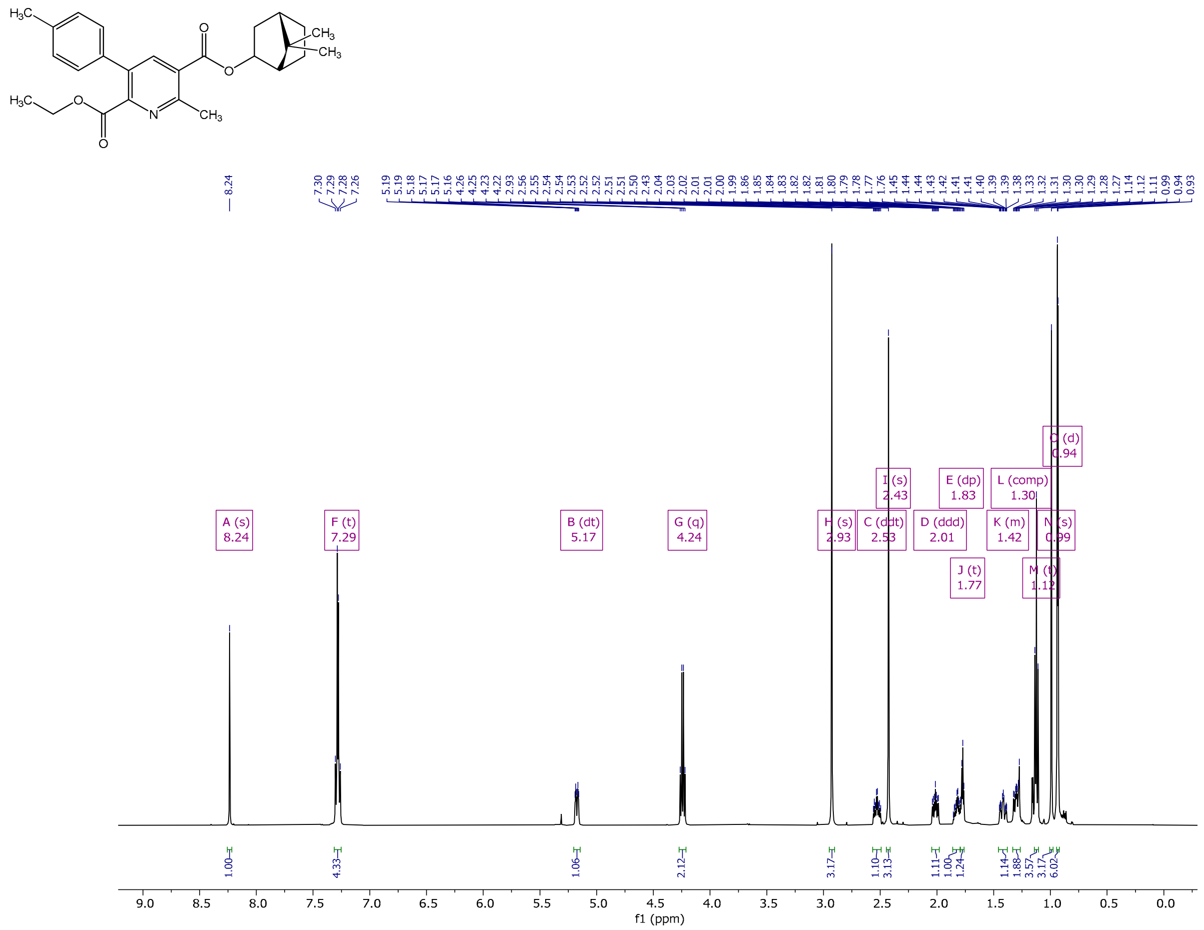

Figure S34. ^{13}C-NMR (126 MHz, $CDCl_3$) of compound **9b**

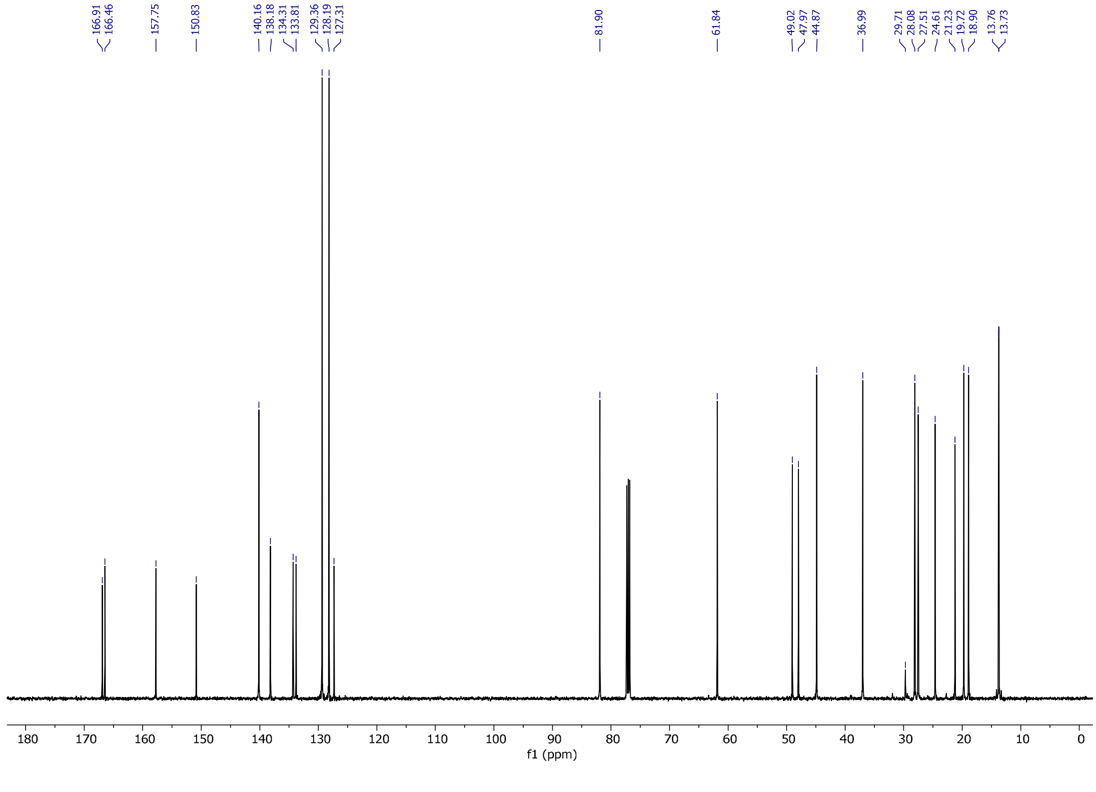

Figure S35. ^{1}H-NMR (500 MHz, $CDCl_3$) of compound **10b**

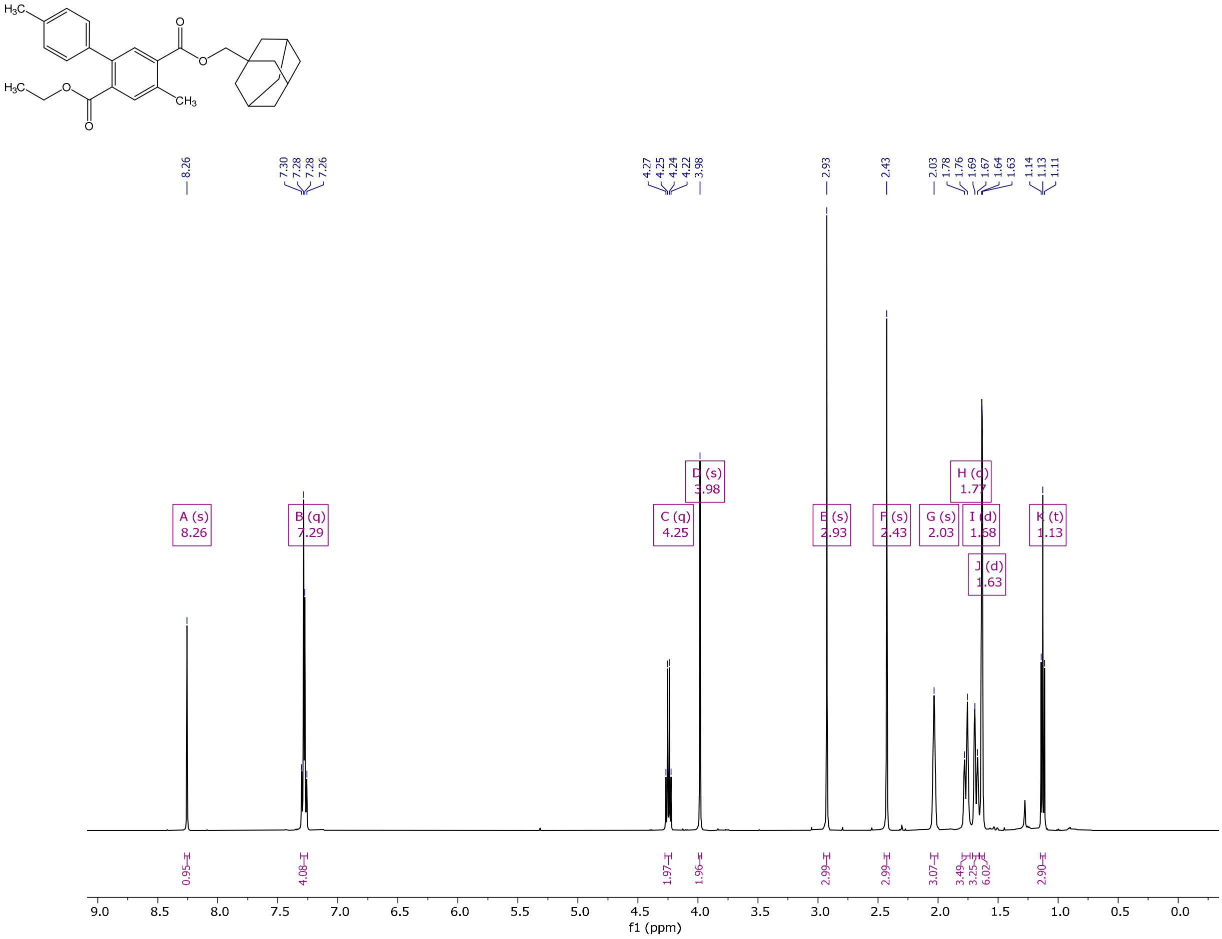

Figure S36. ^{13}C-NMR (126 MHz, $CDCl_3$) of compound **10b**

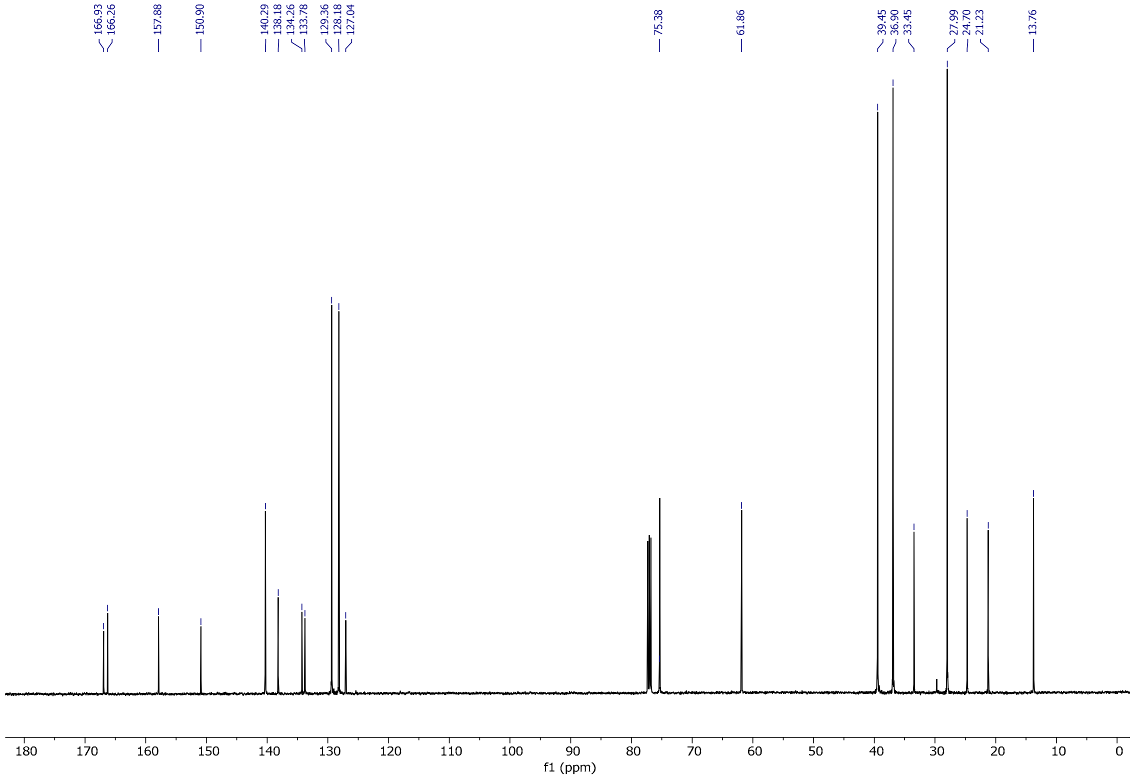

Figure S37. ^{1}H NMR (300 MHz, $CDCl_3$) of compound **11b**

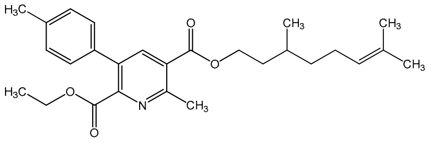

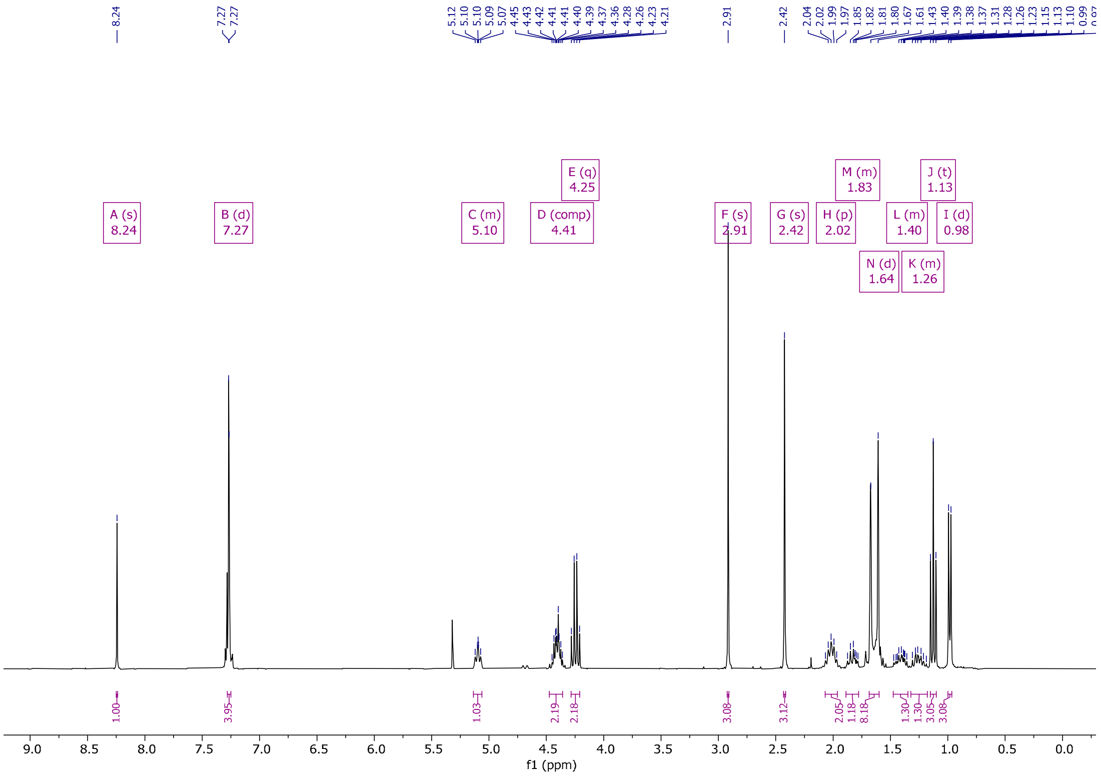

Figure S38. ^{13}C NMR (75 MHz, $CDCl_3$) of compound **11b**

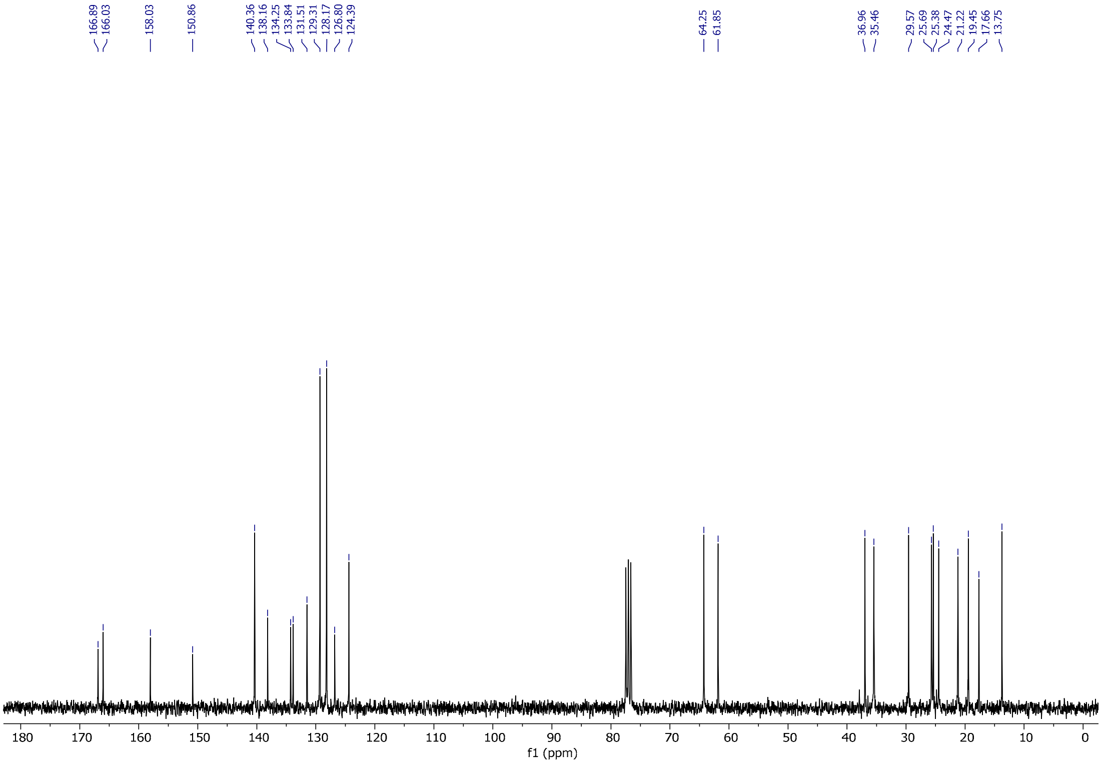

Figure S39. ^{1}H NMR (300 MHz, $CDCl_3$) of compound **12b**

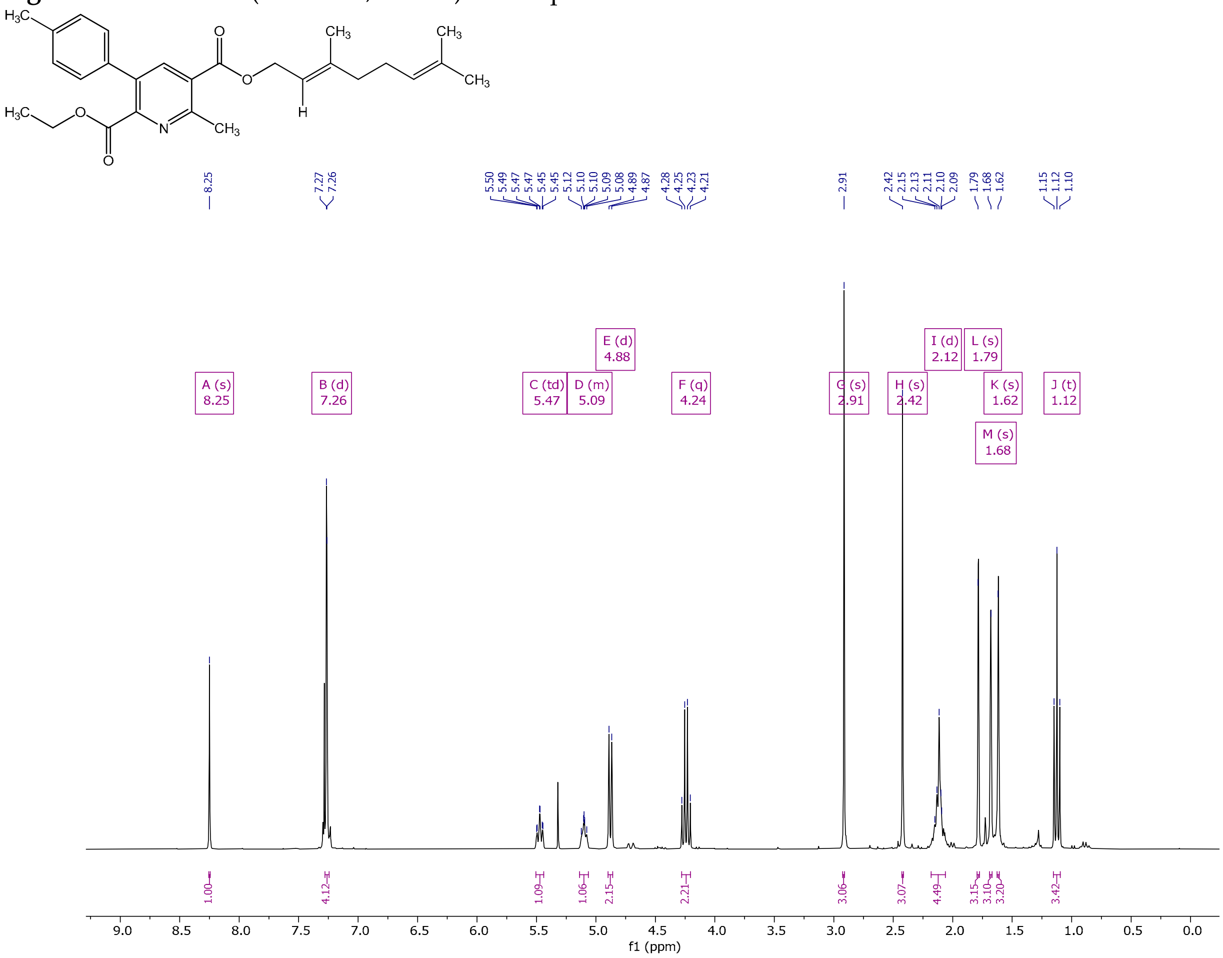

Figure S40. ^{13}C NMR (75 MHz, $CDCl_3$) of compound **12b**

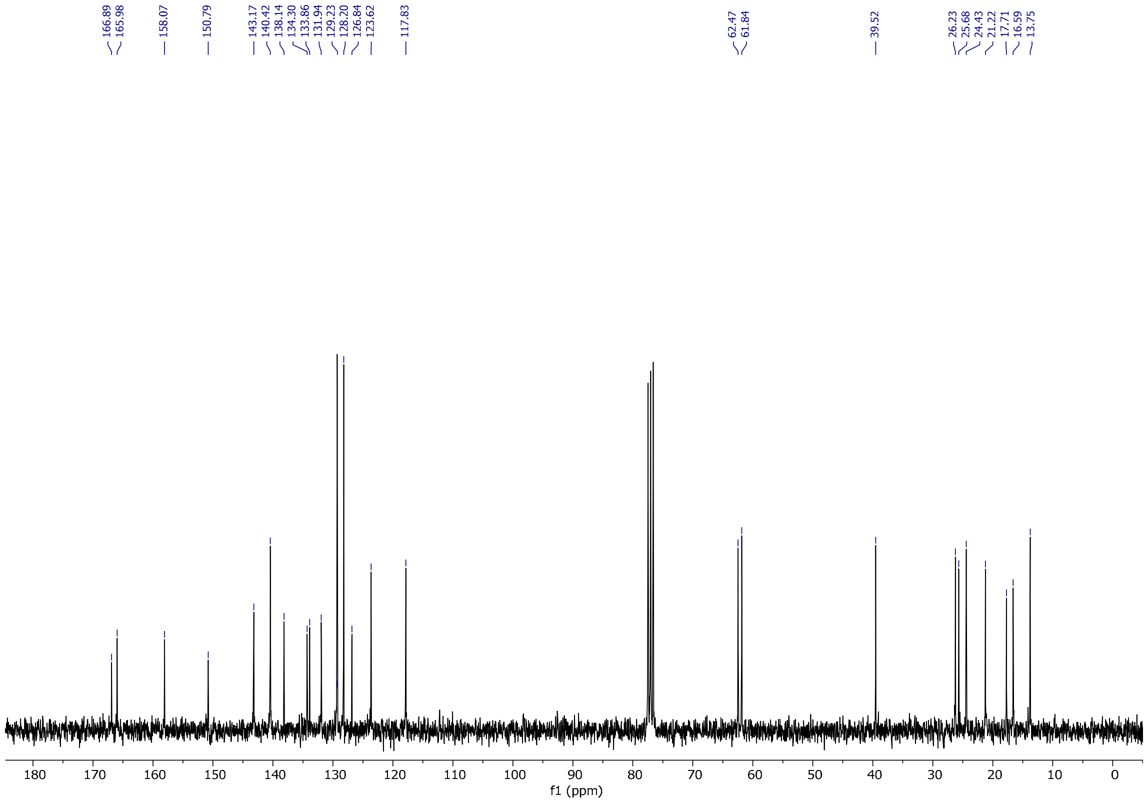

Figure S41. ^{1}H-NMR (500 MHz, $CDCl_3$) of compound **3c**

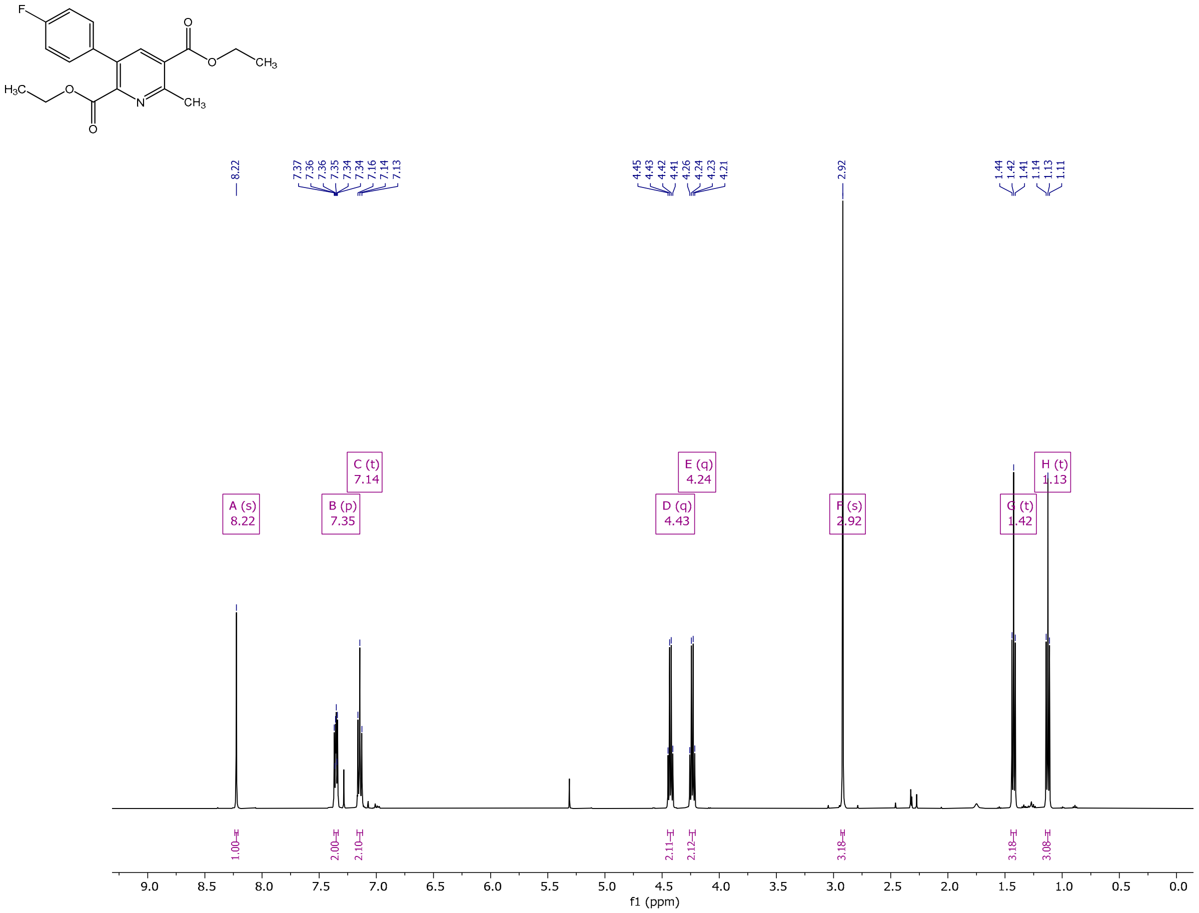

Figure S42. ^{13}C-NMR (126 MHz, $CDCl_3$) of compound **3c**

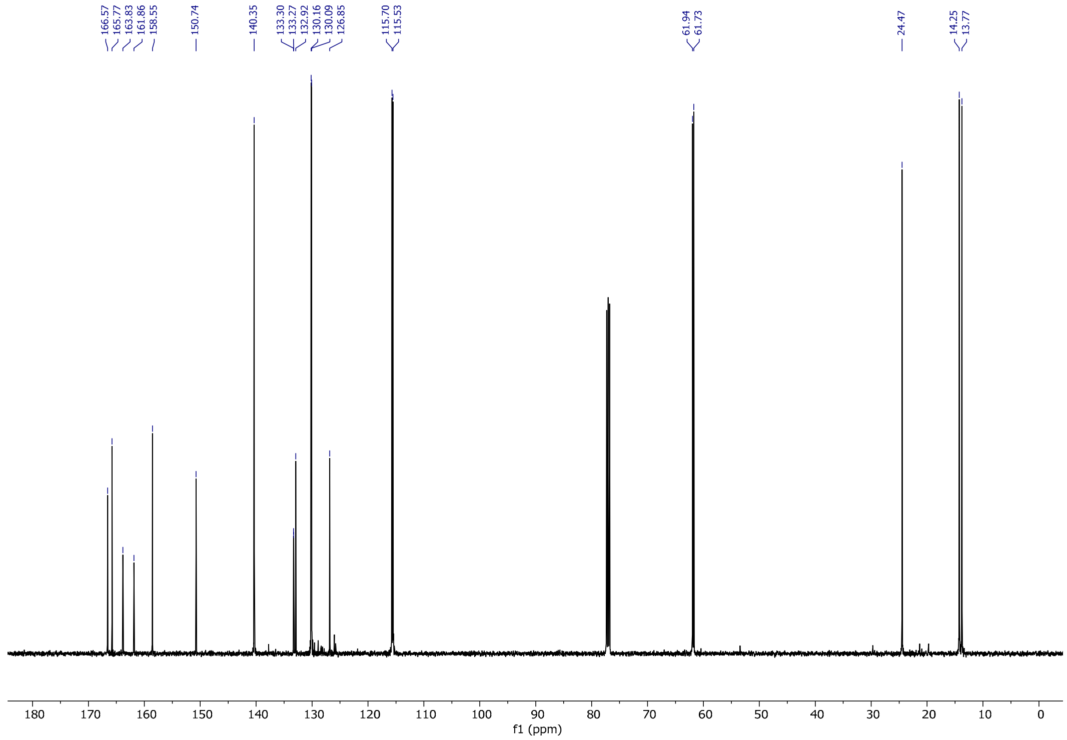

Figure S43. ^{1}H-NMR (500 MHz, $CDCl_3$) of compound **4c**

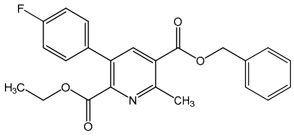

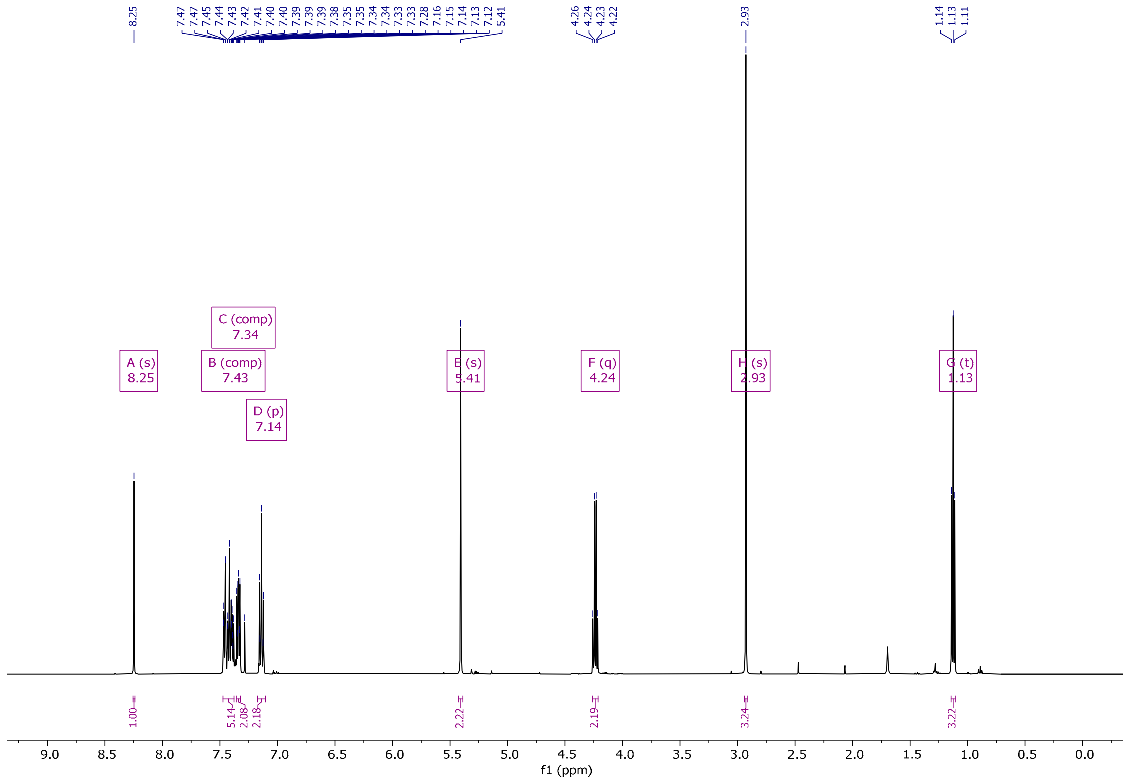

Figure S44. ^{13}C-NMR (126 MHz, $CDCl_3$) of compound **4c**

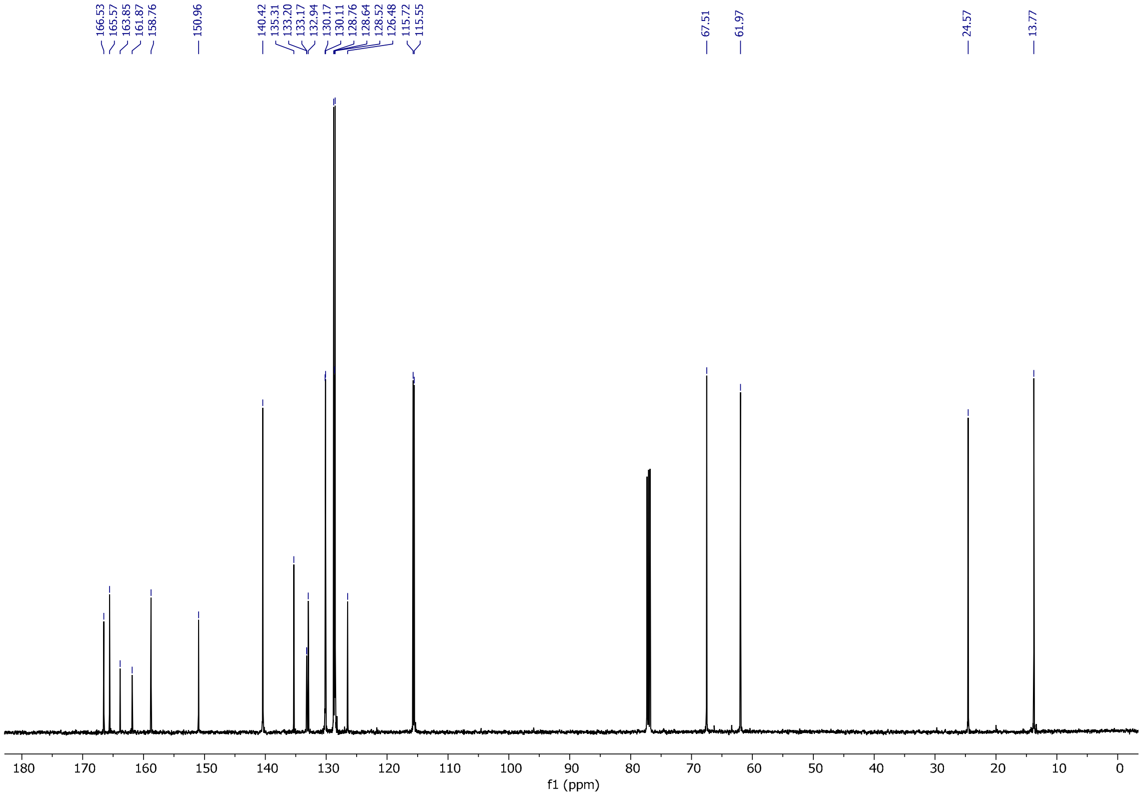

Figure S45. ^{1}H-NMR (500 MHz, $CDCl_3$) of compound **5c**

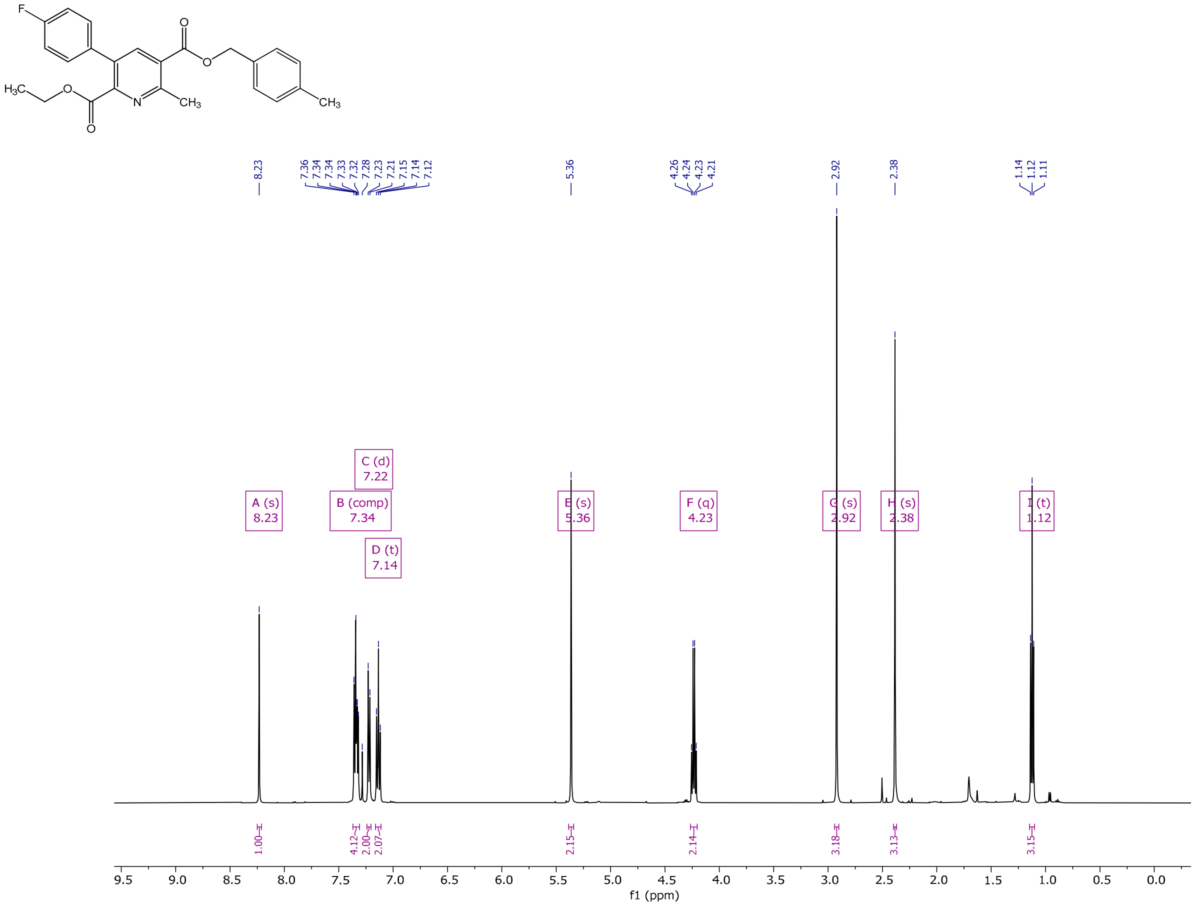

Figure S46. ^{13}C-NMR (126 MHz, $CDCl_3$) of compound **5c**

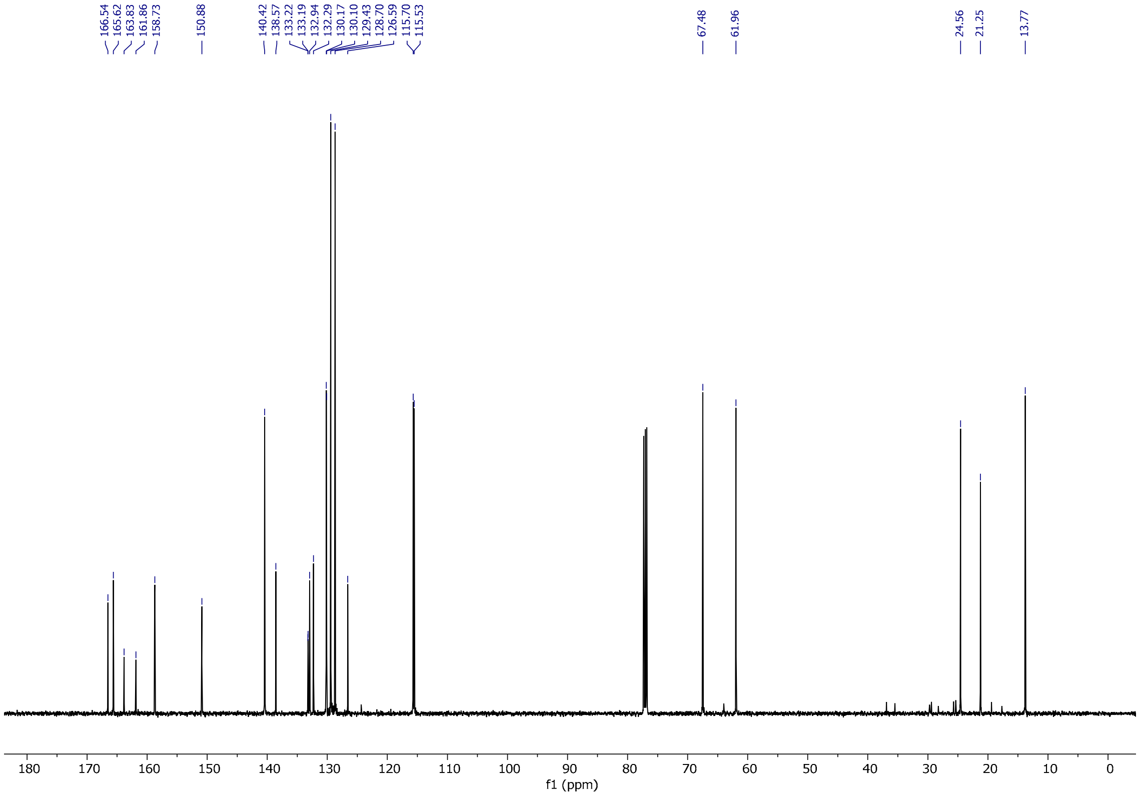

Figure S47. ^{1}H-NMR (500 MHz, $CDCl_3$) of compound **6c**

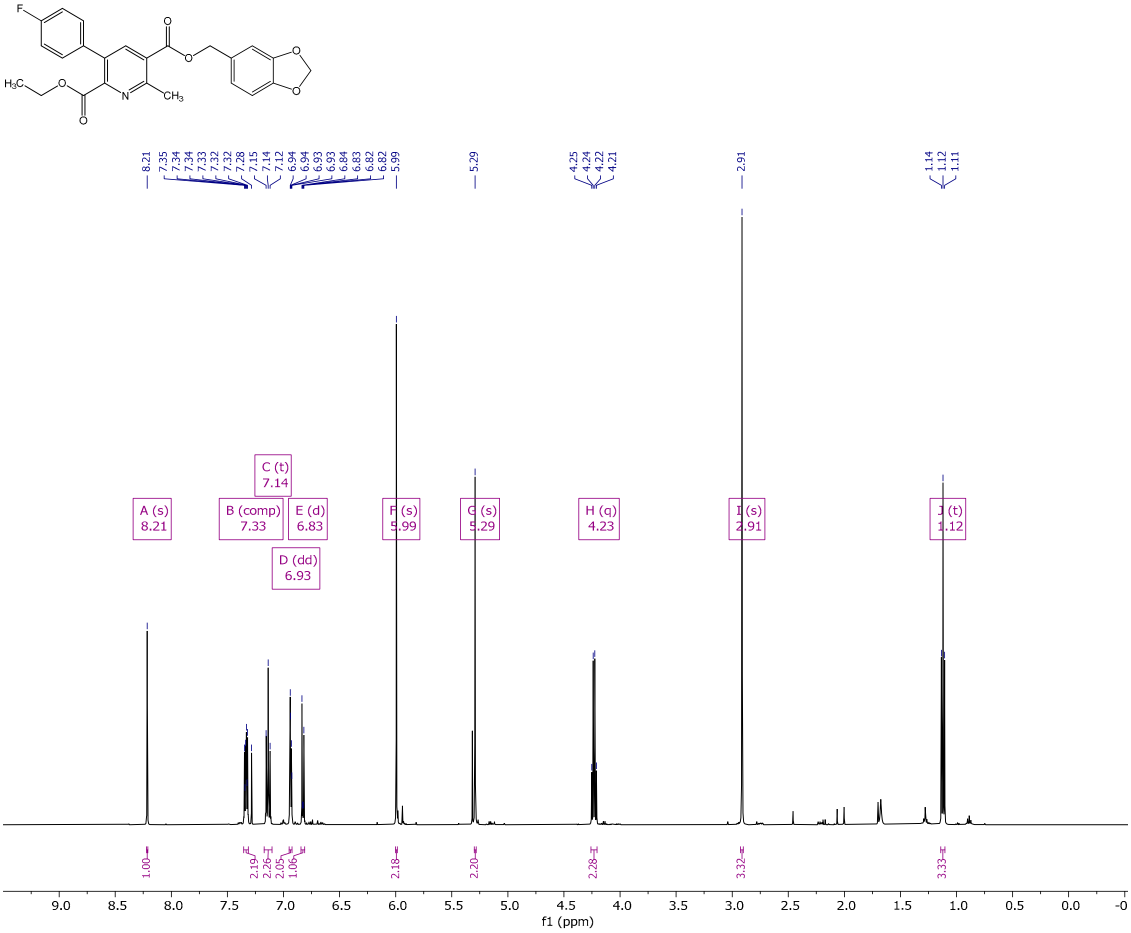

Figure S48. ^{13}C-NMR (126 MHz, $CDCl_3$) of compound **6c**

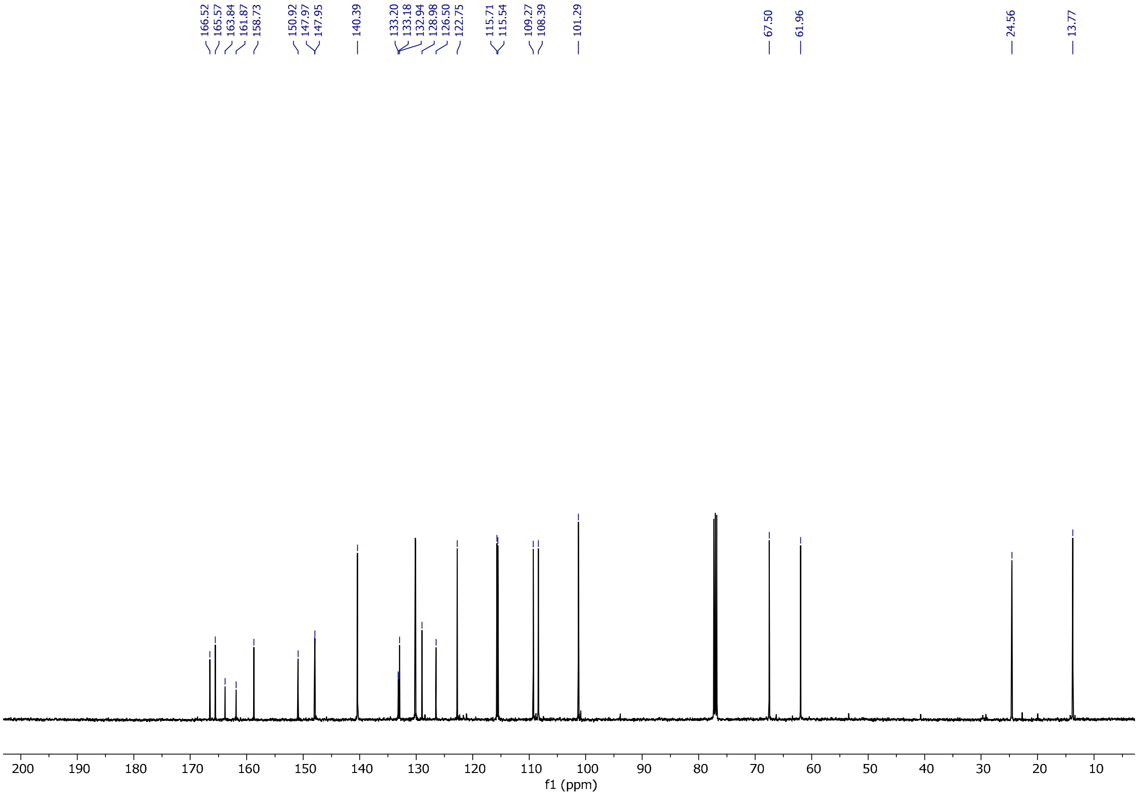

Figure S49. ^{1}H-NMR (500 MHz, $CDCl_3$) of compound **7c**

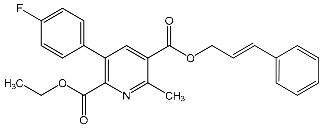

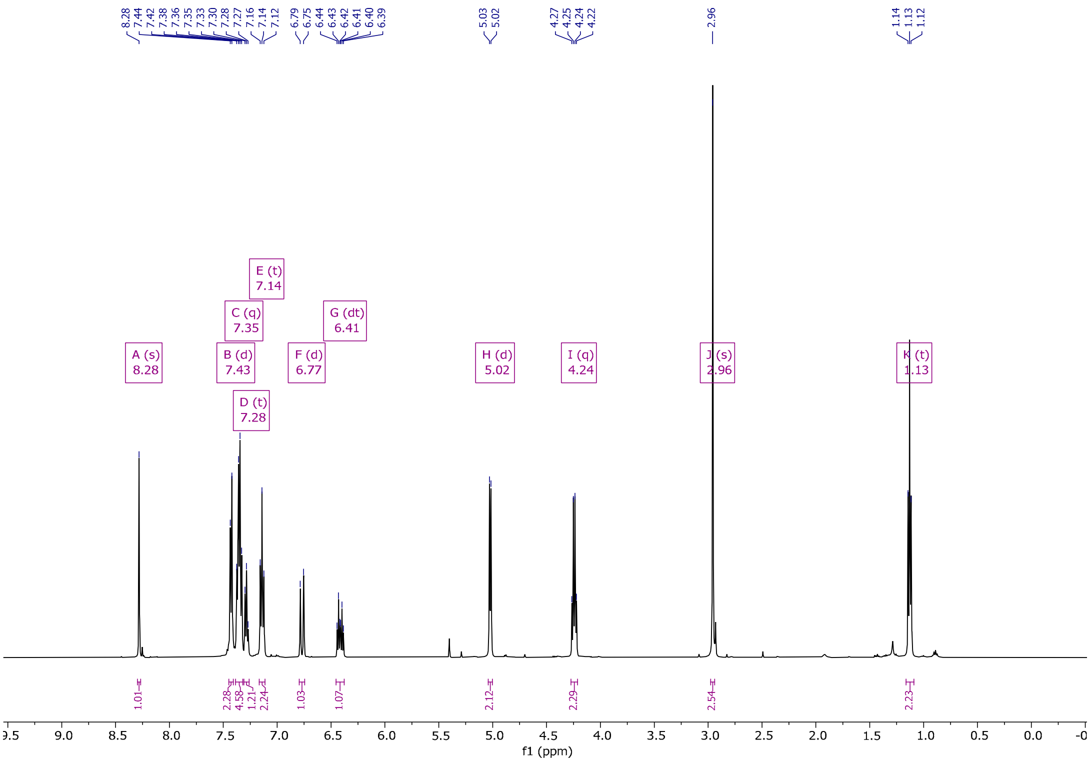

Figure S50. ^{13}C-NMR (126 MHz, $CDCl_3$) of compound **7c**

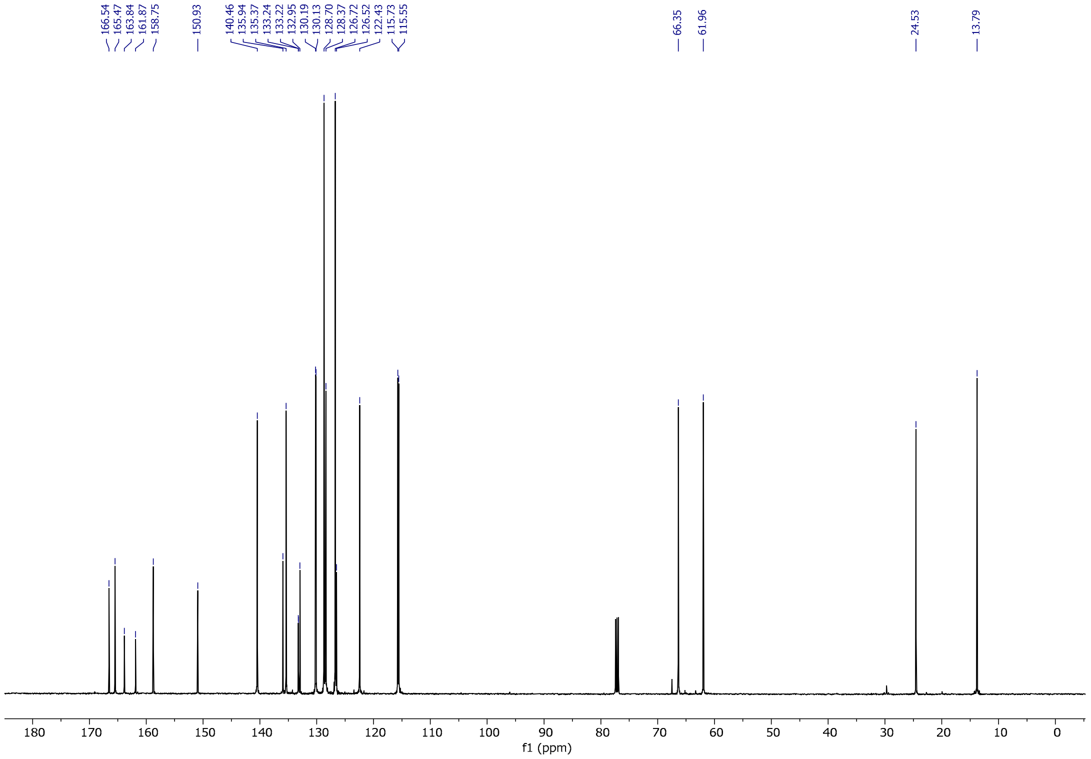

Figure S51.^{1}H-NMR (500 MHz, $CDCl_3$) of compound **8c**

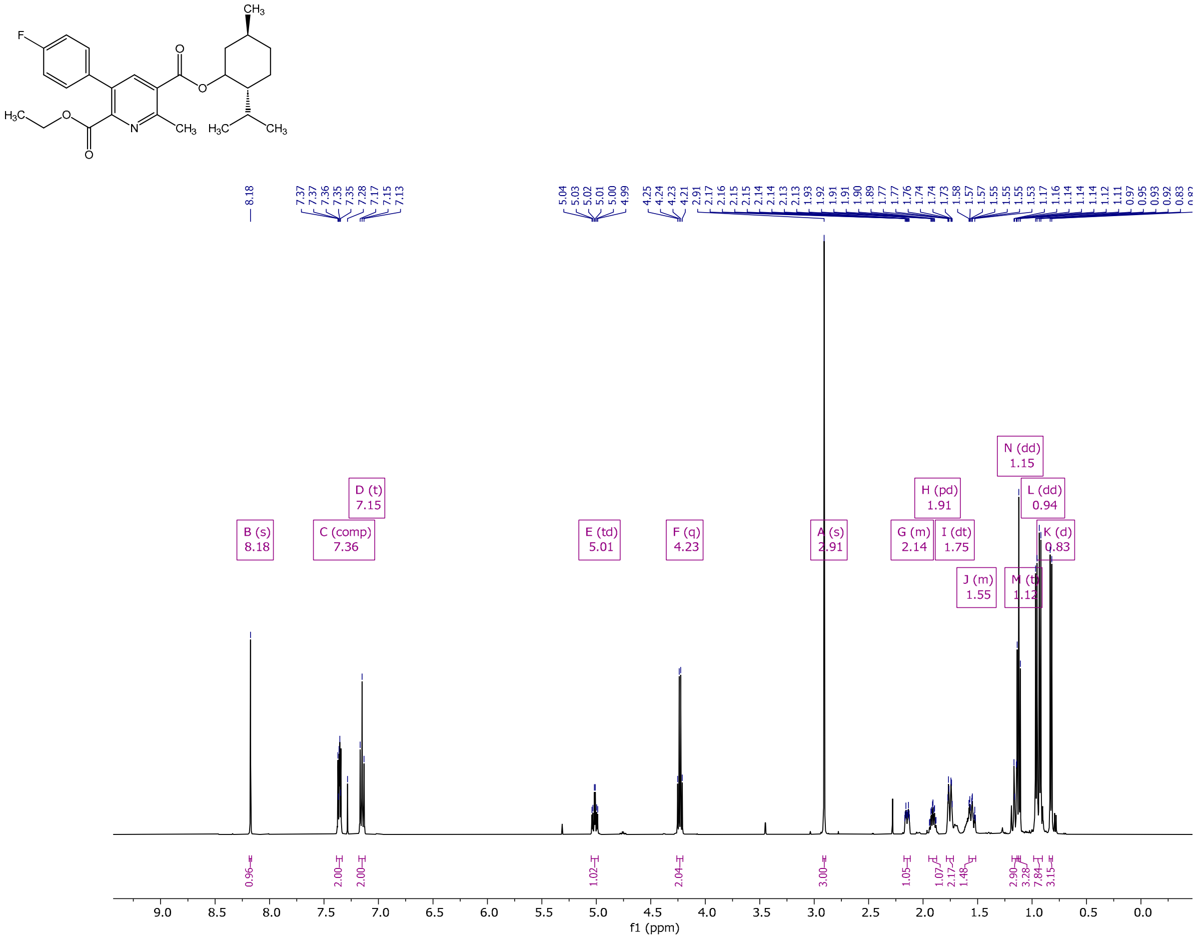

Figure S52. ^{13}C-NMR (126 MHz, $CDCl_3$) of compound **8c**

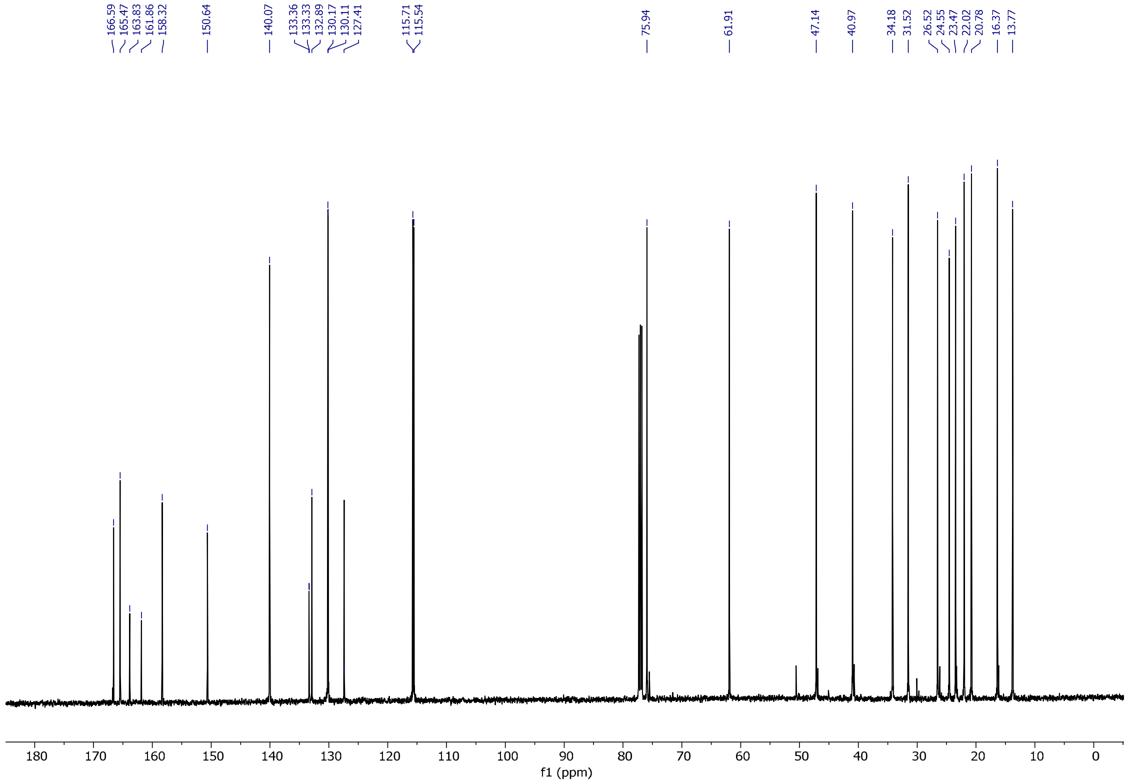

Figure S53. ^{1}H-NMR (500 MHz, $CDCl_3$) of compound **9c**

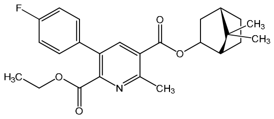

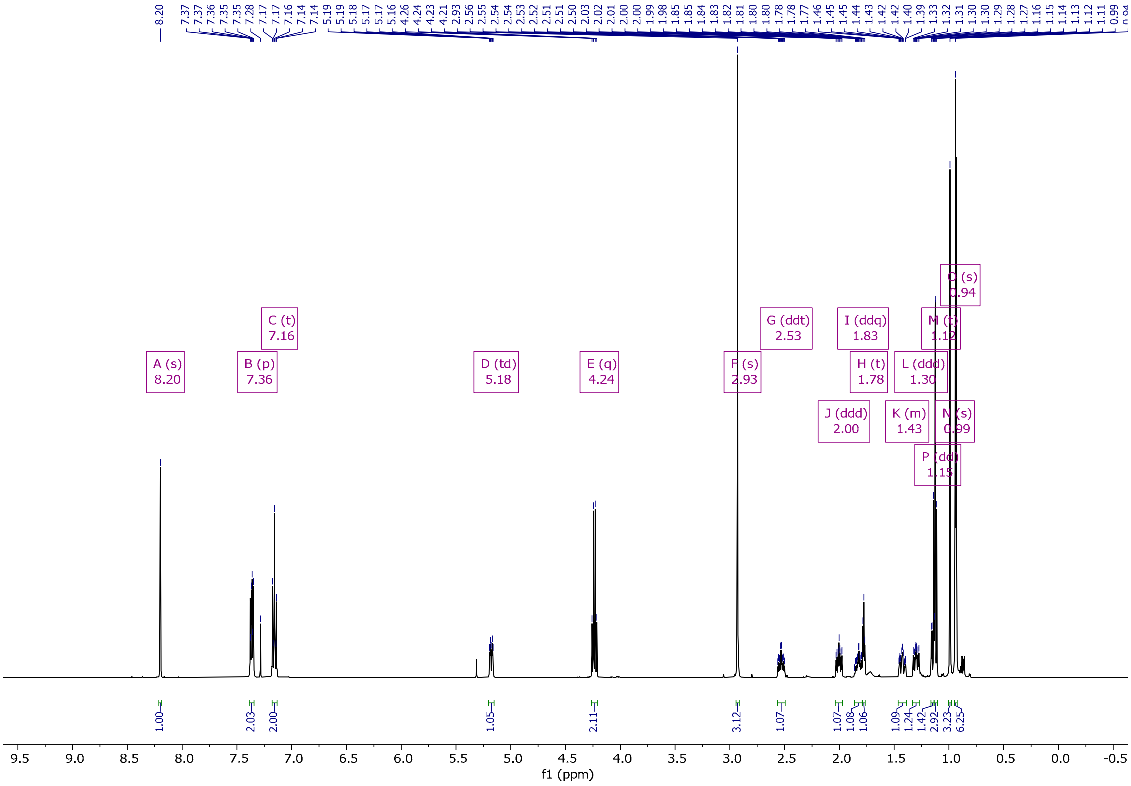

Figure S54. ^{13}C-NMR (126 MHz, $CDCl_3$) of compound **9c**

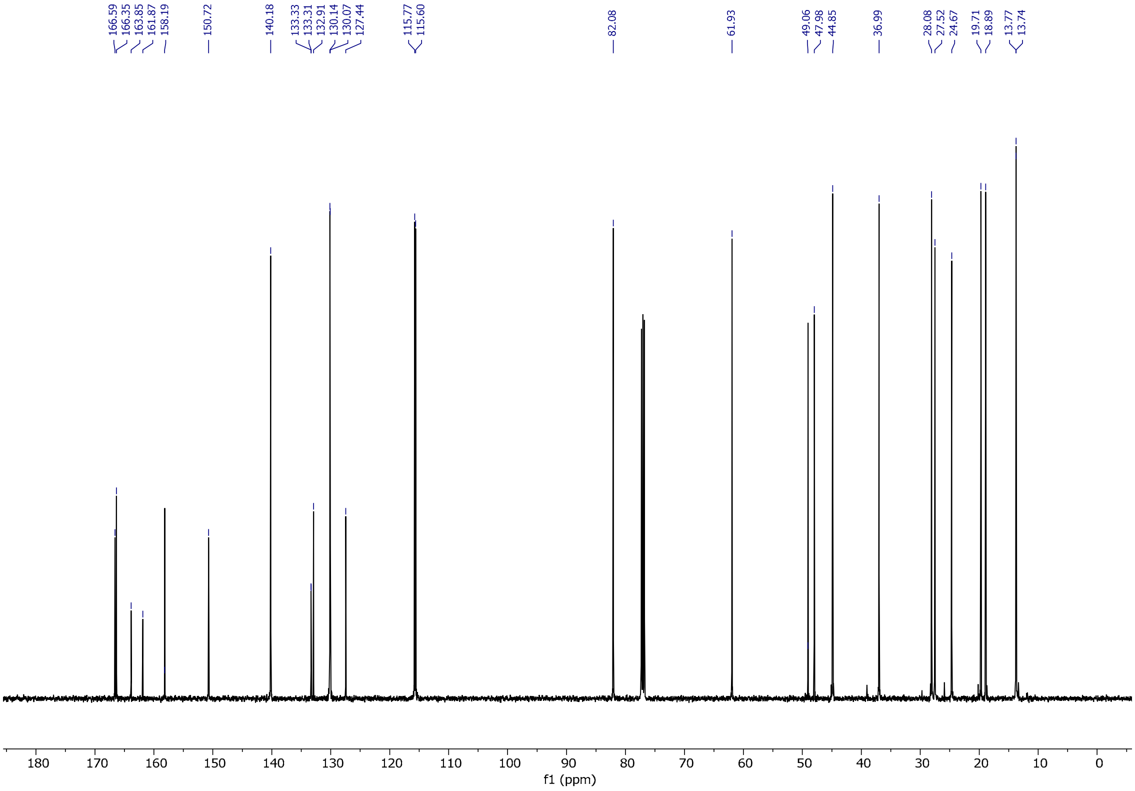

Figure S55. ^{1}H-NMR (500 MHz, $CDCl_3$) of compound **10c**

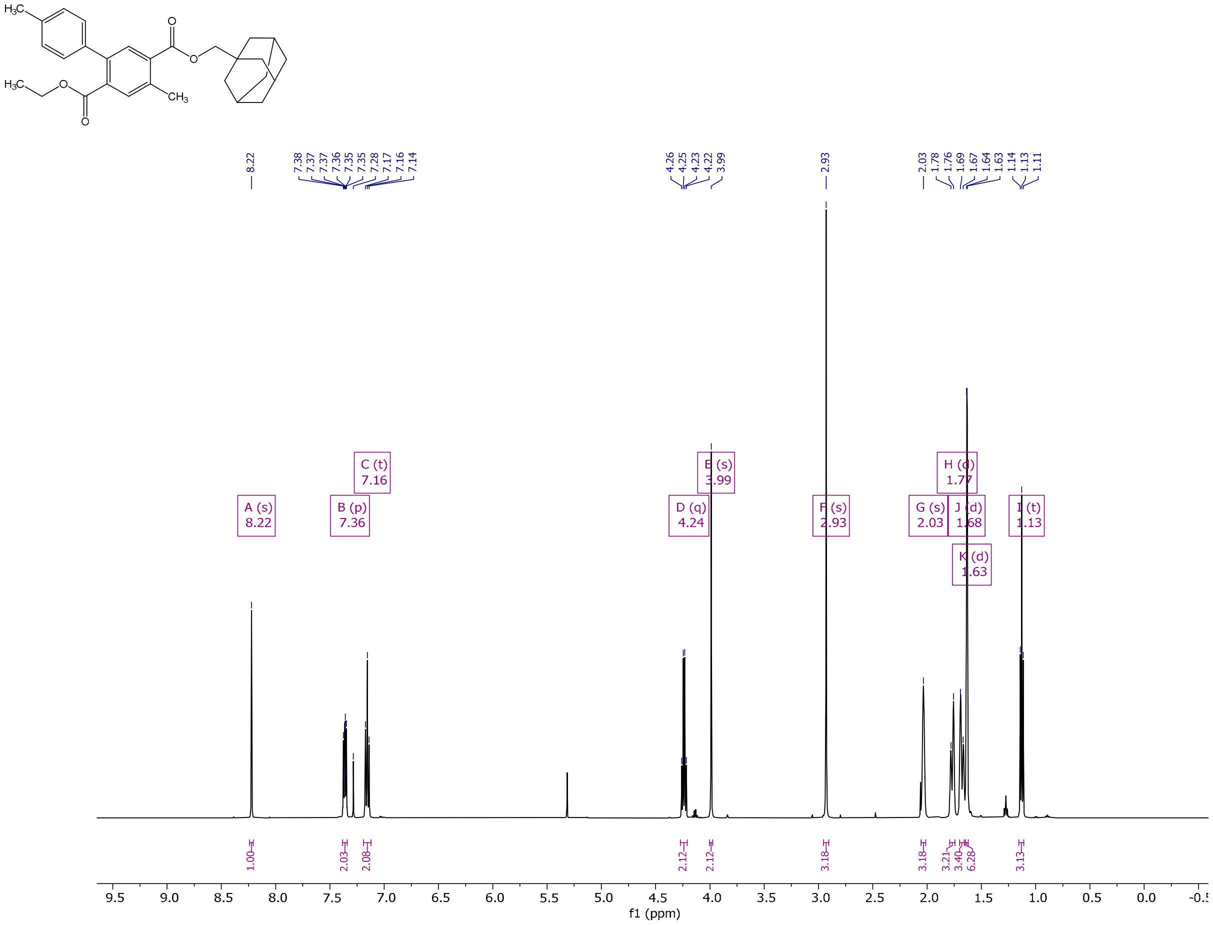

Figure S56. ^{13}C-NMR (126 MHz, $CDCl_3$) of compound **10c**

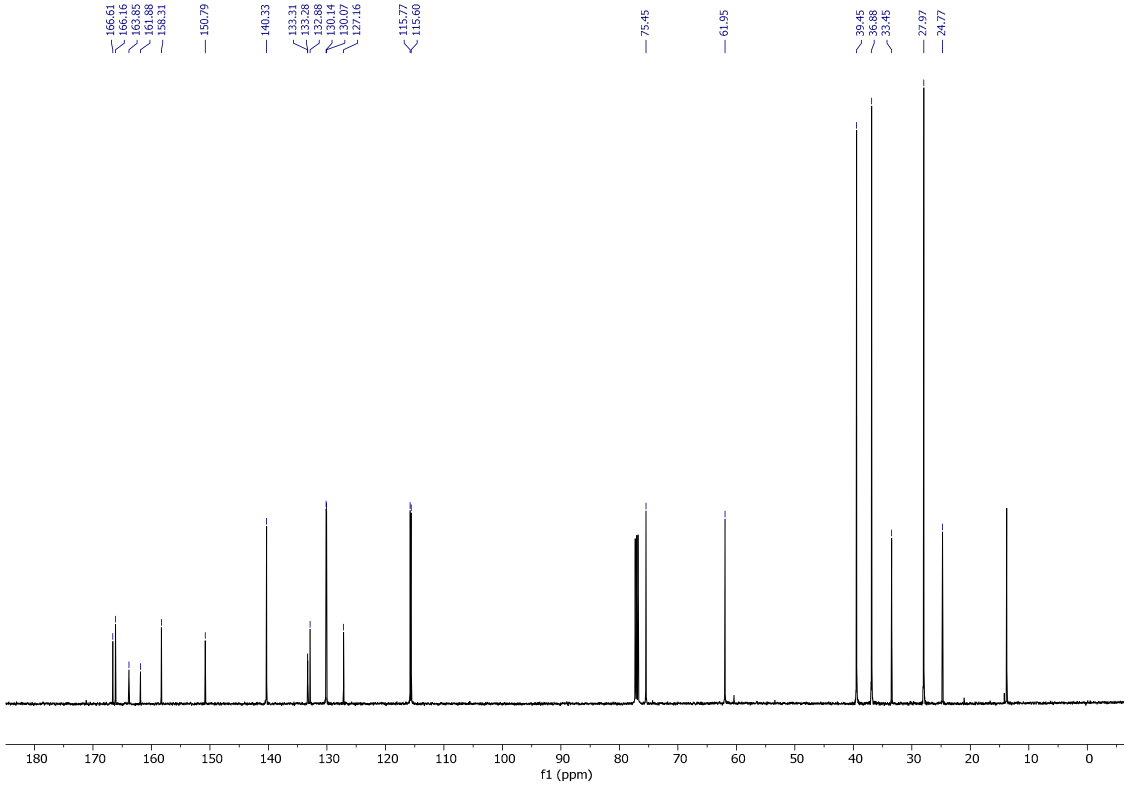

Figure S57. ^{1}H-NMR (500 MHz, $CDCl_3$) of compound **11c**

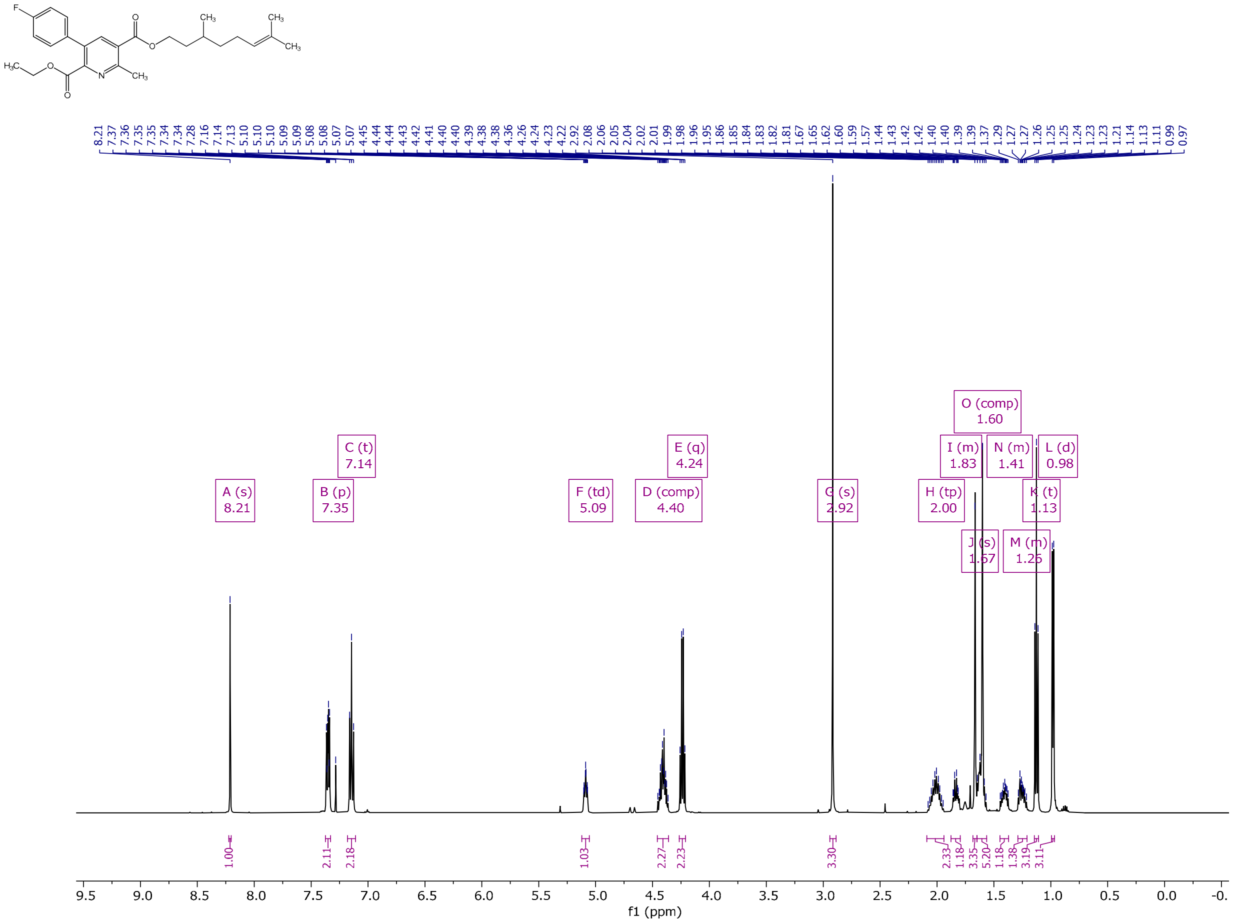

Figure S58. ^{13}C-NMR (126 MHz, $CDCl_3$) of compound **11c**

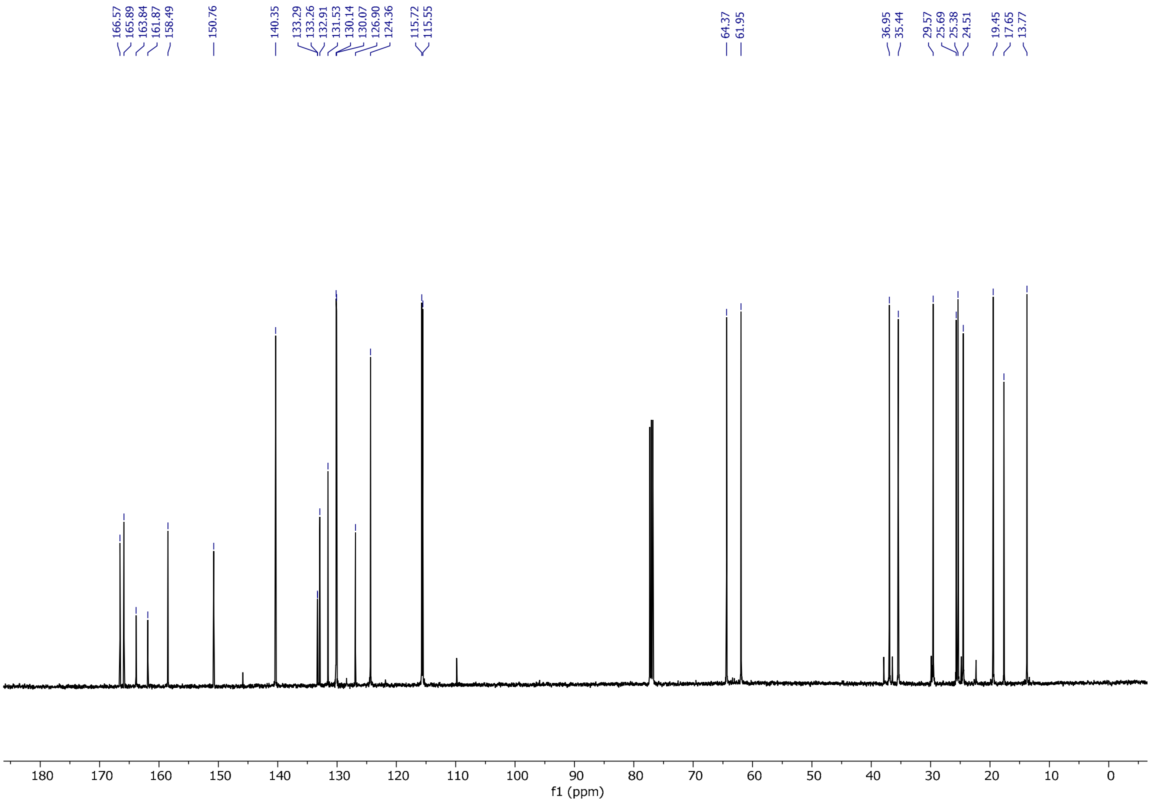

Figure S59. ^{1}H-NMR (500 MHz, $CDCl_3$) of compound **12c**

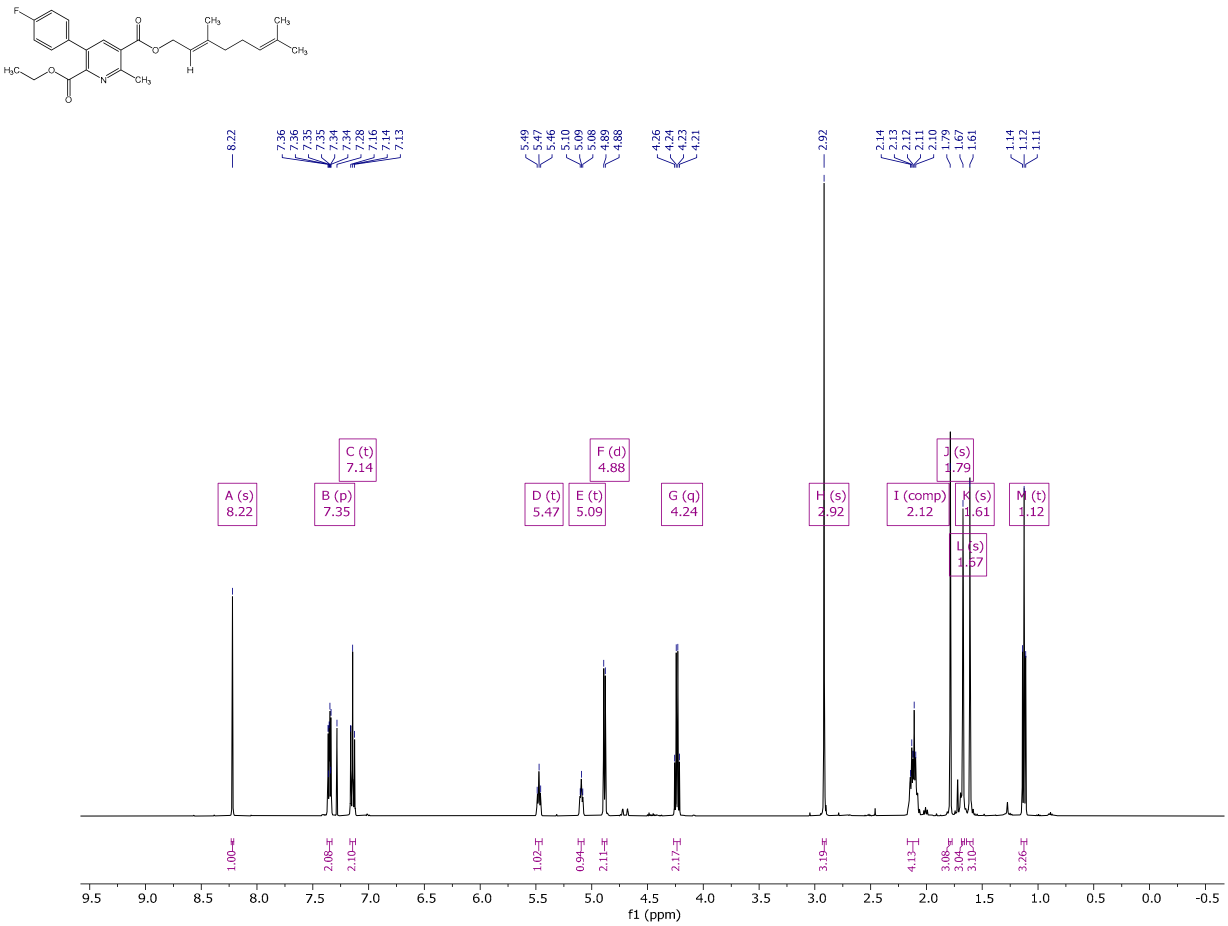

Figure S60. ^{13}C-NMR (126 MHz, $CDCl_3$) of compound **12c**

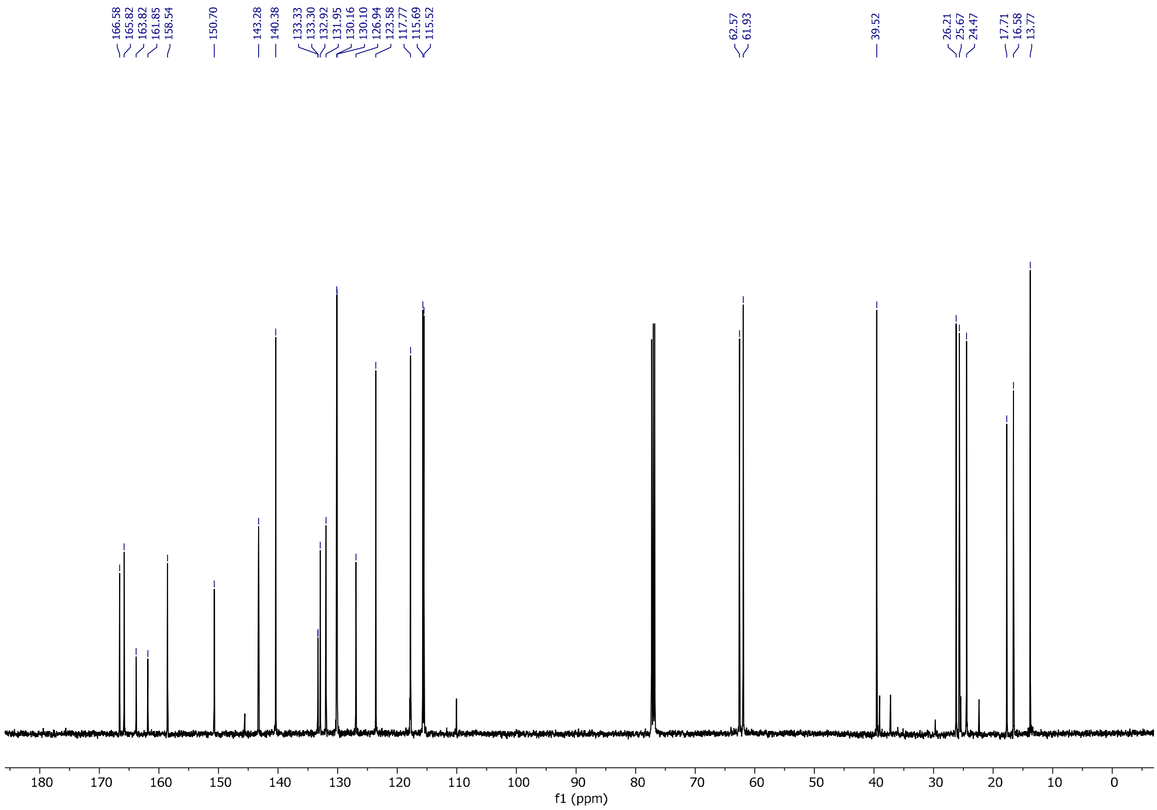

3. Crystallographic information

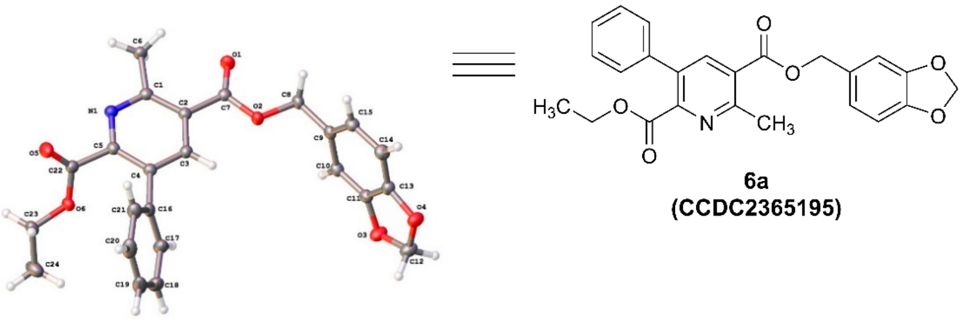

Figure S61. ORTEP drawing of the molecular structure of 6a. Identification code: Hpd915; Empirical formula: $C_{24}H_{21}NO_6$; Formula weight: 419.42; Crystal system: Triclinic; Space group: P-1; a (Å): 6.6874(2); b (Å): 11.2712(4); c (Å): 14.7436(4); α (°): 73.294(3); β (°): 81.513(3); γ (°): 75.161(3); Volume (Å^3): 1025.61(6); Z: 2; ϱ (calc.): 1.358; λ: 1.54184; Temp. (K): 100.0(1); F(000): 440.

Single crystals of **6a** (CCDC2365195) were prepared by slow evaporation of a methylene chloride solution. A suitable colorless plank-like crystal, with dimensions of 0.133 mm × 0.077 mm × 0.046 mm, was mounted in paratone oil onto a nylon loop. All data were collected at 100.0(1) K, using a XtaLAB Synergy/ Dualflex, HyPix fitted with CuKα radiation (λ = 1.54184 Å). Data collection and unit cell refinement were performed using CrysAlisPro software[5]. The total number of data were measured in the 6.3° < 2θ < 153.0° using ω scans. Data processing and absorption correction, giving minimum and maximum transmission factors (0.791, 1.000) were accomplished with CrysAlisPro[1] and SCALE3 ABSPACK[6], respectively. The structure, using Olex2[7], was solved with the ShelXT[8] structure solution program using direct methods and refined (on F^2) with the ShelXL[9] refinement package using full-matrix, least-squares techniques. All non-hydrogen atoms were refined with anisotropic displacement parameters. All hydrogen atom positions were determined by geometry and refined by a riding model.
